# Structure and Bonding of Halonium Compounds

**DOI:** 10.1021/acs.inorgchem.3c00654

**Published:** 2023-05-31

**Authors:** Juan D. Velasquez, Jorge Echeverría, Santiago Alvarez

**Affiliations:** † Instituto de Síntesis Química y Catálisis Homogénea (ISQCH) and Departmento de Química Inorgánica, Facultad de Ciencias, Universidad de Zaragoza, Pedro Cerbuna 12, 50009 Zaragoza, Spain; ‡ Departament de Química Inorgànica i Orgànica and Institut de Química Teòrica i Computacional (IQTC-UB), Universitat de Barcelona, Martí i Franquès 1-11, 08028 Barcelona, Spain

## Abstract

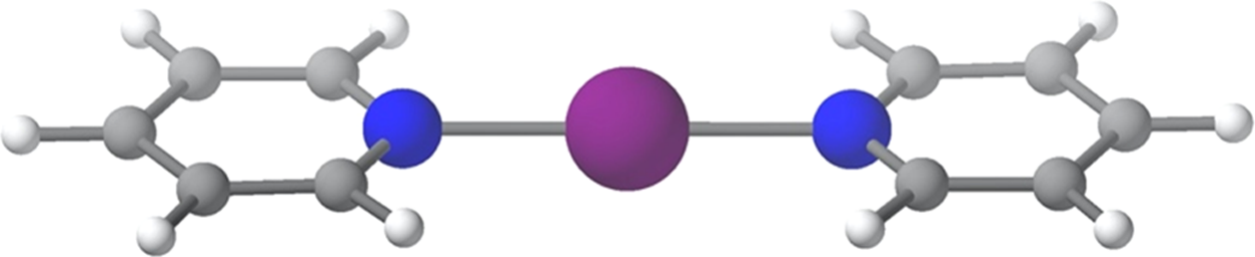

The geometrical parameters and the bonding in [D···X···D]^+^ halonium compounds, where D is a Lewis base with N as the donor atom and X is Cl, Br, or I, have been investigated through a combined structural and computational study. Cambridge Structural Database (CSD) searches have revealed linear and symmetrical [D···X···D]^+^ frameworks with neutral donors. By means of density functional theory (DFT), molecular electrostatic potential (MEP), and energy decomposition analyses (EDA) calculations, we have studied the effect of various halogen atoms (X) on the [D···X···D]^+^ framework, the effect of different nitrogen-donor groups (D) attached to an iodonium cation (X = I), and the influence of the electron density alteration on the [D···I···D]^+^ halonium bond by variation of the R substituents at the N-donor upon the symmetry, strength, and nature of the interaction. The physical origin of the interaction arises from a subtle interplay between electrostatic and orbital contributions (σ-hole bond). Interaction energies as high as 45 kcal/mol suggest that halonium bonds can be exploited for the development of novel halonium transfer agents, in asymmetric halofunctionalization or as building blocks in supramolecular chemistry.

## Abstract 2

The geometry, strength, and physical nature of the bonding in halonium cationic compounds have been comprehensively investigated by means of a combined structural and computational analysis.

## Introduction

**Scheme 1:**
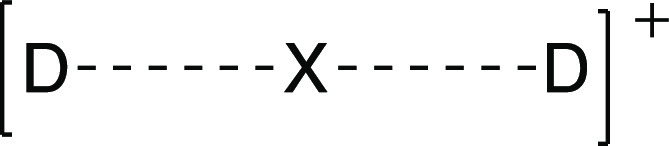
Structure of a Halonium Cation (X = Halogen, D = Neutral Lewis Base)

Halogen bonds are highly directional noncovalent interactions between a nucleophilic Lewis base (D) and the electrophilic region of a polarized halogen atom (X).^1^ In terms of geometry, bond strength, and origin of the interaction, halogen bonds are similar to hydrogen bonds.^2−7^ In some cases, both interactions are simultaneously found, either competing^8^ or cooperating.^9,10^ Like tetrel,^11^ pnicogen,^12^ and chalcogen^13^ bonds, the nature of the interaction can be rationalized in terms of a σ-hole^14,15^ with non-negligible charge transfer, dispersion, and polarization contributions.^4−7,16^ In contrast to the classical two-center halogen bond, R–X···D, in which a covalently bonded neutral halogen interacts with a Lewis base (D), the electron-deficient halonium ions (X^+^) tend to interact simultaneously with two Lewis bases. The resulting linear three-center bond, [D···X···D]^+^, is shown in Scheme 1.^17^

**Figure 1:**
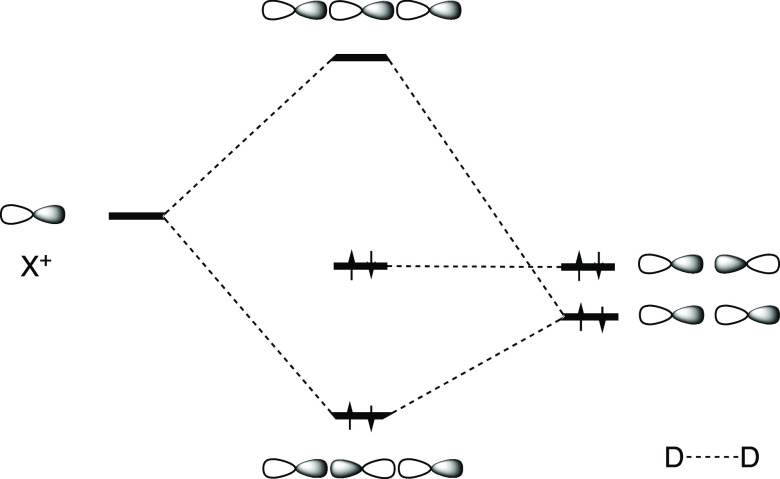
Molecular orbital diagram for the halonium bond, [D···X···D]^+^, for X = Cl, Br, and I.

The bonding in halonium ions (X = Cl, Br, and I) leads to a hypercoordinated system. These stable^18−20^ noncovalent complexes with short interatomic distances, in which the central atom exceeds the octet rule, have attracted interest due to their applicability as synthetic reagents^17,21^ and in the design of complex supramolecular synthons^17,22−28^ and two-dimensional (2D) halogen-bonded organic framework (XOF) materials.^29−31^ The nature of the interaction in halonium cations can be described in terms of orbital and electrostatic contributions,^32−36^ with smaller contribution of dispersion forces.^33^ According to the Pimentel–Rundle model,^37−39^ the halonium cation interacts simultaneously with two Lewis bases by accepting electrons through both lobes of its empty p-orbital. Consequently, three atomic orbitals combine to form three molecular orbitals (Figure 1). Two electrons are in the bonding orbital and two in the nonbonding orbital, while the antibonding orbital remains unfilled.^17,19^ Notice that this simplified model does not take into account the interaction between the occupied s(X) orbital and the symmetric combination of the two donor orbitals, which introduces significant Pauli repulsion.

Alternatively, one could consider a halonium ion as resulting from the interaction between two closed-shell groups, D–X^+^ and D, to which the σ-hole formalism could be applied, since the anisotropic electron distribution of D–X^+^ forms a σ-hole, produced by the presence of an empty D–X antibonding orbital opposite to that bond, that can establish Coulombic and orbital interactions with the electron density of the lone pair of the incoming donor. Since the σ-hole originates from the lobe of the empty p-orbital of the cationic halogen atom, X^+^, Hakkert has proposed that the two partially positively charged regions of X^+^ may best be termed as p-holes.^32^ According to Ruedenberg’s seminal work, the formation of chemical bonding can be alternatively explained in terms of the lowering of the kinetic energy associated with electron delocalization upon bond formation.^40^

Notice that a trihalide anion, X_3_^–^, can be considered as a central halogen cation bonded to two terminal halides, X^–^···X^+^···X^–^, the main difference with halonium cations being that the whole assembly is in this case negatively charged.^41^ Unsurprisingly, the MO diagram for the trihalides^42^ is identical to that of the halonium cations (Figure 1). In a similar way, X_3_^–^ can also be described as X_2_ and X^–^ interacting units that could be rationalized by the σ-hole model.^43^

In the case of the fluoronium complexes, [D···F···D]^+^, computational studies for D = pyridine suggest that they are best described as [D–F]^+^···D ion–molecule complexes^34^ since they prefer an asymmetric geometry with one classical covalent bond (1.360 Å) and a second, weaker and longer halogen bond (3.499 Å).^33^ Indeed, to form [D···F···D]^+^ adducts, a highly electron-withdrawing group directly attached to the F center is required, thereby enhancing its σ-hole.^17^

When D is an N-donor, [N···I···N]^+^ complexes are symmetric and static in both the solid state and in solution,^19,33,35^ regardless of the solvent polarity^44^ and of the size, charge distribution, or coordination strength of the counterion.^45^ The effect of the substituents on the electron density of the [N···I···N]^+^ halonium bond was assessed upon symmetric modulation of the *para*-positions of [bis(4-R-pyridine)iodine]^+^ model^17,46^ and the geometrically restrained [1,2-bis((4-R-pyridine-2-ylethynyl)benzene)iodine]^+^ complex.^46^ To the best of our knowledge, no attempts at analyzing the impact of the electron density alteration on the strength and nature of the bond have been reported when the R groups are located at *ortho-*, double *ortho-*, *meta-*, and double *meta*-positions relative to the pyridine nitrogen.

**Figure 2:**
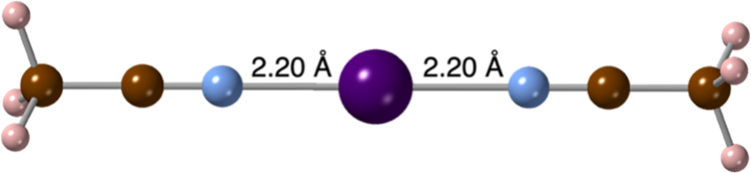
Short I···N contacts in the crystal structure of bis(acetonitrile)-iodonium cation.^47^ Color code: pink, H; brown, C; blue, N.; and purple, I.

**Scheme 2:**
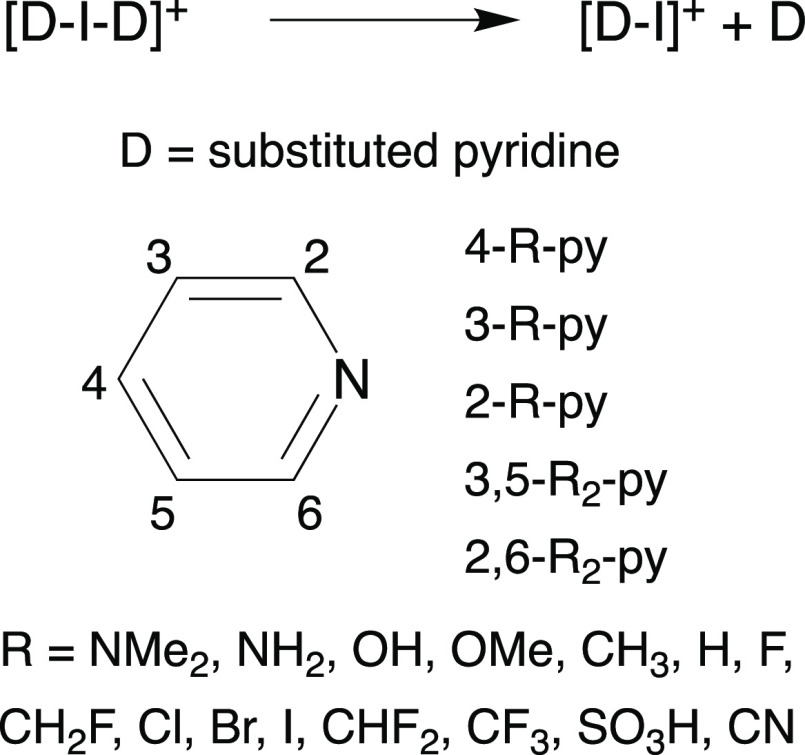
Heterolytic Cleavage Reactions Considered in This Study

Herein, we present a combined structural and computational study of the [N···X···N]^+^ halonium bond. By means of density functional theory (DFT) calculations, the geometry and strength of the halonium bond were studied from three different viewpoints in the present work: (1) the effect of various halogen atoms (X) on the [py···X···py]^+^ framework, (2) the effect of different nitrogen-donor groups (D) attached to the iodonium cation and (3) the influence of the electron density alteration on the [N···I···N]^+^ halonium bond by variation of the R substituents at the N-donor. The MN12-SX method was selected after an extensive benchmark study on the performance of 11 common DFT functionals and the corresponding dispersion-corrected functionals, as well as with the second-order Møller–Plesset perturbational method (MP2), in predicting the geometry and interaction energy of the bis(acetonitrile)-iodonium cation (BUKNAX,^47^Figure 2). The results were compared with the experimental and calculated data at the coupled cluster singles and doubles (CCSD) and Perturbative Triple excitations (CCSD(T)) level (see Supporting Information, Table S2). The covalent vs dative character of the X···N bond in the bis-pyridine halonium cations was discussed by Georgiou and co-workers using both theoretical and synthetic techniques. They concluded that the removal of the “first” pyridine is clearly heterolytic, both in the gas phase and in the presence of solvent dielectric fields.^34^ In light of these results, we have focussed our analysis on the heterolytic dissociation of several halonium ions (Scheme 2). To further investigate the nature of the interaction and the factors that affect its strength we have performed molecular electrostatic potential (MEP) and energy decomposition analyses (EDA) of the same compounds (Scheme 2). Since the strength of the electrostatic interaction in the halonium bond is related to the positive value of the electrostatic potential (*V*_s,max_) at the σ-hole of [D–X]^+^ and the anisotropic distribution of charge around the halogen atom,^48^ we have paid special attention to possible correlations between the value of *V*_s,max_ and computed geometrical and/or energetic descriptors.

## Structural Analysis

We have searched the Cambridge Structural Database (CSD)^49^ for compounds containing a [N···X···N] central framework with X···N bonds or contacts shorter than the sum of the van der Waals radii. In our searches, the central atom X was set to be I, known to form linear and highly symmetric three-center-four-electron bonds.^47,50−52^

**Figure 3:**
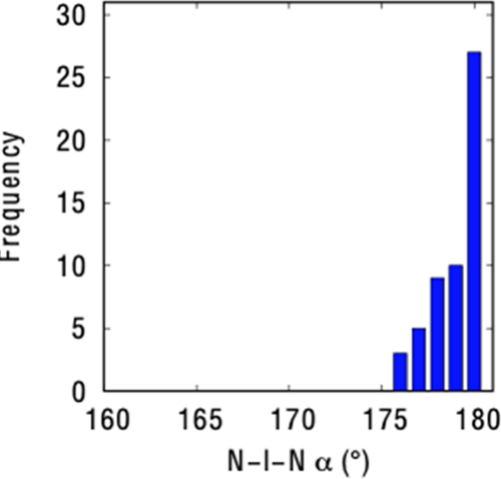
N–I–N angle distribution for compounds containing a [N···I···N] central framework.

Only linear [N···I···N]^+^ halonium systems with neutral donors (Figure 3) have been reported in the CSD database, all having angles between 175 and 180°. This result is consistent with the proposal that the central atom employs an empty atomic p-orbital to interact with the N lone pairs of the two donors and thereby give rise to practically linear [N···I···N] frameworks (Figure 1). Altogether, 37 crystal structures were found, 1 with an sp nitrogen atom attached to the iodonium cation (BUKNAX),^47^ 35 with sp^2^ nitrogen, and 1 with sp^3^ nitrogen atoms (HMTITI).^50^ Among the sp^2^ nitrogen compounds, three are nonaromatic in an R_3_P=N–I phosphazene moiety (HINXIL,^51^ HINXOR,^51^ and KABRUB^52^). The average I···N distance in these structures is 2.26 Å and the average N···I···N angle is 179°. The difference of less than 3% between the two I···N bond lengths is within chemical accuracy. Thus, the complexes show an overall symmetric [N···I···N]^+^ geometry in the solid state. The shortest contact is found for BUKNAX^47^ (Figure 2), with two identical I···N distances of 2.20 Å, i.e., 1.5 Å shorter than the sum of the van der Waals radii (3.70 Å),^53^ or an interpenetration of the van der Waals crusts of 94%, consistent with a hypercoordinated bond.^54^

## Analysis of the Molecular Electrostatic Potential

### Effect of the Central Atom X

**Figure 4:**
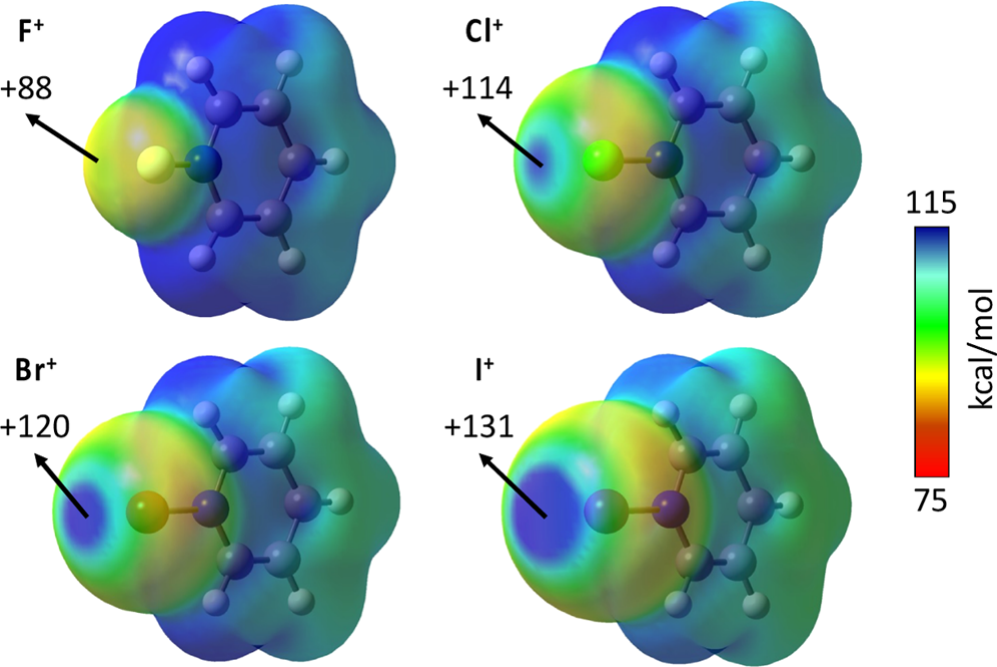
MEP maps for [X···NC_5_H_5_]^+^ cations (X = F, Cl, Br, and I) calculated at the MN12-SX/def2-TZVP level and plotted on the electron density van der Waals isosurface (*s* = 0.001 Å). Energies are given in kcal/mol. Red and blue colors indicate the least and most positive MEP values, respectively.

Figure 4 shows the MEP maps of the [X–NC_5_H_5_]^+^ cations (X = F, Cl, Br, and I). These systems were selected to evaluate the effect of the halogen atom on the magnitude of the σ-hole.

Upon initial inspection, all compounds exhibit a σ-hole close to the central atom and opposite to the N–X bond and the maximum electrostatic potential value (*V*_s,max_) of the σ-hole increases when descending down the halogen group. All in all, the MEP maps allow us to explain the structural preferences found in the previous section. Halogen(I) compounds (X = Cl, Br, and I) would form highly linear [N···X···N] frameworks since the interaction with the Lewis base along the X···N axis is favored by the Coulombic attraction. Regarding fluor(I) compounds, its small σ-hole might be the cause for the asymmetric arrangement (*d*_N···F_ = 1.337 and 2.790 Å; α_N–F···N_ = 179.0°) of the optimized fluoronium compound, [py-F···py]^+^, similar to a classical halogen bond. For that reason, we will not consider the F center in the following discussion.

### Effect of the Donor

**Figure 5:**
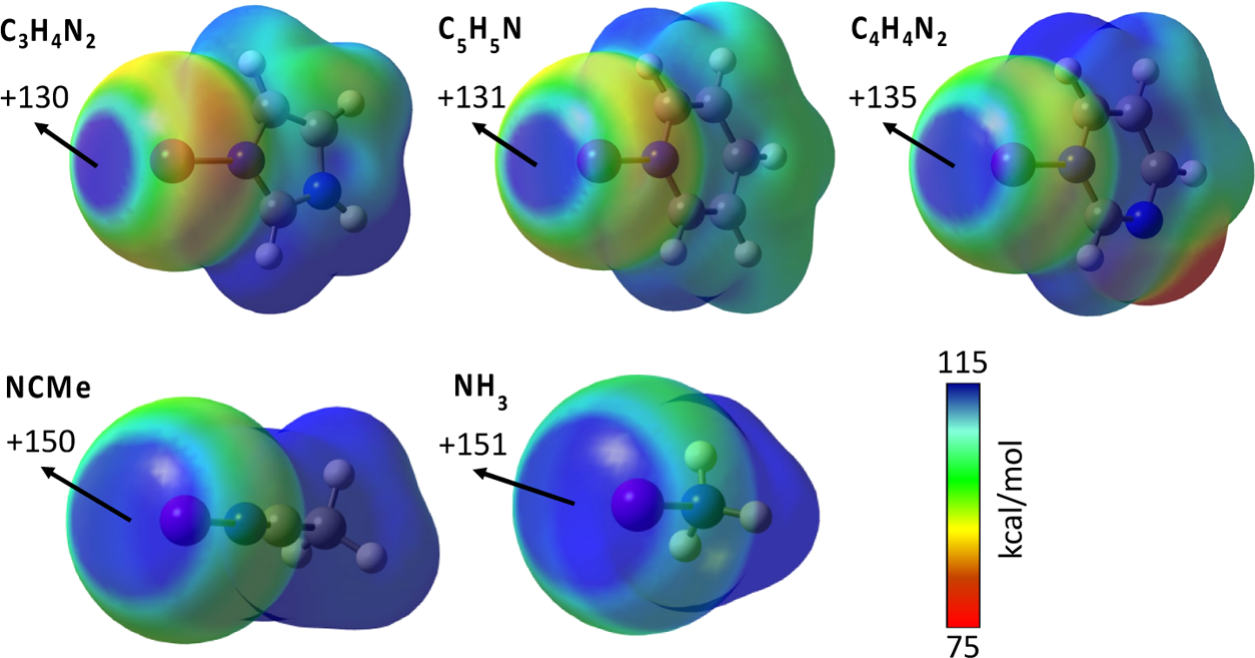
MEP map for [I···D]^+^ cations (D = C_3_H_4_N_2_, C_5_H_5_N, C_4_H_4_N_2_, NCMe, and NH_3_) calculated at the MN12-SX/def2-TZVP level and plotted on the electron density van der Waals isosurface (*s* = 0.001 Å). Energies are given in kcal/mol. Red and blue colors indicate less and more positive MEP values, respectively.

Figure 5 shows the MEP maps of [I···D]^+^ complexes, where D is imidazole (C_3_H_4_N_2_), pyridine (C_5_H_5_N), pyrimidine (C_4_H_4_N_2_), acetonitrile (NCMe), and ammonia (NH_3_).

Different nitrogen-containing donors (D) have been selected to study their effect on the magnitude of the σ-hole associated with the I···N bond axis (we kept the iodonium cation as the reference central atom X because it showed the most marked σ-hole among the halonium ions). Upon changing from pyridine (C_5_H_5_N) to other aromatic donors such as imidazole (C_3_H_4_N_2_) and pyrimidine (C_4_H_4_N_2_), the *V*_s,max_ value of the σ-hole changes very little. In contrast, it increases with nonaromatic donors such as ammonia (NH_3_) and acetonitrile (NCMe).

### Effect of the R Group

The [py···I]^+^ complex has been used as a reference to analyze the influence of the electron density alteration on the I···N σ-hole induced by variation of the R substituents attached in *ortho* (2-R-py and 2,6-R_2_-py), *meta* (3-R-py and 3,5-R_2_-py) and *para*-positions (4-R-py) relative to the pyridine nitrogen. Several substituents have been studied (R = NMe_2_, NH_2_, OH, OMe, CH_3_, CH_2_F, CHF_2_, CF_3_, F, Cl, Br, I, CN, SO_3_H, and NO_2_). The *V*_s,max_ values of σ-holes for these compounds are shown in Table 1. For comparison, the magnitude of the σ-hole of the unsubstituted model (R = H) is +131 kcal/mol (Figures 4 and 5).

**Table 1:** Highest Positive Value of the Electrostatic Potential (*V*_s,max_) of σ-Holes Related to the Iodine Atom in the Substituted [(Pyridine)Iodine]^+^ Complexes, and Hammett Parameters of the Substituents^a^

	*V*_s,max_						
R	2-R-py	2,6-R_2_-py	3-R-py	3,5-R_2_-py	4-R-py	σ_p_	σ_m_
NMe_2_	123	114	123	117	118	–0.83	–0.21
NH_2_	128	126	125	122	121	–0.66	–0.16
OH	127	122	129	130	128	–0.37	0.12
OMe	125	118	128	125	125	–0.28	0.12
CH_3_	128	123	128	125	127	–0.17	–0.07
H	131	131	131	131	131	0	0
F	134	137	134	138	132	0.06	0.34
CH_2_F	130	130	130	130	129	0.11	0.12
Cl	131	130	132	133	131	0.23	0.37
Br	130	128	132	133	130	0.23	0.39
I	128	123	130	129	128	0.28	0.35
CHF_2_	130	131	132	133	132	0.32	0.29
CF_3_	133	134	136	138	135	0.54	0.43
SO_3_H	131	137	133	136	134	0.64	0.38
CN	135	138	137	144	136	0.66	0.56
NO_2_	136	142	139	144	138	0.78	0.71

a Energies are given in kcal/mol.

**Figure 6:**
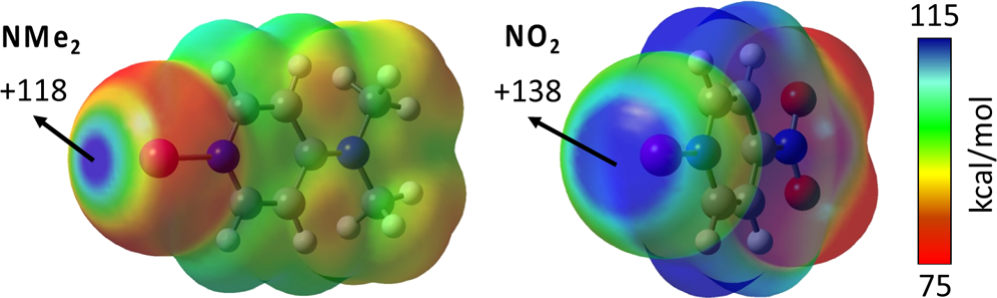
MEP maps of [{4-NMe_2_-py}I]^+^ and [{4-NO_2_-py}I]^+^ complexes calculated at the MN12-SX/def2-TZVP level and plotted on the electron density van der Waals isosurface (*s* = 0.001 Å). Energies are given in kcal/mol. Red and blue colors indicate less and more positive MEP values, respectively.

**Figure 7:**
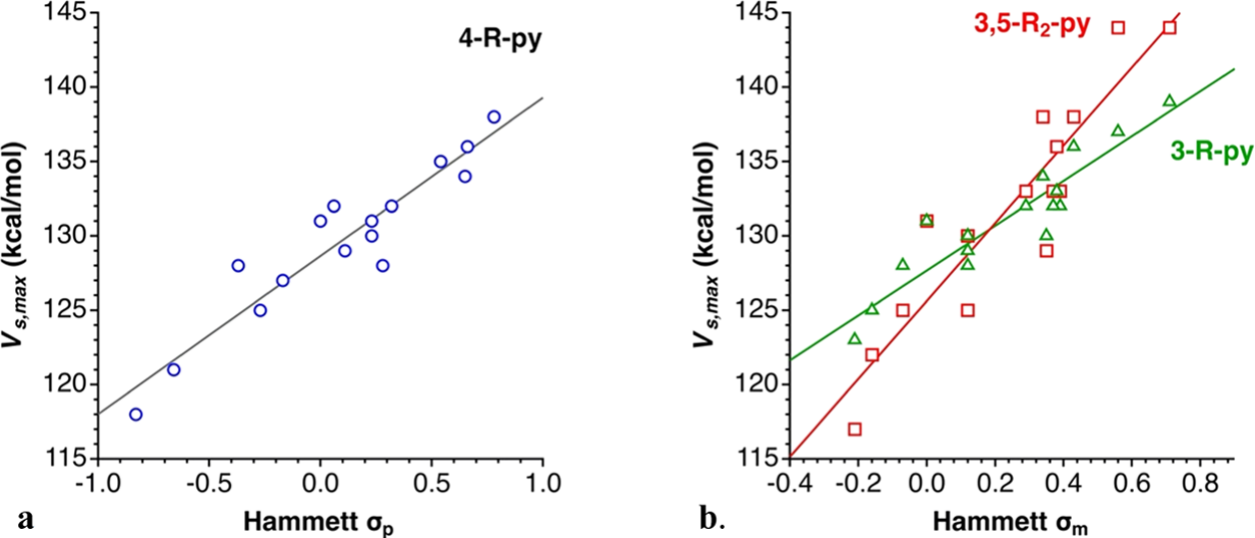
Dependence of the electrostatic potential at the σ-hole (*V*_s,max_) on the Hammett parameters for (a) the 4-R-py (*R*^2^ = 0.89) and (b) the 3-R-py (triangles, *R*^2^ = 0.86) and 3,5-R_2_-py (squares, *R*^2^ = 0.84).

The smallest positive MEP value is found for NMe_2_ and the highest ones for CN and NO_2_. Two examples of MEP maps are depicted in Figure 6 (for the MEP maps of all compounds studied, see Figure S3.1–5 in the Supporting Information). The *V*_s,max_ values of σ-holes show fair correlations with the electron-releasing power of the R groups measured by their Hammett σ_p_ (4-R-py) and σ_m_ (3-R-py and 3,5-R_2_-py) parameters, whereas no correlation is found for the *ortho* mono- and disubstituted pyridines (2-R-py and 2,6-R_2_-py), clearly indicating the important steric effects in these two cases (Figure 7 and eqs S1–S5 in the Supporting Information). Those trends are most clearly seen in the CH_3–*n*_F*_n_*-substituted pyridines, for which the magnitude of the σ-hole increases with the number of F atoms, and in the halogenated pyridines, for which it decreases on descending down the halogen group. The strongest inductive effect among singly substituted pyridines is found for the *para* derivatives. It must be noted also that the incorporation of two substituents at two equivalent positions of the pyridine ring practically doubles the effect of a single substituent. For 2,6-(SO_3_H)_2_-py, there are two additional σ-holes related to the O–H bond axis (both of 144 kcal/mol) parallel to the I···N σ-hole (see Supporting Information, Figure S3.2). Although the trends found in Figure 7 point to a clear influence of electron donor properties of the substituents on the electrostatic potential at the σ-hole, the correlation is somewhat poor, indicating that some fine-tuning is due to other effects.

In summary, it is expected that for a given Lewis base, and considering the electrostatic attraction as the main driving force, an increase of the *V*_s,max_ value will strengthen the interaction, whereas its reduction will weaken it.

## Analysis of the Interaction Energies

### Effect of the Central Atom X

The effect of varying the central atom on the bonding and geometry between the py and [X-py]^+^ fragments has been studied by means of DFT calculations. We have used the same X atoms as in the above MEP analysis (Figure 4, X = Cl, Br, and I). The main results are shown in Table 2. The optimized geometry of the bis-pyridine halonium(I) cations exhibits D_2h_ symmetry with two identical X···N bond distances. The calculated halogen···nitrogen bond lengths are 1.52 Å (N···Cl), 1.43 Å (N···Br), and 1.45 Å (N···I) shorter than the sum of the van der Waals radii of the involved atoms (3.48, 3.52, and 3.70 Å, respectively).^53^ The interaction energy increases in magnitude from Cl to I, in good agreement with the magnitude of the σ-holes.

**Table 2:** Key Geometrical Parameters and Interaction Energies for the Optimized [py···X···py]^+^ Model (X = Cl, Br, and I) Calculated at the MN12-SX/def2-TZVP Level

X	*d*_N–I_ (Å)	*p*_X···N_ (%)	α_N–I–N_ (°)	*V*_s,max_ (kcal/mol)	Δ*E*_int_ (kcal/mol)
Cl	1.961	86.8	180.0	114	–37.37
Br	2.088	88.5	180.0	120	–40.01
I	2.255	90.3	180.0	131	–41.30

Notice that the interpenetration of the van der Waals cores increases slightly with the atomic size of X and that the values of around 87−90% are consistent with those of other hypercoordinated compounds.^54^ While the maximum electrostatic potential at the σ-hole decreases linearly with the Pauling electronegativity of the halogen, the interaction energy shows a linear dependence on the penetration indices.

### Effect of the Donor

The effect of different N-donor groups (D) on the bond strength and geometry of the D**···**[I–D]^+^ interaction (Scheme 2) has been analyzed using the same donors as in the MEP analysis (Figure 5), and the results are shown in Table 3. The optimized [N···I···N]^+^ backbones’ are all linear with two identical I···N distances. Ammonia, which induces the most marked σ-hole (151 kcal/mol), has an interaction energy of −43.48 kcal/mol. However, even if imidazole has the lowest *V*_s,max_ value (130 kcal/mol), it gives the most stable adduct. Furthermore, the acetonitrile iodonium is less stable than the pyridine one even though the *V*_s,max_ value of the former is as high as 150 kcal/mol. In addition, pyrimidine yields the least stable adduct although its *V*_s,max_ value is the highest among the aromatic donors (135 kcal/mol). It seems thus clear that the strength of the bond cannot be explained by Coulombic interactions only.

**Table 3:** Key Geometrical Parameters and Interaction Energies for the Optimized [D···I···D]^+^ Models with Different N-Donor Lewis Bases (D) Calculated at the MN12-SX/def2-TZVP Level

donor	*d*_N–I_ (Å)	*p*_I···N_ (%)	α_N–I–N_ (°)	*V*_s,max_ (kcal/mol)	Δ*E*_int_ (kcal/mol)
pyrimidine	2.261	89.9	179.8	135	–38.50
acetonitrile	2.190	94.4	180.0	150	–40.09
pyridine	2.255	90.3	180.0	131	–41.30
ammonia	2.299	87.6	180.0	151	–43.48
imidazole	2.238	91.4	180.0	130	–44.87

Among the aromatic donors, the interaction energy increases with the penetration index, while for the differently hybridized N-donor atoms, the interaction energy seems to follow the following trend: sp^3^ > sp^2^ > sp, considering the average value for the three aromatic ligands, 25(2) kcal/mol.

### Effect of the R Group

Upon substitutions at the *ortho* (2-R-py), double *ortho* (2,6-R_2_-py), *meta* (3-R-py), double *meta* (3,5-R_2_-py), and *para* (4-R-py) positions, relative to the pyridine nitrogen of the [bis(pyridine)iodine]^+^ adducts, with the same substituents used in our previous MEP analysis (Table 1), we have analyzed the impact of the electron density alteration on the stability and geometry of the bonding between pyridine and [I-py]^+^ (Scheme 2). Previous works using the same compound have only focused on substitution of the pyridine *para*-hydrogen using a few substituents.^17,46^ The numerical results are summarized in Tables S3.1–5 in the Supporting Information.

**Figure 8:**
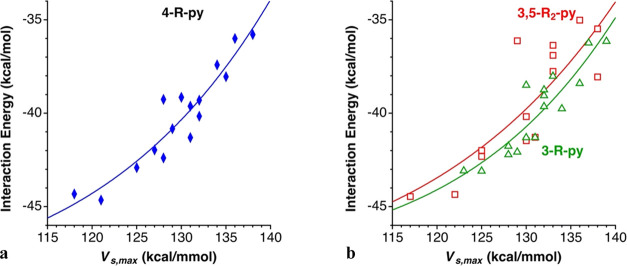
Interaction energy between a pyridine donor (D) and the corresponding [I–D]^+^ cation, represented as a function of the electrostatic potential at the σ-hole (*V*_s,max_) for (**a**) the 4-R-py (*R*^2^ = 0.89) and (**b**) the 3-R-py (triangles, *R*^2^ = 0.82) and 3,5-R_2_-py (squares, *R*^2^ = 0.84) adducts.

When comparing the interaction energy between a pyridine donor (D) and the corresponding [I–D]^+^ cation with the electrostatic potential at the σ-hole (*V*_s,max_), some correlation between the two parameters are found for the 4-R-py, 3-R-py and 3,5-R_2_-py families (Figure 8 and eqs S6–S8 in the Supporting Information), whereas no correlation is found for the *ortho*-substituted 2-R-py and 2,6-R_2_-py families. The surprising aspect of that correlation is that it is positive, i.e., the interaction energy is made less attractive as the electrostatic potential increases. These results clearly indicate that the attractive interaction between the two moieties is modulated via substituents by forces other than the electrostatic attraction associated with the σ-hole at the [I–D]^+^ cation. Consider, for instance, the 2,6-(NO_2_)_2_-py iodonium cation that appears in the calculations as the least stable one (Δ*E*_int_ = −30.56 kcal/mol), yet the electrostatic potential of the [I–D]^+^ cation at its σ-hole is among the highest ones (142 kcal/mol). Another clear example is the 4-NH_2_-py adduct, calculated to be the most stable one (Δ*E*_int_ = −44.65 kcal/mol) despite the rather low *V*_s,max_ value (121 kcal/mol) of the interacting iodonium group.

**Figure 9:**
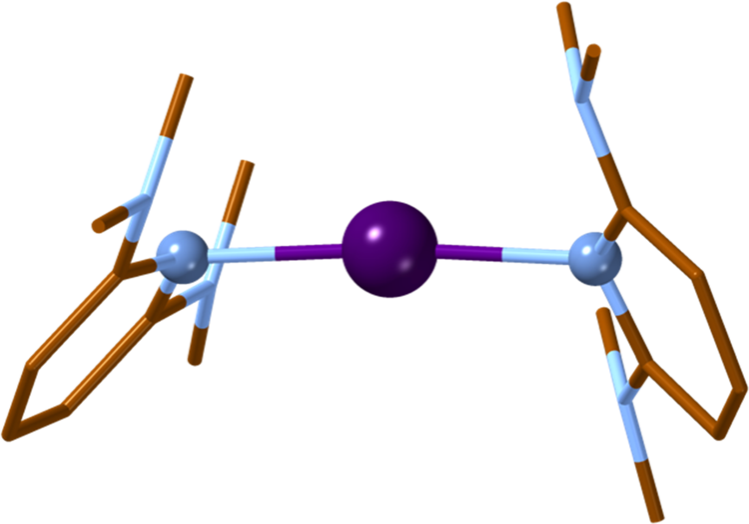
Optimized geometry for the [{2,6-(NMe_2_)_2_-py}_2_I]^+^ complex. Color code: brown, C; blue, N; and purple, I. H atoms are omitted for clarity.

**Figure 10:**
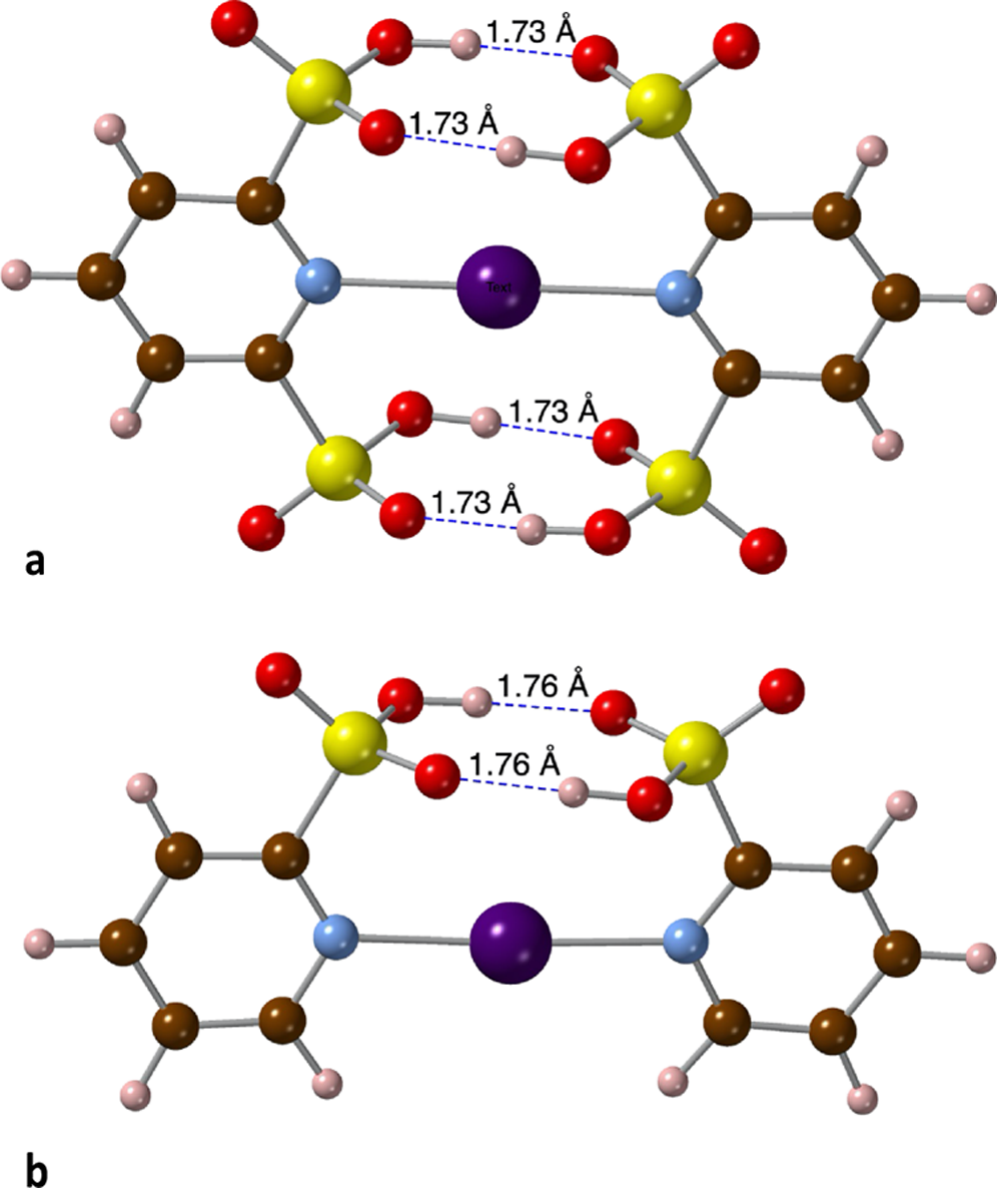
Hydrogen bonds (dashed lines) in the calculated structure of (a) [{2,6-(SO_3_H)_2_-py}_2_I]^+^ and (b) [*cis*-{2-(SO_3_H)-py}_2_I]^+^ adducts.

All optimized compounds show linear [N···I···N]^+^ frameworks with two equal I···N distances, although a slight bending of a few degrees is obtained for singly *ortho*-substituted pyridines and for the [{2,6-(NMe_2_)_2_-py}_2_I]^+^ cation (Tables S3.1,2, respectively, in the Supporting Information). It must be stressed that, at difference with the wide range of interaction energies induced by the nature and positions of the substituents at the pyridine ring, the I···N distances fall in a narrow range (2.24–2.27 Å) for the *meta* and *para*-substituted pyridines, while they result more variable and longer (2.26–2.36 Å) for the *ortho*-substituted ones. For those *ortho*-substituted compounds, the steric hindrance and other secondary interactions such as hydrogen bonds surely play an important role in the geometry and stability of the system. For instance, despite the strong electron-releasing character of NMe_2_, the I···N distance in the 2,6-NMe_2_-py compound is longer, and the interaction energy smaller, than in the unsubstituted compound. It is worth mentioning here that the influence of hydrogen bonds on the structure of the related [Cl_3_]^−^ system has been previously reported.^55^ In addition, the pyridine donors form a handle-shaped structure around the central [N···I···N]^+^ framework (Figure 9), probably due to the steric hindrance of the methyl groups. Furthermore, in the 2,6-(SO_3_H)_2_-py adduct, four O···H hydrogen bonds of 1.73 Å are formed between the hydrogen sulfonato groups (Figure 10a), explaining the unusually high interaction energy between the two fragments (Δ*E*_int_ = −60.67 kcal/mol), to be compared with the much smaller value for the monosubstituted *trans*-derivative, 2-SO_3_H-py (−38.73 kcal/mol). Comparison with the interaction energy of the monosubstituted *cis*-derivative 2-SO_3_H-py (−49.86 kcal/mol), which shows two O···H hydrogen bonds of 1.76 Å formed between the substituents (Figure 10b), allows us to estimate the stabilization energy of each hydrogen bond in 5.41 kcal/mol. The interaction energies between *ortho*-substituted pyridines, 2-R-py and 2,6-R_2_-py, and the corresponding [I–D]^+^ cations are affected by the same effects. As a result, neither the I···N distances nor the interaction energies in this family correlate with the electron-withdrawing/releasing power of the substituents, calibrated by the Hammett σ_p_ parameters. Notice that the same effects have prevented the definition of a Hammett parameter for substituents in the *ortho* position. Also note that although the interatomic distance might correlate with the interaction energy in some cases, the bond length is not necessarily an appropriate general measure of the strength of a bond.^56^

**Figure 11:**
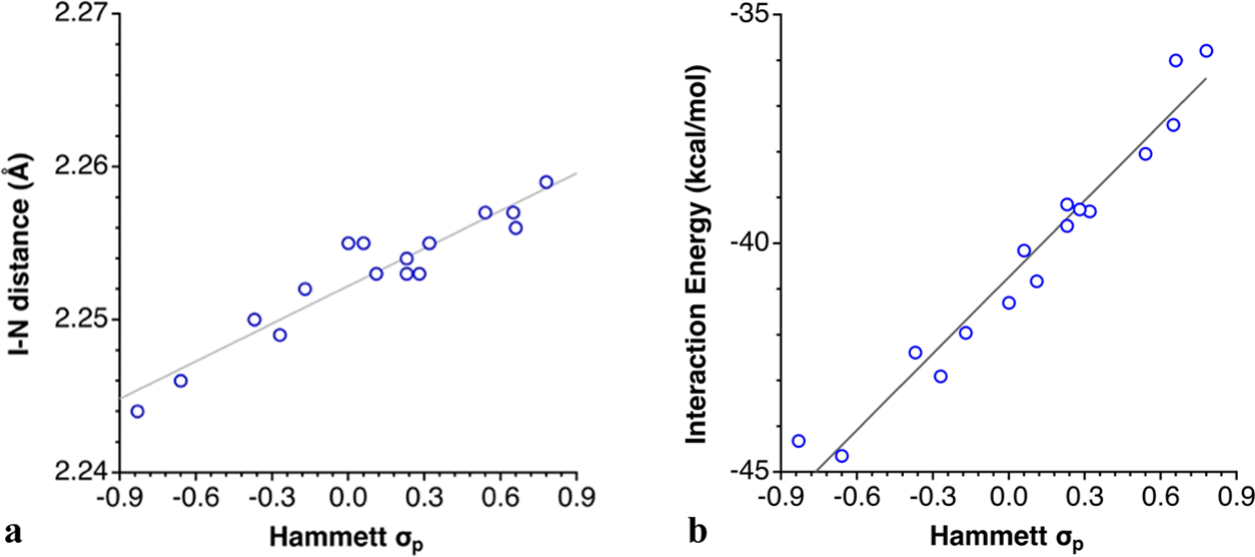
Dependence on the Hammett σ_p_ parameter of (a) the I–N bond distances (*R*^2^ = 0.90) and (b) the interaction energy between the N and [I–N]^+^ fragments (*R*^2^ = 0.96) in 4-R-py compounds.

**Figure 12:**
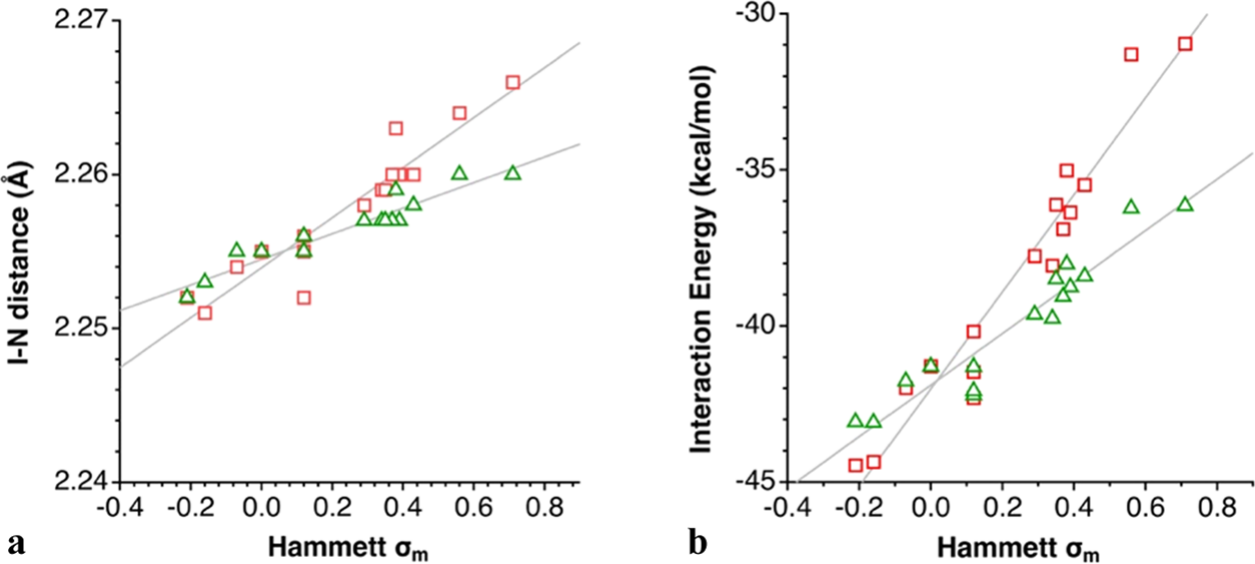
Dependence on the Hammett σ_m_ parameter of (a) the I–N bond distances (*R*^2^ = 0.91, 0.89) and (b) the interaction energy between the N and [I–N]^+^ fragments (*R*^2^ = 0.91, 0.94) in 3-R-py (triangles) and 3,5-R_2_-py (squares) compounds.

Both the I···N distances and the interaction energies of 3-R-py, 3,5-R_2_-py, and 4-R-py compounds are expected to remain unaffected by steric hindrance and other intramolecular secondary interactions between the R groups and thus are most adequate to analyze the effect of changes in the electron density of the Lewis base. Plots of those two parameters as a function of the Hammett σ_p_ and σ_m_ constants of the pyridine substituents (Figures 11 and 12, respectively) show that they are nicely correlated (see eqs S9–S10 and S11–S14, respectively, in the Supporting Information). Such behaviors bear some similarities with the trends just discussed for the electrostatic potential at the σ-hole. Now the highest interaction energies within each substitution scheme are found for R = NH_2_ and NMe_2_ and the lowest ones for CN and NO_2_. As for the general trends, the I···N distance increases, and the interaction energy decreases, as the Hammett parameters become more positive, i.e., as the electron-releasing power decreases or the electron-withdrawing ability increases. The effect of a *para* substitution at the pyridine ring on the interaction energy is stronger than a single *meta-*substitution. However, the incorporation of a second substituent at the *meta*-position practically doubles the effect of a single substituent, as can be appreciated by the higher slope of the least-squares lines for the 3,5-R_2_-py compounds with respect to the 3-R-py analogues (Figures 12a,b and eqs S11–S14 in the Supporting Information).

Those trends can be clearly seen in the subsets of CH_3–*n*_F*_n_* and halogen-substituted pyridines (Table S3.1–5). For the former, the interaction energy is made less stabilizing as the number of F atoms increase, within every family with a given substitution pattern. While single substitution with these groups induces changes in the interaction energy of up to 3.4 kcal/mol, double substitution modifies it by 6.5 kcal/mol. Among the halogen-substituted pyridines, the interaction energy decreases on descending down the halogens group, but with smaller changes than those induced by the fluoromethyl groups.

The fact that both the I–N distance and the interaction energy show a dependence on the Hammett parameter of the substituent means that there is also some correlation between the distance and the interaction energy, and comparison of bond distances in halonium ions should give approximate information on relative interaction energies. Moreover, the electrostatic potentials at the σ-hole (*V*_s,max_) are also correlated with the Hammett parameters, as can be seen in Figure 7a,b and, consequently, the interaction energy becomes less attractive as the electrostatic potential at the σ-hole increases, clearly showing that such an interaction is not the main responsible for the strength of the I–N bonds in the studied [D···I···D]^+^ iodonium cations.

## Energy Decomposition Analysis

In light of the above results, we have performed an energy decomposition analysis (EDA) to investigate the effect of (i) the central atom X, (ii) the N-donor D, and (iii) the substituents R on the nature of the bond between D and [X–D]^+^. EDA schemes are a useful tool to understand the physical origin of a given interaction that have attracted increasing interest in recent years among theoretical chemists.^57−61^

### Effect of Central Atom X

The results for three halogen atoms (X = Cl, Br, and I) with D = pyridine are summarized in Table 4. The orbital-based interactions, shown as polarization (Δ*E*_pol_) and charge transfer (Δ*E*_ct_) terms, decrease as we go down the group of the halogens. The less electronegative the central atom is, the higher the energy of its np orbitals, thus enlarging the energy gap between the donor and acceptor orbitals and allowing for a poorer orbital interaction. Simultaneously, the electrostatic (Δ*E*_elec_) contribution increases, accordingly with the polarizability of the central atom. The sum of Δ*E*_pol_ and Δ*E*_ct_ represents 51% (Cl), 47% (Br), and 44% (I) of the total attractive interaction energy. For the three halogens, the electrostatic term is not enough to overcome the Pauli repulsion but the significant contributions of polarization, charge transfer and, to a lesser extent (<5%) dispersion make the net interaction attractive (see Figure S4 in the Supporting information).

**Table 4:** EDA for the Interaction of Pyridine and [X-py]^+^ in the Optimized [py···X···py]^+^ Model (X = Cl, Br, and I), Calculated at the MN12-SX/def2-TZVP Level^a^

X	Δ*E*_Pauli_	Δ*E*_elec_	Δ*E*_disp_	Δ*E*_pol_	Δ*E*_ct_	Δ*E*_int_
Cl	145.88	–81.41 (44.4%)	–8.83 (4.8%)	–25.08 (13.7%)	–67.93 (37.1%)	–37.37
Br	125.62	–79.71 (48.1%)	–8.03 (4.8%)	–26.86 (16.2%)	–51.14 (30.9%)	–40.12
I	105.56	–74.44 (50.7%)	–7.16 (4.9%)	–25.94 (17.7%)	–39.32 (26.8%)	–41.29

a The percentage represents the contribution to the sum of the attractive (negative) contributions to the interaction energy. Energies are given in kcal/mol.

### Effect of the Donor

The EDA results for the interaction between various N-donors D and the corresponding [I–D]^+^ cation (Scheme 2) are summarized in Table 5. The first observation is that the electrostatic term is the largest attractive contribution, yet it is insufficient to overcome the Pauli repulsion. The dispersion term contributes less than 5%, whereas the polarization and charge transfer terms contribute nearly as much (39–45%) as the electrostatic term to the attractive part of the interaction, making the formation of the adduct energetically favorable in all cases by 38–45 kcal/mol. Among the aromatic donors, imidazole has the greatest magnitude of Δ*E*_INT_, whereas pyrimidine has the smallest one, while the proportion of orbital contribution to the total attractive interactions is smallest for imidazole (43%) and largest for pyrimidine (45%). For the nonaromatic donors, the ammonia iodonium interacts more strongly than the acetonitrile one. All in all, these results indicate that, in all cases, the electrostatic term is not enough to overcome the Pauli repulsion and it is the combined effect of the orbital-based terms (polarization and charge transfer) and a smaller contribution of dispersion forces, which makes the interaction attractive (see Figure S5 in the Supporting information).

**Table 5:** EDA for the Interaction of Different Nitrogen-Containing Lewis Bases (D) and the Corresponding [I–D]^+^ in the Optimized [D···I···D]^+^ Models, Calculated at the MN12-SX/def2-TZVP Level^a^

donor	Δ*E*_Pauli_	Δ*E*_elec_	Δ*E*_disp_	Δ*E*_pol_	Δ*E*_ct_	Δ*E*_int_
pyrimidine	101.84	–70.02 (49.9%)	–6.71 (4.8%)	–25.44 (18.1%)	–38.17 (27.2%)	–38.50
acetonitrile	91.91	–67.33 (51.0%)	–5.53 (4.2%)	–23.28 (17.6%)	–35.86 (27.2%)	–40.09
pyridine	105.56	–74.44 (50.7%)	–7.16 (4.9%)	–25.94 (17.7%)	–39.32 (26.8%)	–41.29
ammonia	98.80	–81.42 (57.2%)	–5.53 (3.9%)	–18.06 (12.7%)	–37.27 (26.2%)	–43.49
imidazole	107.68	–80.62 (52.9%)	–6.96 (4.6%)	–26.30 (17.2%)	–38.66 (25.3%)	–44.86

a The percentage represents the contribution to the sum of the attractive (negative) contributions to the interaction energy. Energies are given in kcal/mol.

### Effect of the R Group

Finally, we have applied the same approach to analyze how the energetic contributions change with the electron density distribution of the [N···I···N]^+^ halonium bond. The modifications of the electron density were achieved by modulation of *ortho* (2-R-py and 2,6-R_2_-py), *meta* (3-R-py and 3,5-R_2_-py), and *para*-position (4-R-py) relative to the pyridine nitrogen of the [py···I···py]^+^ complex (R = H, Scheme 2), with the same R groups as in the previous sections. The numerical results are summarized in Tables S4.1–5 in the Supporting Information.

As happens with the interaction energies, the electrostatic, dispersion, and orbital terms remain unaffected by the steric hindrance and other intramolecular secondary interactions between the R groups for 3-R-py, 3,5-R_2_-py, and 4-R-py compounds, and they can therefore be used to study the nature of the [N···I···N]^+^ halonium bonding. In contrast, extreme caution must be taken when interpreting the results obtained for 2-R-py and 2,6-R_2_-py compounds. Although these complexes somewhat follow the same trend previously found, the EDA analysis not only decomposes the energetic contributions between the I···N bond, but it is sensible to all the interactions among the fragments, including secondary interactions between the R groups.

**Figure 13:**
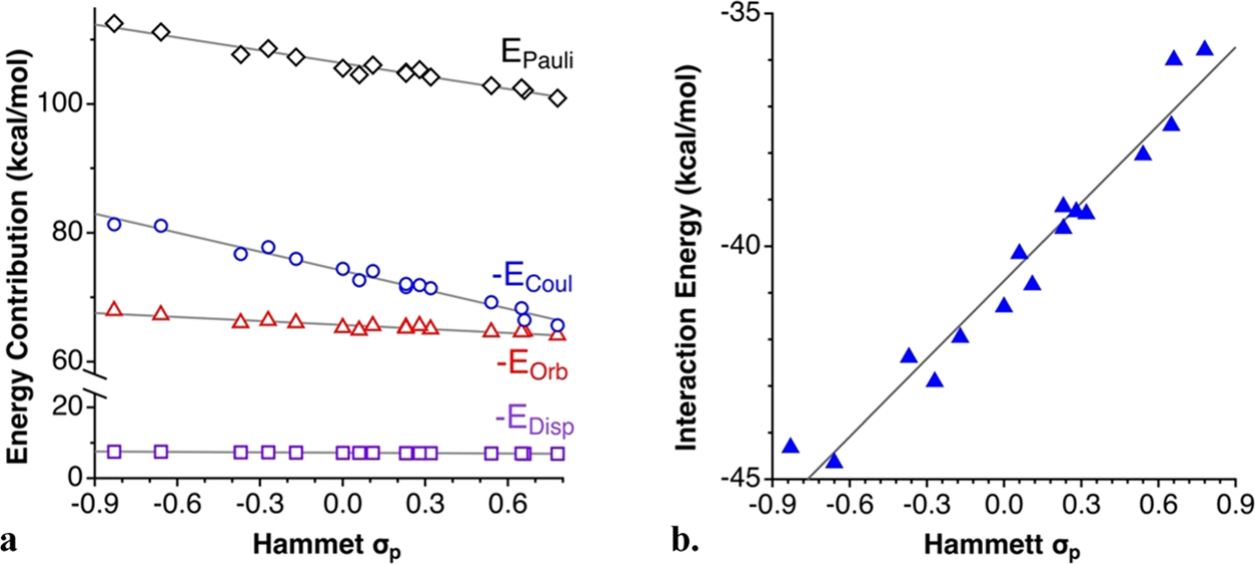
Dependence on the Hammett σ_p_ parameter of (a) the EDA contributions to the interaction energy and (b) the net interaction energy between the 4-R-py donors and the [(4-R-py)-I]^+^ cations. Δ*E*_Pauli_, Δ*E*_Coul_, Δ*E*_orb_, and Δ*E*_disp_ are the Pauli repulsion, electrostatic, orbital (Δ*E*_orb_ = Δ*E*_pol_ + Δ*E*_ct_), and dispersion, contributions to the interaction energy, respectively.

For those substitution patterns affecting the *meta* or *para*-positions, for which it is sensible to employ Hammett parameters, the interaction energy, as well as the four contributions obtained from the EDA analysis present a clear dependence on the electron-releasing power of the substituents. This correlation can be seen for the 4-R-py compounds in Figure 13a, and linear least-squares fittings are described by eqs 1–5 (all energies in kcal/mol), where Δ*E*_orb_, Δ*E*_Coul_, Δ*E*_disp_, and Δ*E*_Pauli_ are the sum of the polarization and charge transfer (Δ*E*_pol_ + Δ*E*_ct_), electrostatic, dispersion, and Pauli repulsion contributions to the interaction energy (Δ*E*_int_), respectively. Although for highly electron-withdrawing substituents (positive σ_p_ values) the weights of the electrostatic and orbital terms are similar, the former presents a stronger dependence on the Hammett parameter and becomes predominant for highly electron-releasing groups (negative σ_p_ values). In all cases, however, the orbital interactions are significant and necessary to overcome the Pauli repulsion.

Notice that although the Pauli repulsion increases with decreasing σ_p_, the slope of the electrostatic term is larger, and the net interaction energy is consequently more attractive for negative Hammett parameters (Figure 13a).
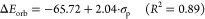
1
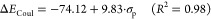
2
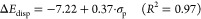
3
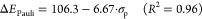
4
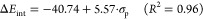
5

**Figure 14:**
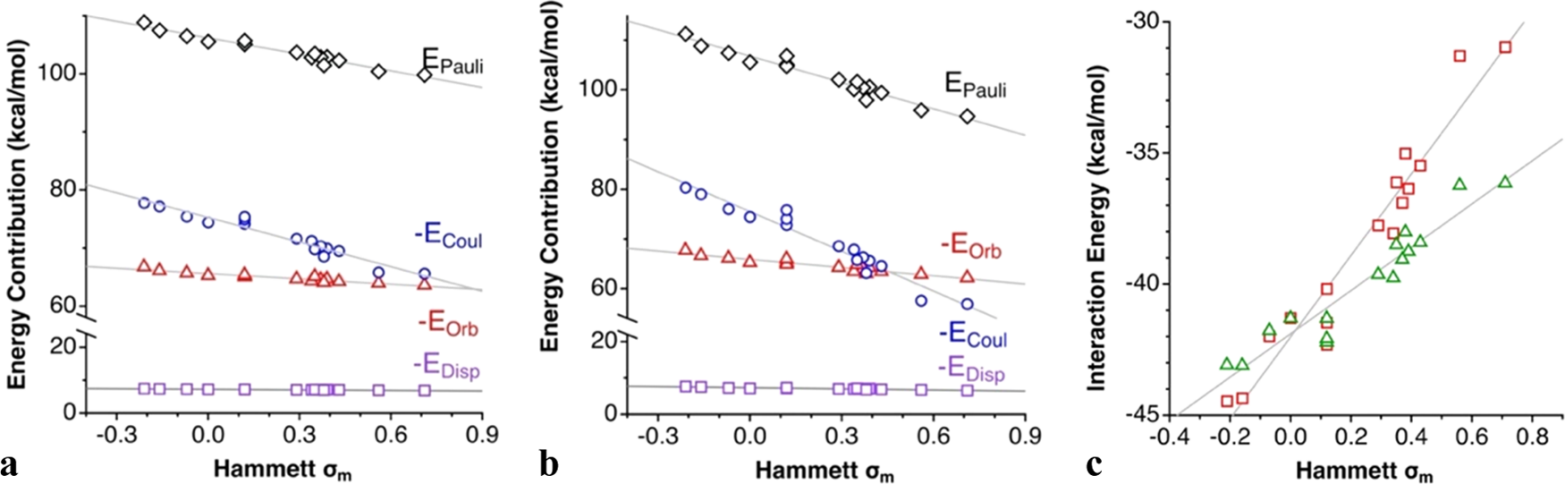
Dependence on the Hammett σ_m_ parameters of (a) the EDA contributions to the interaction energy between the 3-R-py donors and the [(3-R-py)-I]^+^ cations, (b) similarly for (3,5-R_2_-py) analogues, and (c) the net interaction energy for the two families of compounds (triangles for monosubstituted, squares for disubstituted pyridine).

Similar considerations can be made for 3-R-py adducts (Figure 14a and eqs 6–10)
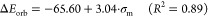
6
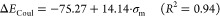
7
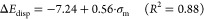
8
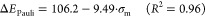
9
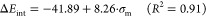
10as well as for 3,5-R_2_-py adducts (Figure 14b and eqs 11–15). The main difference with the 4-R derivatives appears for the disubstituted adducts that induce much wider variations of the interaction energies with the σ_m_ value (14 kcal/mol) than the monosubstituted ones (7 kcal/mol).
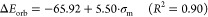
11
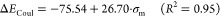
12
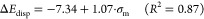
13
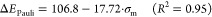
14
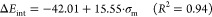
15

In the *meta* and *para* substitution patterns, the contribution of dispersion forces to the total attractive interaction is small, in good agreement with the results of previous works,^33^ and shows little dependence on the Hammett parameters. In contrast, the electrostatic and Pauli contributions are strongly dependent on the nature of the substituent. It is important to stress that for each of the sets of systems studied (Tables S4.1–5 in the Supporting Information) there is a fair linear correlation between the Pauli and electrostatic contributions (see Figure S6 in the Supporting Information). The sum of these two terms is in all cases positive, with values between 25.3 and 48.0 kcal/mol, and an average value of 33(2) kcal/mol. Since the orbital contribution has in all cases negative values in excess of 50 kcal/mol, it is clear that they overcome the combined effect of the electrostatic and Pauli terms. Therefore, the orbital interaction, schematically represented by the MO diagram of Figure 1, must be blamed responsible for the attractive nature of the interaction, which is enhanced by highly electron-withdrawing groups.

## Halonium Bonding in Trihalide Anions

**Figure 15:**
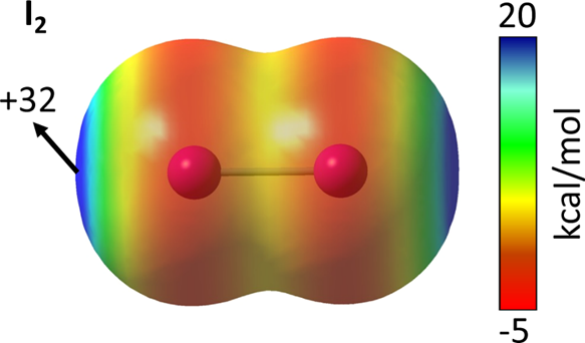
MEP map of the I_2_ molecule calculated at the MN12-SX/def2-TZVP level and plotted on the electron density van der Waals isosurface (*s* = 0.001 Å). Energies are given in kcal/mol. Red and blue indicate more negative and more positive MEP values, respectively.

As mentioned in the Introduction Section, trihalide anions behave very similarly to halonium cations. For example, the bonding in I_3_^–^ could be described as the interaction between two closed-shell groups, I_2_ and I^–^, and rationalized by the σ-hole model. The anisotropic distribution of the electron density of I_2_ forms two opposite σ-holes (both of 32 kcal/mol, Figure 15) at each side of the I–I bond axis capable of accepting an electron pair of I^–^ at either side of the iodine molecule.

The optimized geometry of I_3_^–^ is linear (179.9°) and exhibits a *D*_∞*h*_ symmetry with two identical I–I bond distances (2.959 Å), 1.12 Å shorter than the sum of the van der Waals radii of the involved atoms (4.08 Å), or an 86% penetration of the van der Waals crusts, compared to 110% in the I_2_ molecule and consistent with the values found in other hypercoordinated systems.^54^ Despite the low *V*_s,max_ value of its σ-holes (Figure 15), the interaction energy of the complex is as high as −39.18 kcal/mol, comparable in strength to those in the halonium cations discussed above. An EDA analysis shows that the electrostatic and orbital contributions (especially charge transfer) have similar weight and, with a small contribution of the dispersion term (<5%), both are necessary to overcome the Pauli repulsion and make thus the net interaction energy attractive (Table 6). To sum up, the nature of the interactions that governs the bonding in halonium ions can also explain the geometric parameters and the bonding in trihalides anions.

**Table 6:** EDA for the Interaction of I^–^ Anion and I_2_ Molecule in the Optimized I_3_^–^ Anion, Calculated at the MN12-SX/def2-TZVP Level^a^

X_3_^–^	Δ*E*_Pauli_	Δ*E*_elec_	Δ*E*_disp_	Δ*E*_pol_	Δ*E*_ct_	Δ*E*_int_
I_3_^–^	81.85	–53.79 (44.4%)	–5.96 (4.9%)	–15.14 (12.5%)	–46.13 (38.1%)	–39.18

a The percentage represents the contribution to the sum of the attractive (negative) contributions to the interaction energy. Energies are given in kcal/mol.

## Conclusions

We have carried out a combined structural and computational analysis of the bonding in [N···I···N]^+^ halonium groups. Only linear [N···I···N] frameworks with neutral donors have been found in the CSD, with nearly equal I···N distances and angles in the range of 175–178°. An MEP analysis has disclosed a well-defined σ-hole at the halogen atom for all compounds studied. Such electron depletion is consistent with the geometric preferences of the interaction since the bond with the Lewis base along the X···N axis (X = Cl, Br, and I) is favored by Coulombic attraction. The value of the electrostatic potential at the σ-hole increases on going down the halogen group, in good agreement with the polarizability of the central atom, and its value can be modulated by the donor group D and the nature of its substituent R.

The strength of the interaction depends on the nature of both the donor and the acceptor. The stability of the adduct increases with the size of the halogen, in good agreement with the MEP analysis. However, aromatic donors show high interaction energies even though the magnitude of their σ-hole is low compared to ammonia and acetonitrile donors. Electron density changes have a strong influence on the stability of the [N···I···N]^+^ halonium bond in 3-R-py, 3,5-R_2_-py, and 4-R-py complexes, whereas the I···N bond length remains virtually unaltered. The correlation of the Hammett σ_m_ and σ_p_ constants, respectively, with the I···N distances, the interaction energy, and the *V*_s,max_ indicates that the [N···I···N]^+^ halonium bond is made less stable as the electrostatic potential at the σ-hole and the I···N distances increase. The steric hindrance and other secondary interactions between the R groups in 2-R-py and 2,6-R_2_-py complexes play an important role in the stability of the complex. Consequently, the lack of correlation between the Hammett σ_p_ parameters and the three parameters mentioned above prevents us from discussing further along this line.

Our energy decomposition analysis (EDA) results shed light on the physical nature of the interaction. As descending down the halogen group, the orbital-to-dispersion ratio decreases (Cl = 10.53:1, Br = 9.71:1, and I = 9.11:1), in good agreement with the atomic volume of the central atom X. Regarding the effect of the donors (D), the aromatic groups present the lowest orbital to dispersion ratio (pyrimidine = 9.48:1, pyridine = 9.11:1, and imidazole = 9.33:1). Among the nonaromatic ones, acetonitrile (10.69:1) is a better donor than ammonia (10.01:1). As in the interaction energy, the four energetic contributions obtained from the EDA results of 3-R-py, 3,5-R_2_-py, and 4-R-py adducts correlate very well with the Hammett σ_m_ and σ_p_ parameters, respectively. The Coulombic and Pauli repulsion terms are linearly correlated within each family and are overall repulsive. As in the systems previously studied in our group,^62,63^ the orbital and dispersion contributions are required to overcome the net repulsion of the electrostatic and Pauli terms, but in this case, the orbital terms are much stronger than the dispersion contribution. In the compounds with distal substituents (3, 4, and 5 positions) the orbital/dispersion ratio is nearly invariably 9:1, whereas *ortho* mono- and disubstitutions reduce that proportion to 7.3:1 and 5.7:1, respectively. To sum up, it seems clear that a pure electrostatic model of the σ-hole or “halogen bond” is inadequate to explain the stability of the halonium ions and the Pimentel–Rundle delocalized molecular orbital picture (Figure 1) is more adequate. Since trihalide anions, X_3_^–^, behave very similarly to halonium cations, the same conclusions found for the halonium ions can be applied to explain the geometric parameters and the nature of the bonding in those anions, as substantiated by present calculations on the triiodide anion.

We believe that these findings allow for a better understanding of the nature and factors that govern the bonding and the geometry of three-center-four-electron halonium compounds, and open new ways to enable novel applications as halonium transfer agents, asymmetric halofunctionalization, or as building blocks in supramolecular chemistry, and, ultimately, contribute to our understanding of chemical bonding at large.

## Computational Methods

Structural searches were carried out in the Cambridge Structural Database (CSD)^49^ version 5.42, November 2020. Only crystal structures with three-dimensional (3D) coordinates determined, nondisordered, with no errors, not polymeric, and with *R* < 0.1 were allowed in searches. CSD refcodes of selected examples are given throughout the text as six-letter codes (e.g., ABCDEF). We used the van der Waals radii proposed by Alvarez.^53^ DFT calculations were performed using the Gaussian 16 package^64^ with the MN12-SX functional and the def2-TZVP basis set for all atoms, with the corresponding relativistic pseudopotentials for heavy atoms. The functional was chosen after an extensive benchmark of the performance of 11 functionals (with and without Grimme’s D3^65^ and D3BJ^66^ dispersion correction) and the MP2 method for the calculation of bond distance, angles, and the interaction energy in bis(acetonitrile)-iodonium cation (BUKNAX).^47^ The results were compared with the experimental and calculated data at the CCSD and CCSD(T) levels. All structures were fully optimized and confirmed to be minima of the corresponding potential energy surfaces by frequency calculations. Interactions energies were calculated via the supermolecule approach and corrected for the BSSE by means of the counterpoise method.^67^ Only heterolytic dissociation of halonium compounds was taken into account by considering D and [X–D]^+^ as the interacting fragments. MEP maps were built on the 0.001 Å isosurface with GaussView 6 program^68^ on the molecular geometries of the interacting systems. EDA analyses were carried out with Q-Chem 5.3 software^69^ by means of the second-generation ALMO-EDA method.^70^

## Supporting Information Available

Cartesian coordinates of all optimized systems (*xyz*); benchmarking studies for the [N···I···N]^+^ halonium system; analysis of the molecular electrostatic potential; analysis of the interaction energies; and complete results of the energy decomposition analysis and structural searches (PDF)

## References

[ref1] Desiraju G. R.; Ho P. S.; Kloo L.; Legon A. C.; Marquardt R.; Metrangolo P.; Politzer P.; Resnati G.; Rissanen K. Definition of the halogen bond (IUPAC Recommendations 2013). Pure Appl. Chem. 2013, 85, 1711–1713. 10.1351/PAC-REC-12-05-10.

[ref2] Metrangolo P.; Neukirch H.; Pilati T.; Resnati G. Halogen Bonding Based Recognition Processes: A World Parallel to Hydrogen Bonding. Acc. Chem. Res. 2005, 38, 386–395. 10.1021/ar0400995. 15895976

[ref3] Metrangolo P.; Resnati G. Halogen versus Hydrogen. Science 2008, 321, 918–919. 10.1126/science.1162215. 18703728

[ref4] Umeyama H.; Morokuma K. The Origin of Hydrogen Bonding. An Energy Decomposition Study. J. Am. Chem. Soc. 1977, 99, 1316–1332. 10.1021/ja00447a007.

[ref5] Grabowski S. J. Hydrogen and halogen bonds are ruled by the same mechanisms. Phys. Chem. Chem. Phys. 2013, 15, 7249–7259. 10.1039/c3cp50537e. 23576180

[ref6] Syzgantseva O. A.; Tognetti V.; Joubert L. On the Physical Nature of Halogen Bonds: A QTAIM Study. J. Phys. Chem. A 2013, 117, 8969–8980. 10.1021/jp4059774. 24000903

[ref7] Wolters L. P.; Bickelhaupt F. M. Halogen Bonding versus Hydrogen Bonding: A Molecular Orbital Perspective. ChemistryOpen 2012, 1, 96–105. 10.1002/open.201100015. 24551497 PMC3922460

[ref8] Corradi E.; Meille S. V.; Messina M. T.; Metrangolo P.; Resnati G. Halogen Bonding versus Hydrogen Bonding in Driving Self-Assembly Processes. Angew. Chem., Int. Ed. 2000, 39, 1782–1786. 10.1002/(SICI)1521-3773(20000515)39:10<1782::AID-ANIE1782>3.0.CO;2-5. 10934360

[ref9] Aakeröy C. B.; Desper J.; Helfrich B. A.; Metrangolo P.; Pilati T.; Resnati G.; Stevenazzi A. Combining halogen bonds and hydrogen bonds in the modular assembly of heteromeric infinite 1-D chains. Chem. Commun. 2007, 4236–4238. 10.1039/b707458a. 18217591

[ref10] Saha B. K.; Nangia A.; Jaskólski M. Crystal engineering with hydrogen bonds and halogen bonds. CrystEngComm 2005, 7, 355–358. 10.1039/b501693b.

[ref11] Velasquez J. D.; Mahmoudi G.; Zangrando E.; Gurbanov A. V.; Zubkov F. I.; Zorlu Y.; Masoudiasl A.; Echeverría J. Experimental and theoretical study of Pb···S and Pb···O *σ*-hole interactions in the crystal structures of Pb(II) complexes. CrystEngComm 2019, 21, 6018–6025. 10.1039/C9CE00959K.

[ref12] Scheiner S. Coordination of a Central Atom by Multiple Intramolecular Pnicogen Bonds. Inorg. Chem. 2020, 59, 9315–9324. 10.1021/acs.inorgchem.0c01177. 32496762

[ref13] Zierkiewicz W.; Wysokiński R.; Michalczyk M.; Scheiner S. Chalcogen bonding of two ligands to hypervalent YF_4_ (Y = S, Se, Te, Po). Phys. Chem. Chem. Phys. 2019, 21, 20829–20839. 10.1039/C9CP04006D. 31517347

[ref14] Politzer P.; Murray J. S.; Clark T. Halogen bonding: an electrostatically-driven highly directional noncovalent interaction. Phys. Chem. Chem. Phys. 2010, 12, 7748–7757. 10.1039/c004189k. 20571692

[ref15] Politzer P.; Murray J. S.; Clark T. Halogen bonding and other *σ*-hole interactions: a perspective. Phys. Chem. Chem. Phys. 2013, 15, 11178–11189. 10.1039/c3cp00054k. 23450152

[ref16] Lommerse J. P. M.; Stone A. J.; Taylor R.; Allen F. H. The Nature and Geometry of Intermolecular Interactions between Halogens and Oxygen or Nitrogen. J. Am. Chem. Soc. 1996, 118, 3108–3116. 10.1021/ja953281x.

[ref17] Turunen L.; Erdélyi M. Halogen bonds of halonium ions. Chem. Soc. Rev. 2020, 49, 2688–2700. 10.1039/D0CS00034E. 32211673

[ref18] Koskinen L.; Hirva P.; Kalenius E.; Jääskeläinen S.; Rissanen K.; Haukka M. Halogen bonds with coordinative nature: halogen bonding in a S–I^+^–S iodonium complex. CrystEngComm 2015, 17, 1231–1236. 10.1039/C4CE01735H.

[ref19] Reiersølmoen A. C.; Battaglia S.; Øien-Ødegaard S.; Gupta A. K.; Fiksdahl A.; Lindh R.; Erdélyi M. Symmetry of three-center, four-electron bonds. Chem. Sci. 2020, 11, 7979–7990. 10.1039/D0SC02076A. 34094166 PMC8163095

[ref20] Lindblad S.; Németh F. B.; Földes T.; Vanderkooy A.; Pápai I.; Erdélyi M. O-I-O halogen bond of halonium ions. Chem. Commun. 2020, 56, 9671–9674. 10.1039/D0CC03513K. 32696769

[ref21] Oishi S.; Fujinami T.; Masui Y.; Suzuki T.; Kato M.; Ohtsuka N.; Momiyama N. Three-center-four-electron halogen bond enables non-metallic complex catalysis for Mukaiyama-Mannich-type reaction. iScience 2022, 25, 105220–105248. 10.1016/j.isci.2022.105220. 36274952 PMC9579028

[ref22] Warzok U.; Marianski M.; Hoffmann W.; Turunen L.; Rissanen K.; Pagel K.; Schalley C. A. Surprising solvent-induced structural rearrangements in large [N···I^+^···N] halogen-bonded supramolecular capsules: an ion mobility-mass spectrometry study. Chem. Sci. 2018, 9, 8343–8351. 10.1039/C8SC03040E. 30542581 PMC6243472

[ref23] Turunen L.; Peuronen A.; Forsblom S.; Kalenius E.; Lahtinen M.; Rissanen K. Tetrameric and Dimeric [N···I^+^···N] Halogen-Bonded Supramolecular Cages. Chem. - Eur. J. 2017, 23, 11714–11718. 10.1002/chem.201702655. 28631856

[ref24] Turunen L.; Warzok U.; Puttreddy R.; Beyeh N. K.; Schalley C. A.; Rissanen K. [N···I^+^···N] Halogen-Bonded Dimeric Capsules from Tetrakis(3-pyridyl)ethylene Cavitands. Angew. Chem., Int. Ed. 2016, 55, 14033–14036. 10.1002/anie.201607789. 27709827

[ref25] Vanderkooy A.; Gupta A. K.; Földes T.; Lindblad S.; Orthaber A.; Pápai I.; Erdélyi M. Halogen Bonding Helicates Encompassing Iodonium Cations. Angew. Chem., Int. Ed. 2019, 58, 9012–9016. 10.1002/anie.201904817. PMC6773207 31074942

[ref26] Turunen L.; Warzok U.; Schalley C. A.; Rissanen K. Nano-sized I_12_L_6_ Molecular Capsules Based on the [N···I^+^···N] Halogen Bond. Chem 2017, 3, 861–869. 10.1016/j.chempr.2017.08.010.

[ref27] An S.; Hao A.; Xing P. [N···I···N]^+^ Type Halogen-Bonding-Driven Supramolecular Helical Polymers with Modulated Chirality. ACS Nano 2022, 16, 19220–19228. 10.1021/acsnano.2c08506. 36286252

[ref28] Taipale E.; Ward J. S.; Fiorini G.; Stares D. L.; Schalley C. A.; Rissanen K. Dimeric iodine(i) and silver(i) cages from tripodal N-donor ligands *via* the [N–Ag–N]^+^ to [N–I–N]^+^ cation exchange reaction. Inorg. Chem. Front. 2022, 9, 2231–2239. 10.1039/D1QI01532J.

[ref29] Xia N.; Han J.; Xie F.; Gong G.; Wang L.; Wang J.; Chen S. Construction of Halogen-Bonded Organic Frameworks (XOFs) as Novel Efficient Iodinating Agents. ACS Appl. Mater. Interfaces 2022, 14, 43621–43627. 10.1021/acsami.2c11598. 36099250

[ref30] Gong G.; Xie F.; Wang L.; Wang J.; Chen S. Construction and Characterization of a Diphase Two-Dimensional Halogen-Bonded Organic Framework Based on a Pyrene Derivative. Synlett 2023, 34, 423–428. 10.1055/a-1928-2562.

[ref31] Gong G.; Zhao J.; Chen Y.; Xie F.; Lu F.; Wang J.; Wang L.; Chen S. An amino-type halogen-bonded organic framework for the selective adsorption of aliphatic acid vapors: insight into the competitive interactions of halogen bonds and hydrogen bonds. J. Mater. Chem. A 2022, 10, 10586–10592. 10.1039/D2TA00628F.

[ref32] Hakkert S. B.; Erdélyi M. Halogen bond symmetry: the N–X–N bond. J. Phys. Org. Chem. 2015, 28, 226–233. 10.1002/poc.3325.

[ref33] Karim A.; Reitti M.; Carlsson A.-C. C.; Gräfenstein J.; Erdélyi M. The nature of [N–Cl–N]^+^ and [N–F–N]^+^ halogen bonds in solution. Chem. Sci. 2014, 5, 3226–3233. 10.1039/C4SC01175A.

[ref34] Georgiou D. C.; Butler P.; Browne E. C.; Wilson D. J. D.; Dutton J. L. On the Bonding in Bis-pyridine Iodonium Cations. Aust. J. Chem. 2013, 66, 1179–1188. 10.1071/CH13202.

[ref35] Carlsson A.-C. C.; Gräfenstein J.; Budnjo A.; Laurila J. L.; Bergquist J.; Karim A.; Kleinmaier R.; Brath U.; Erdélyi M. Symmetric Halogen Bonding Is Preferred in Solution. J. Am. Chem. Soc. 2012, 134, 5706–5715. 10.1021/ja301341h. 22384818

[ref36] Razmazma H.; Ebrahimi A. The effects of cation-π and anion-π interactions on halogen bonds in the [N···X···N]^+^ complexes: A comprehensive theoretical study. J. Mol. Graphics Model. 2018, 84, 134–144. 10.1016/j.jmgm.2018.06.006. 29975864

[ref37] Pimentel G. C. The Bonding of Trihalide and Bifluoride Ions by the Molecular Orbital Method. J. Chem. Phys. 1951, 19, 446–448. 10.1063/1.1748245.

[ref38] Rundle R. E. Electron Deficient Compounds^1^. J. Am. Chem. Soc. 1947, 69, 1327–1331. 10.1021/ja01198a028.

[ref39] Rundle R. E. Electron Deficient Compounds. II. Relative Energies of ″Half-Bonds″. J. Chem. Phys. 1949, 17, 671–675. 10.1063/1.1747367.

[ref40] Ruedenberg K. The Physical Nature of the Chemical Bond. Rev. Mod. Phys. 1962, 34, 326–376. 10.1103/RevModPhys.34.326.

[ref41] Sonnenberg K.; Mann L.; Redeker F. A.; Schmidt B.; Riedel S. Polyhalogen and Polyinterhalogen Anions from Fluorine to Iodine. Angew. Chem., Int. Ed. 2020, 59, 5464–5493. 10.1002/anie.201903197. 31090163

[ref42] Novoa J. J.; Mota F.; Alvarez S. Structure and Stability of the X_3_^–^ Systems (X = F, Cl, Br, I) and Their Interaction with Cations. J. Phys. Chem. A 1988, 92, 6561–6566. 10.1021/j100334a017.

[ref43] Hardegger L. A.; Kuhn B.; Spinnler B.; Anselm L.; Ecabert R.; Stihle M.; Gsell B.; Thoma R.; Diez J.; Benz J.; Plancher J.-M.; Hartmann G.; Banner D. W.; Haap W.; Diederich F. Systematic Investigation of Halogen Bonding in Protein-Ligand Interactions. Angew. Chem., Int. Ed. 2011, 50, 314–318. 10.1002/anie.201006781. 21184410

[ref44] Carlsson A.-C. C.; Uhrbom M.; Karim A.; Brath U.; Gräfenstein J.; Erdélyi M. Solvent effects on halogen bond symmetry. CrystEngComm 2013, 15, 3087–3092. 10.1039/c2ce26745d.

[ref45] Bedin M.; Karim A.; Reitti M.; Carlsson A.-C. C.; Topić F.; Cetina M.; Pan F.; Havel V.; Al-Ameri F.; Sindelar V.; Rissanen K.; Gräfenstein J.; Erdélyi M. Counterion influence on the N-I-N halogen bond. Chem. Sci. 2015, 6, 3746–3756. 10.1039/C5SC01053E. 29218144 PMC5707496

[ref46] Carlsson A.-C. C.; Mehmeti K.; Uhrbom M.; Karim A.; Bedin M.; Puttreddy R.; Kleinmaier R.; Neverov A. A.; Nekoueishahraki B.; Gräfenstein J.; Rissanen K.; Erdélyi M. Substituent Effects on the [N–I–N]^+^ Halogen Bond. J. Am. Chem. Soc. 2016, 138, 9853–9863. 10.1021/jacs.6b03842. 27265247 PMC4981895

[ref47] Crawford M.-J.; Göbel M.; Karaghiosoff K.; Klapötke T. M.; Welch J. M. Does [I_3_]^+^ Act as an “[I]^+^” Donor to CH_3_CN and N_2_O? Structure of [H_3_CCN-I-NCCH_3_]^+^[AsF_6_]^−^. Inorg. Chem. 2009, 48, 9983–9985. 10.1021/ic9015492. 19788262

[ref48] Ebrahimi A.; Razmazma H.; Delarami H. S. The Nature of Halogen Bonds in [N•••X•••N]^+^ Complexes: A Theoretical Study. Phys. Chem. Res. 2016, 4, 1–15.

[ref49] Groom C. R.; Bruno I. J.; Lightfoot M. P.; Ward S. C. The Cambridge Structural Database. Acta Crystallogr., Sect. B: Struct. Sci., Cryst. Eng. Mater. 2016, 72, 171–179. 10.1107/S2052520616003954. PMC4822653 27048719

[ref50] Pritzkow H. Bis(hexamethylenetetramine)iodonium Triiodide. Acta Crystallogr., Sect. B: Struct. Crystallogr. Cryst. Chem. 1975, 31, 1505–1506. 10.1107/S0567740875005560.

[ref51] Grebe J.; Geiseler G.; Harms K.; Neumüller B.; Dehnicke K. Domino Effect in the Buildup of N-I-N-I Chains of the *N*-Iodine(triphenylphosphane)imine. Angew. Chem., Int. Ed. 1999, 38, 222–225. 10.1002/(SICI)1521-3773(19990115)38:1/2<222::AID-ANIE222>3.0.CO;2-5.

[ref52] Grebe-Metz J.; Weller F.; Dehnicke K. [(Et_3_PNI)_2_I]Cl *–* a Donor-Acceptor Complex of N-Iodo(triethylphosphane)imine with Iodine Monochloride. Z. Anorg. Allg. Chem. 2003, 629, 1110–1112. 10.1002/zaac.200300040.

[ref53] Alvarez S. A cartography of the van der Waals territories. Dalton Trans. 2013, 42, 8617–8636. 10.1039/c3dt50599e. 23632803

[ref54] Gil D. M.; Echeverría J.; Alvarez S. Tetramethylammonium Cation: Directionality and Covalency in Its Interactions with Halide Ions. Inorg. Chem. 2022, 61, 9082–9095. 10.1021/acs.inorgchem.2c00600. 35658462 PMC9214700

[ref55] Keil H.; Sonnenberg K.; Müller C.; Herbst-Irmer R.; Beckers H.; Riedel S.; Stalke D. Insights into the Topology and the Formation of a Genuine ppσ Bond: Experimental and Computed Electron Densities in Monoanionic Trichlorine [Cl_3_]^−^. Angew. Chem., Int. Ed. 2021, 60, 2569–2573. 10.1002/anie.202013727. PMC7898528 33151006

[ref56] Kaupp M.; Danovich D.; Shaik S. Chemistry is about energy and its changes: A critique of bond-length/bond-strength correlations. Coord. Chem. Rev. 2017, 344, 355–362. 10.1016/j.ccr.2017.03.002.

[ref57] Andrés J.; Ayers P. W.; Boto R. A.; Carbó-Dorca R.; Chermette H.; Cioslowski J.; Contreras-García J.; Cooper D. L.; Frenking G.; Gatti C.; Heidar-Zadeh F.; Joubert L.; Pendás Á. M.; Matito E.; Mayer I.; Misquitta A. J.; Mo Y.; Pilmé J.; Popelier P. L. A.; Rahm M.; Ramos-Cordoba E.; Salvador P.; Schwarz W. H. E.; Shahbazian S.; Silvi B.; Solà M.; Szalewicz K.; Tognetti V.; Weinhold F.; Zins É.-L. Nine Questions on Energy Decomposition Analysis. J. Comput. Chem. 2019, 40, 2248–2283. 10.1002/jcc.26003. 31251411

[ref58] Zhao L.; von Hopffgarten M.; Andrada D. M.; Frenking G. Energy decomposition analysis. WIREs Comput. Mol. Sci. 2018, 8, e1345 10.1002/wcms.1345.

[ref59] Stasyuk O. A.; Sedlak R.; Guerra C. F.; Hobza P. Comparison of the DFT-SAPT and Canonical EDA Schemes for the Energy Decomposition of Various Types of Noncovalent Interactions. J. Chem. Theory Comput. 2018, 14, 3440–3450. 10.1021/acs.jctc.8b00034. 29926727

[ref60] Levine D. S.; Head-Gordon M. Energy decomposition analysis of single bonds within Kohn–Sham density functional theory. Proc. Natl. Acad. Sci. U.S.A. 2017, 114, 12649–12656. 10.1073/pnas.1715763114. 29158379 PMC5715786

[ref61] Lao K. U.; Herbert J. M. Energy Decomposition Analysis with a Stable Charge-Transfer Term for Interpreting Intermolecular Interactions. J. Chem. Theory Comput. 2016, 12, 2569–2582. 10.1021/acs.jctc.6b00155. 27049750

[ref62] Velasquez J. D.; Echeverría J.; Alvarez S. Effect of the Substituents on the Nature and Strength of Lone-Pair–Carbonyl Interactions in Acyl Halides. Cryst. Growth Des. 2019, 19, 6511–6518. 10.1021/acs.cgd.9b00972.

[ref63] Echeverría J.; Velasquez J. D.; Alvarez S. Understanding the Interplay of Dispersion, Charge Transfer, and Electrostatics in Noncovalent Interactions: The Case of Bromine–Carbonyl Short Contacts. Cryst. Growth Des. 2020, 20, 7180–7187. 10.1021/acs.cgd.0c00791.

[ref64] Frisch M. J.; Trucks G. W.; Schlegel H. B.; Scuseria G. E.; Robb M. A.; Cheeseman J. R.; Scalmani G.; Barone V.; Petersson G. A.; Nakatsuji H.; Li X.; Caricato M.; Marenich A. V.; Bloino J.; Janesko B. G.; Gomperts R.; Mennucci B.; Hratchian H. P.; Ortiz J. V.; Izmaylov A. F.; Sonnenberg J. L.; Williams-Young D.; Ding F.; Lipparini F.; Egidi F.; Goings J.; Peng B.; Petrone A.; Henderson T.; Ranasinghe D.; Zakrzewski V. G.; Gao J.; Rega N.; Zheng G.; Liang W.; Hada M.; Ehara M.; Toyota K.; Fukuda R.; Hasegawa J.; Ishida M.; Nakajima T.; Honda Y.; Kitao O.; Nakai H.; Vreven T.; Throssell K.; Montgomery J. A. Jr.; Peralta J. E.; Ogliaro F.; Bearpark M. J.; Heyd J. J.; Brothers E. N.; Kudin K. N.; Staroverov V. N.; Keith T. A.; Kobayashi R.; Normand J.; Raghavachari K.; Rendell A. P.; Burant J. C.; Iyengar S. S.; Tomasi J.; Cossi M.; Millam J. M.; Klene M.; Adamo C.; Cammi R.; Ochterski J. W.; Martin R. L.; Morokuma K.; Farkas O.; Foresman J. B.; Fox D. J. Gaussian 16, rev. A.03; Gaussian, Inc.: Wallingford, CT, 2016.

[ref65] Grimme S.; Antony J.; Ehrlich S.; Krieg H. A consistent and accurate *ab initio* parametrization of density functional dispersion correction (DFT-D) for the 94 elements H-Pu. J. Chem. Phys. 2010, 132, 154104–154122. 10.1063/1.3382344. 20423165

[ref66] Grimme S.; Ehrlich S.; Goerigk L. Effect of the Damping Function in Dispersion Corrected Density Functional Theory. J. Comput. Chem. 2011, 32, 1456–1465. 10.1002/jcc.21759. 21370243

[ref67] Boys S. F.; Bernardi F. The calculation of small molecular interactions by the differences of separate total energies. Some procedures with reduced errors. Mol. Phys. 1970, 19, 553–566. 10.1080/00268977000101561.

[ref68] Dennington R.; Keith T. A.; Millam J. M. GaussView. version 6; Semichem Inc.: Shawnee Mission, KS, 2016.

[ref69] Shao Y.; Gan Z.; Epifanovsky E.; Gilbert A. T. B.; Wormit M.; Kussmann J.; Lange A. W.; Behn A.; Deng J.; Feng X.; Ghosh D.; Goldey M.; Horn P. R.; Jacobson L. D.; Kaliman I.; Khaliullin R. Z.; Kuś T.; Landau A.; Liu J.; Proynov E. I.; Rhee Y. M.; Richard R. M.; Rohrdanz M. A.; Steele R. P.; Sundstrom E. J.; Woodcock H. L. III; Zimmerman P. M.; Zuev D.; Albrecht B.; Alguire E.; Austin B.; Beran G. J. O.; Bernard Y. A.; Berquist E.; Brandhorst K.; Bravaya K. B.; Brown S. T.; Casanova D.; Chang C.-M.; Chen Y.; Chien S. H.; Closser K. D.; Crittenden D. L.; Diedenhofen M.; DiStasio R. A. Jr; Do H.; Dutoi A. D.; Edgar R. G.; Fatehi S.; Fusti-Molnar L.; Ghysels A.; Golubeva-Zadorozhnaya A.; Gomes J.; Hanson-Heine M. W. D.; Harbach P. H. P.; Hauser A. W.; Hohenstein E. G.; Holden Z. C.; Jagau T.-C.; Ji H.; Kaduk B.; Khistyaev K.; Kim J.; Kim J.; King R. A.; Klunzinger P.; Kosenkov D.; Kowalczyk T.; Krauter C. M.; Lao K. U.; Laurent A. D.; Lawler K. V.; Levchenko S. V.; Lin C. Y.; Liu F.; Livshits E.; Lochan R. C.; Luenser A.; Manohar P.; Manzer S. F.; Mao S.-P.; Mardirossian N.; Marenich A. V.; Maurer S. A.; Mayhall N. J.; Neuscamman E.; Oana C. M.; Olivares-Amaya R.; O’Neill D. P.; Parkhill J. A.; Perrine T. M.; Peverati R.; Prociuk A.; Rehn D. R.; Rosta E.; Russ N. J.; Sharada S. M.; Sharma S.; Small D. W.; Sodt A.; Stein T.; Stück D.; Su Y.-C.; Thom A. J. W.; Tsuchimochi T.; Vanovschi V.; Vogt L.; Vydrov O.; Wang T.; Watson M. A.; Wenzel J.; White A.; Williams C. F.; Yang J.; Yeganeh S.; Yost S. R.; You Z.-Q.; Zhang I. Y.; Zhang X.; Zhao Y.; Brooks B. R.; Chan G. K. L.; Chipman D. M.; Cramer C. J.; Goddard W. A. III; Gordon M. S.; Hehre W. J.; Klamt A.; Schaefer H. F. III; Schmidt M. W.; Sherrill C. D.; Truhlar D. G.; Warshel A.; Xu X.; Aspuru-Guzik A.; Baer R.; Bell A. T.; Besley N. A.; Chai J.-D.; Dreuw A.; Dunietz B. D.; Furlani T. R.; Gwaltney S. R.; Hsu C.-P.; Jung Y.; Kong J.; Lambrecht D. S.; Liang W.; Ochsenfeld C.; Rassolov V. A.; Slipchenko L. V.; Subotnik J. E.; Van Voorhis T.; Herbert J. M.; Krylov A. I.; Gill P. M. W.; Head-Gordon M. Advances in Molecular Quantum Chemistry Contained in the Q-Chem 4 Program Package. Mol. Phys. 2015, 113, 184–215. 10.1080/00268976.2014.952696.

[ref70] Horn P. R.; Mao Y.; Head-Gordon M. Probing non-covalent interactions with a second generation energy decomposition analysis using absolutely localized molecular orbitals. Phys. Chem. Chem. Phys. 2016, 18, 23067–23079. 10.1039/C6CP03784D. 27492057

