# Supplementary material for: Structure and Bonding of Halonium Compounds
Source: Inorg Chem. 2023 May 31;62(23):8980–92. doi: 10.1021/acs.inorgchem.3c00654 (PMC10265704; doi:10.1021/acs.inorgchem.3c00654)
Supplement: Supplementary file 1 — ic3c00654_si_001.pdf [file ic3c00654_si_001.pdf]

Structure and bonding of halonium compounds

Juan D. Velasquez,[a] Jorge Echeverría[a] and Santiago Alvarez*[b]

[a]Instituto de Síntesis Química y Catálisis Homogénea (ISQCH) and Departamento de Química Inorgánica, Facultad de Ciencias, Universidad de Zaragoza, Pedro Cerbuna 12, 50009 Zaragoza (Spain).

[b]Departament de Química Inorgànica i Orgànica and Institut de Química Teòrica i Computacional (IQTC-UB), Universitat de Barcelona, Martí i Franquès 1-11, 08028 Barcelona (Spain).

SUPPORTING INFORMATION

Table S1 [Py]···[X-Py]$^+$ adducts.

X = F

H	-2.156020	1.453881	1.455619
H	-5.914950	0.004386	-0.005030
H	-4.630841	1.521173	1.517758
H	-4.628061	-1.514040	-1.523833
H	-2.153369	-1.449932	-1.454001
C	-2.737054	-0.799356	-0.803494
C	-4.120627	-0.837085	-0.844346
C	-2.738522	0.804137	0.803223
C	-4.122166	0.843610	0.839808
C	-4.828510	0.003710	-0.003360
N	-2.045088	0.001961	0.000922
F	0.744530	-0.009820	0.022942
N	2.081250	-0.006175	0.012047
C	2.689911	-0.846709	0.847009
C	4.066004	-0.852305	0.844770
C	4.748983	0.001224	-0.010441
C	4.046949	0.850895	-0.853997
C	2.671047	0.837649	-0.833050
H	4.587243	-1.527461	1.515205
H	2.041614	-1.461845	1.464175
H	2.008854	1.448976	-1.439163
H	4.553043	1.528887	-1.533134
H	5.835779	0.004238	-0.019629

X = Cl

H	-1.991857	2.056347	0.030193
H	-5.771586	0.000000	0.000000
H	-4.498218	2.153202	0.031489
H	-4.498218	-2.153202	-0.031489
H	-1.991858	-2.056347	-0.030193
C	-2.607096	-1.160475	-0.017028
C	-3.986641	-1.196772	-0.017518
C	-2.607096	1.160475	0.017027
C	-3.986641	1.196772	0.017518
C	-4.685115	0.000000	0.000000
N	-1.961301	0.000000	0.000000
Cl	0.000000	0.000000	0.000000
N	1.961301	0.000000	0.000000
C	2.607096	-1.160475	0.017028
C	3.986641	-1.196772	0.017518
C	4.685115	0.000000	0.000000
C	3.986641	1.196772	-0.017518
C	2.607096	1.160475	-0.017027
H	4.498218	-2.153202	0.031489
H	1.991858	-2.056347	0.030193

H	1.991857	2.056347	-0.030193
H	4.498218	2.153202	-0.031489
H	5.771586	0.000000	0.000000

X = Br

H	-2.130793	2.058683	0.000000
H	-5.906237	0.000001	0.000001
H	-4.630274	2.152875	0.000000
H	-4.630276	-2.152874	0.000000
H	-2.130795	-2.058684	0.000000
C	-2.740922	-1.159306	0.000000
C	-4.120007	-1.195782	0.000000
C	-2.740921	1.159305	0.000000
C	-4.120006	1.195783	0.000000
C	-4.819777	0.000001	0.000000
N	-2.087593	-0.000001	0.000000
Br	0.000000	-0.000002	-0.000001
N	2.087593	-0.000001	0.000000
C	2.740922	-1.159306	0.000000
C	4.120007	-1.195782	0.000000
C	4.819777	0.000001	0.000000
C	4.120006	1.195783	0.000000
C	2.740921	1.159305	0.000000
H	4.630276	-2.152874	0.000000
H	2.130795	-2.058684	0.000000
H	2.130793	2.058683	0.000000
H	4.630274	2.152875	0.000000
H	5.906237	0.000001	0.000001

X = I

H	-2.314077	2.061070	0.030271
H	-6.084079	0.000004	0.000000
H	-4.805533	2.152323	0.031467
H	-4.805539	-2.152320	-0.031466
H	-2.314083	-2.061074	-0.030271
C	-2.917785	-1.157251	-0.016971
C	-4.296461	-1.194586	-0.017479
C	-2.917782	1.157248	0.016971
C	-4.296458	1.194588	0.017479
C	-4.997606	0.000002	0.000000
N	-2.254690	-0.000002	0.000000
I	0.000000	-0.000005	0.000001
N	2.254690	-0.000002	0.000000
C	2.917785	-1.157251	0.016971
C	4.296461	-1.194586	0.017479
C	4.997606	0.000002	0.000000
C	4.296458	1.194588	-0.017479
C	2.917782	1.157248	-0.016971

H	4.805539	-2.152320	0.031466
H	2.314083	-2.061074	0.030271
H	2.314077	2.061070	-0.030271
H	4.805533	2.152323	-0.031467
H	6.084079	0.000004	0.000000

Table S2. [D]···[I-D]$^+$ adducts.

D = Imidazole ($C_3H_4N_2$)

H	-2.624562	2.169112	0.003940
H	-5.255539	1.320756	-0.002439
H	-5.097239	-1.215170	-0.014953
H	-2.693293	-2.028598	-0.016933
C	-3.036213	1.171455	-0.001408
C	-4.330327	0.766117	-0.004440
C	-3.019281	-0.998112	-0.012121
N	-4.294928	-0.601487	-0.011384
N	-2.236924	0.059038	-0.006189
I	0.000689	0.041301	-0.005133
N	2.238307	0.023560	-0.004309
N	4.296275	0.684098	-0.003161
C	3.037609	-1.088858	-0.006272
C	4.331716	-0.683515	-0.005385
C	3.020628	1.080741	-0.002442
H	2.694612	2.111226	-0.000801
H	5.098562	1.297820	-0.001707
H	5.256931	-1.238156	-0.006100
H	2.626012	-2.086549	-0.008177

D = Pyrimidine ($C_4H_4N_2$)

H	-2.373620	-1.514386	-1.457738
H	-6.024160	-0.061530	-0.217527
H	-4.862421	1.617794	1.274651
H	-2.337223	1.587014	1.276517
C	-2.950592	-0.818438	-0.850063
C	-4.937715	-0.006861	-0.157044
C	-4.306390	0.916183	0.663234
C	-2.930187	0.905813	0.670009
N	-2.260801	0.035403	-0.089048
N	-4.260207	-0.868142	-0.908487
I	-0.000251	-0.001893	-0.102715
N	2.260314	-0.040085	-0.110251
N	4.251844	0.803868	-1.008581
C	2.942861	0.760379	-0.933289
C	2.936885	-0.859245	0.697878
C	4.312919	-0.872347	0.677423
C	4.936439	-0.006943	-0.208924
H	2.360239	1.415454	-1.579783

H	6.022233	0.041736	-0.284347
H	2.349739	-1.497638	1.354549
H	4.874616	-1.532776	1.328251

D = Acetonitrile (NCMe)

H	0.837967	-0.599661	-5.136953
H	0.101479	1.026306	-5.136047
H	-0.938475	-0.424508	-5.137452
C	0.000198	0.000460	-4.772367
C	-0.000167	-0.000615	-3.331675
N	-0.000298	-0.001066	-2.190170
I	-0.000184	-0.000534	0.000099
N	0.000450	0.000057	2.190021
C	0.000368	0.000247	3.331528
C	0.000136	0.000105	4.772212
H	0.938801	0.425319	5.136959
H	-0.837647	0.600475	5.136484
H	-0.101124	-1.025547	5.136566

D = Ammonia (NH_3)

H	2.485280	1.177875	-0.280284
H	2.518970	-0.467194	-0.453017
H	2.408874	0.204160	1.055126
N	2.115034	0.300160	0.083422
I	-0.178123	0.268984	-0.070152
N	-2.471309	0.237371	-0.223039
H	-2.889990	0.663564	0.603299
H	-2.810732	-0.721189	-0.298579
H	-2.781351	0.754990	-1.045046

Table S3. $[(2\text{-R-py})_2I]^+$ adducts.

R = -OH

H	-2.137746	-1.350421	-1.707344
H	-5.021808	1.096643	1.394408
H	-4.608576	-1.559346	-1.968486
H	-6.079154	-0.298211	-0.369538
H	-2.957927	1.773439	2.251738
C	-2.829723	-0.831054	-1.049497
C	-4.189547	-0.939704	-1.184941
C	-3.032529	0.597252	0.758747
C	-4.421126	0.536673	0.684691
C	-4.996802	-0.238114	-0.295315
O	-2.363431	1.307107	1.654994
N	-2.265608	-0.076728	-0.095680
I	0.000472	-0.001097	0.002498
N	2.267074	0.111454	-0.041688

O	2.360751	-1.958322	0.792910
C	3.032007	-0.898448	0.367502
C	4.420748	-0.810995	0.335340
C	4.998652	0.346977	-0.130775
C	4.193493	1.398390	-0.556815
C	2.833335	1.238612	-0.495290
H	2.142846	2.016535	-0.810557
H	5.019753	-1.650033	0.674910
H	4.614312	2.324286	-0.929775
H	6.081181	0.434406	-0.163961
H	2.953714	-2.659766	1.081655

R = -OMe

H	-2.185344	-1.614078	1.835328
H	-5.044669	1.383011	-0.767131
H	-4.660606	-1.859254	2.030459
H	-6.108179	-0.310191	0.686046
H	-3.655316	2.042626	-2.519058
H	-2.269461	3.138365	-2.272516
H	-3.664665	3.218309	-1.163689
C	-2.869564	-0.976818	1.280785
C	-4.232474	-1.104536	1.382068
C	-3.050667	0.773138	-0.229682
C	-4.441165	0.704060	-0.178555
C	-5.024780	-0.243216	0.634619
C	-3.040359	2.554698	-1.771384
O	-2.351443	1.622715	-0.958367
N	-2.296386	-0.060270	0.493979
I	-0.027976	0.017223	0.443165
N	2.237686	0.100769	0.563220
O	2.336673	-1.701247	-0.735954
C	3.049897	-2.701559	-1.439476
C	3.013450	-0.791461	-0.060713
C	4.401675	-0.717580	0.029994
C	4.960520	0.295786	0.778221
C	4.146043	1.217269	1.423091
C	2.786901	1.081193	1.287660
H	3.691030	-2.257847	-2.208533
H	2.294648	-3.327643	-1.912433
H	3.651563	-3.308400	-0.754507
H	2.086387	1.763447	1.762691
H	5.022599	-1.443893	-0.478441
H	4.554392	2.024592	2.018762
H	6.041799	0.367436	0.859358

R = -NH_2

H	-2.106721	-1.394400	-1.702856
H	-5.063994	1.262671	1.137596

H	-4.560029	-1.636306	-2.003495
H	-6.072066	-0.261307	-0.530183
H	-1.484318	1.713332	1.533305
H	-3.058417	2.172539	2.057684
C	-3.046295	0.744964	0.605690
C	-4.444410	0.653927	0.487434
C	-2.813012	-0.830284	-1.099560
C	-4.161802	-0.958285	-1.259072
C	-4.991771	-0.191131	-0.436977
N	-2.478085	1.552509	1.518118
N	-2.263563	-0.002337	-0.190240
I	-0.003395	0.012513	-0.115859
N	2.257719	0.022809	-0.164437
N	2.450683	-1.436653	1.628618
C	3.030326	-0.681013	0.679560
C	4.429798	-0.598494	0.571960
C	4.988903	0.194027	-0.391136
C	4.169422	0.916440	-1.262813
C	2.818699	0.799214	-1.111413
H	5.041021	-1.171797	1.261045
H	2.120160	1.330617	-1.752270
H	4.577124	1.552439	-2.038558
H	6.070280	0.257449	-0.476259
H	1.456725	-1.596642	1.640339
H	3.024071	-2.027872	2.206578

R = -CH_3

H	-2.080237	-1.376026	-1.890395
H	-5.050613	1.237770	0.963139
H	-4.534828	-1.591818	-2.231829
H	-6.060867	-0.244471	-0.759709
H	-3.173576	2.129118	2.043171
H	-1.753834	2.335081	1.002129
H	-1.801400	1.014523	2.168673
C	-2.411270	1.598805	1.473563
C	-3.045364	0.718662	0.451551
C	-4.424815	0.639943	0.308676
C	-2.788357	-0.817651	-1.283377
C	-4.146950	-0.930666	-1.465144
C	-4.981598	-0.184218	-0.649273
N	-2.260657	-0.016841	-0.352486
I	0.007666	-0.000956	-0.251163
N	2.277507	0.012339	-0.307761
C	3.045518	-0.705599	0.527675
C	4.427586	-0.631087	0.410091
C	5.003945	0.171058	-0.554931
C	4.186245	0.899769	-1.403381
C	2.824233	0.791842	-1.245752
C	2.390660	-1.562346	1.556461

H	1.767600	-0.962440	2.226110
H	3.141148	-2.079955	2.152802
H	1.742126	-2.308836	1.088787
H	5.039757	-1.214662	1.089795
H	2.128741	1.337051	-1.878772
H	4.589816	1.543415	-2.176839
H	6.085230	0.228012	-0.645606

R = -NMe$_2$

H	-1.525078	-2.334046	1.114620
H	-5.327689	0.419533	-0.217130
H	-3.754239	-3.413487	0.908945
H	-5.685682	-1.986490	0.152327
H	-1.780165	1.724584	1.816777
H	-2.922464	3.066067	1.650022
H	-1.454296	3.019882	0.647944
H	-4.297136	1.985867	-1.514839
H	-3.478385	3.423496	-0.894963
H	-4.827684	2.729897	0.020279
C	-3.226765	0.343618	0.248386
C	-4.498029	-0.219614	0.057465
C	-2.406444	-1.765290	0.829179
C	-3.633935	-2.357379	0.701327
C	-4.694270	-1.560041	0.279886
C	-2.238714	2.405577	1.100814
C	-3.956880	2.485050	-0.605769
N	-2.981117	1.674509	0.087271
N	-2.197158	-0.462918	0.584013
I	-0.015518	0.026122	0.141819
N	2.165760	0.515092	-0.300277
N	2.949533	-1.622668	0.195357
C	2.207278	-2.353326	-0.818633
C	3.925317	-2.433592	0.887939
C	3.195359	-0.291759	0.034664
C	4.466735	0.271237	0.225463
C	4.663107	1.611717	0.003485
C	3.602771	2.409374	-0.417359
C	2.375148	1.817541	-0.545035
H	5.296386	-0.368107	0.499633
H	1.493769	2.386510	-0.829994
H	3.723197	3.465534	-0.624647
H	5.654616	2.037979	0.130921
H	4.265735	-1.934829	1.797175
H	4.796003	-2.678262	0.261659
H	3.446755	-3.372108	1.176790
H	1.748146	-1.672028	-1.533920
H	1.423347	-2.968411	-0.365995
H	2.891260	-3.012999	-1.368548

R = -CH$_2$F

H	-2.074915	-1.582631	1.755586
H	-5.091836	1.073726	-1.014992
H	-4.526040	-1.900219	2.037968
H	-6.079607	-0.528939	0.622610
H	-1.790766	2.256205	-0.942735
H	-3.240041	2.114004	-1.977687
C	-2.459218	1.551729	-1.455651
C	-3.084491	0.599611	-0.474835
C	-4.457888	0.471634	-0.371818
C	-2.790400	-1.011983	1.168708
C	-4.151684	-1.180883	1.317882
C	-5.001959	-0.421807	0.535412
F	-1.727721	0.852920	-2.376477
N	-2.277574	-0.139530	0.300968
I	-0.002915	-0.013749	0.282924
N	2.264989	0.127957	0.396992
F	1.858021	-2.531375	-0.698614
C	3.097686	-0.561179	-0.397825
C	4.465123	-0.376949	-0.298312
C	4.976132	0.521271	0.622969
C	4.099986	1.213549	1.438581
C	2.746712	0.987023	1.295407
C	2.508958	-1.538330	-1.377280
H	3.305877	-1.985303	-1.980217
H	1.790359	-1.040953	-2.042577
H	5.120957	-0.950504	-0.945554
H	2.011850	1.503368	1.907838
H	4.448643	1.922953	2.181076
H	6.048800	0.671765	0.707554

R = -CHF_2

H	-1.651733	-2.431081	-0.206512
H	-5.327093	0.711755	0.462615
H	-3.972604	-3.320492	-0.163622
H	-5.868150	-1.712010	0.179741
H	-3.820242	2.453808	0.587944
C	-2.902836	1.864166	0.446739
C	-3.227875	0.394124	0.275058
C	-4.542333	-0.023008	0.311042
C	-2.505161	-1.773281	-0.060931
C	-3.798816	-2.257576	-0.035403
C	-4.838106	-1.367796	0.153980
F	-2.251358	2.324893	-0.633291
F	-2.103324	2.041662	1.510806
N	-2.228773	-0.478166	0.090785
I	0.006490	-0.003848	-0.001380
N	2.241570	0.470126	-0.093990
F	2.117060	-2.053741	-1.506943

F	2.263413	-2.331092	0.638012
C	3.240738	-0.402827	-0.275081
C	4.555223	0.014119	-0.311441
C	4.851021	1.359416	-0.158238
C	3.811698	2.249790	0.027864
C	2.517966	1.765631	0.054106
C	2.915748	-1.873333	-0.442742
H	3.833236	-2.463417	-0.581552
H	5.340051	-0.721132	-0.460257
H	1.664542	2.423950	0.197366
H	3.985387	3.313085	0.153020
H	5.881127	1.703412	-0.184413

R = -F

H	-2.307661	2.035642	0.212311
H	-6.101601	0.025016	0.014698
H	-4.799180	2.155814	0.236032
H	-4.840096	-2.139421	-0.221418
C	-2.962365	-1.134466	-0.122406
C	-4.340732	-1.182414	-0.121542
C	-2.927238	1.147688	0.120558
C	-4.299225	1.199757	0.132103
C	-5.015110	0.014368	0.008906
F	-2.257750	-2.228307	-0.241792
N	-2.269236	-0.014934	-0.006106
I	-0.000185	-0.001379	-0.018766
N	2.269281	0.013636	-0.006729
F	2.256289	2.207024	-0.385469
C	2.927936	-1.137909	0.195193
C	4.299965	-1.188276	0.210161
C	5.015133	-0.012962	0.010135
C	4.340058	1.172454	-0.197765
C	2.961726	1.123654	-0.195416
H	4.800564	-2.135173	0.376162
H	2.308893	-2.018425	0.344368
H	4.838903	2.121175	-0.359779
H	6.101631	-0.022575	0.016561

R = -Cl

H	-2.201568	1.596652	1.120539
H	-6.098516	0.055974	0.180518
H	-4.673981	1.762947	1.350452
H	-4.969294	-1.728964	-1.157537
C	-3.020060	-0.983896	-0.705314
C	-4.404046	-0.967379	-0.632331
C	-2.867644	0.886363	0.638136
C	-4.233952	0.968826	0.758055
C	-5.014767	0.022754	0.109340

Cl	-2.234630	-2.192918	-1.613685
N	-2.271751	-0.074571	-0.081654
I	0.000427	-0.000304	-0.145791
N	2.272125	0.074259	-0.070199
Cl	2.242537	2.190410	-1.605254
C	2.864493	-0.885558	0.653989
C	4.230180	-0.967618	0.781005
C	5.014149	-0.022384	0.134870
C	4.407060	0.966555	-0.611355
C	3.023456	0.982803	-0.691402
H	4.667301	-1.760790	1.376820
H	2.196078	-1.595273	1.134000
H	4.974877	1.727437	-1.134803
H	6.097527	-0.055332	0.211599

R = -SO_3H (cis)

H	-6.110660	-1.204932	0.089886
H	-4.922021	1.002147	-0.020174
H	-4.730001	-3.298905	0.174792
H	-0.502684	1.712701	-1.312508
H	-2.252300	-3.091306	0.142491
C	-2.892716	-2.213750	0.108697
C	-4.270458	-2.317431	0.125634
C	-2.998388	0.088896	0.005239
C	-4.378331	0.064239	0.017678
C	-5.025011	-1.161706	0.079107
O	-3.256036	2.631038	-0.078750
O	-1.234364	1.751489	1.006371
O	-1.483864	1.675894	-1.396300
S	-2.205673	1.700119	-0.039341
N	-2.272437	-1.035322	0.051116
I	-0.000007	-1.089555	-0.000029
N	2.272421	-1.035431	-0.051188
S	2.205693	1.699994	0.038828
O	3.255994	2.630987	0.078082
O	1.234368	1.751152	-1.006879
O	1.483869	1.676015	1.395784
C	2.998410	0.088765	-0.005408
C	4.378353	0.064059	-0.017723
C	5.024992	-1.161921	-0.078929
C	4.270398	-2.317623	-0.125397
C	2.892659	-2.213893	-0.108577
H	6.110640	-1.205194	-0.089573
H	4.729910	-3.299119	-0.174421
H	4.922073	1.001952	0.020033
H	0.502698	1.712926	1.312008
H	2.252212	-3.091429	-0.142304

R = -SO_3H (trans)

H	-0.932252	2.780720	0.167370
H	-5.200415	3.253610	0.011649
H	-2.916559	4.278458	0.187235
H	-5.370146	0.753468	-0.168371
H	-3.478436	-1.950093	1.723616
C	-3.256295	0.502077	-0.098596
C	-4.411908	1.256416	-0.090970
C	-1.941286	2.382406	0.090978
C	-3.051999	3.205387	0.103801
C	-4.306429	2.636122	0.008143
O	-4.790238	-1.555522	-0.275055
O	-2.534659	-1.677949	-1.302591
O	-2.787382	-1.787883	1.064810
S	-3.407080	-1.271070	-0.275344
N	-2.042079	1.056896	-0.015066
I	-0.006703	0.007475	-0.004133
N	2.027841	-1.041266	0.007094
S	3.392743	1.287785	0.261007
O	2.521576	1.696630	1.288545
O	4.775853	1.572399	0.258551
O	2.771589	1.801556	-1.079612
C	1.927434	-2.367073	-0.095960
C	3.038401	-3.189668	-0.108086
C	4.292728	-2.619754	-0.015025
C	4.397793	-1.239806	0.080954
C	3.241968	-0.485791	0.088195
H	2.903249	-4.262963	-0.189067
H	0.918462	-2.765863	-0.170517
H	5.186929	-3.236932	-0.018177
H	5.355930	-0.736331	0.156183
H	3.462167	1.964084	-1.738842

R = -CN

H	-6.030435	-0.961777	-0.001344
H	-4.391088	-2.858895	-0.002195
H	-1.955645	-2.333748	-0.002649
H	-5.145746	1.382257	-0.001014
C	-2.574191	2.055221	-0.001484
C	-2.706230	-1.546756	-0.002284
C	-4.060281	-1.825753	-0.002027
C	-3.112208	0.730677	-0.001653
C	-4.481709	0.524121	-0.001373
C	-4.960150	-0.775254	-0.001561
N	-2.164293	3.126634	-0.001343
N	-2.246822	-0.297920	-0.002098
I	-0.000057	-0.000277	-0.002451
N	2.246742	0.297414	-0.002761
N	2.164372	-3.127088	-0.003124

C	3.112193	-0.731125	-0.002982
C	4.481681	-0.524462	-0.003071
C	4.960023	0.774945	-0.002933
C	4.060080	1.825384	-0.002718
C	2.706048	1.546281	-0.002638
C	2.574248	-2.055667	-0.003139
H	1.955407	2.333220	-0.002471
H	4.390821	2.858547	-0.002611
H	6.030295	0.961541	-0.002995
H	5.145781	-1.382553	-0.003252

R = -CF_3

H	-1.571408	-2.490884	-0.017583
H	-5.377385	0.573549	0.001457
H	-3.862666	-3.453633	-0.020356
H	-5.822627	-1.886644	-0.010468
C	-2.963786	1.801695	0.005345
C	-3.262166	0.306740	-0.001871
C	-4.564992	-0.144176	-0.002901
C	-2.451376	-1.852710	-0.013528
C	-3.728853	-2.377217	-0.014987
C	-4.803628	-1.509891	-0.009553
F	-2.263373	2.140759	1.079184
F	-4.083185	2.497366	0.009137
F	-2.264203	2.151217	-1.065727
N	-2.224785	-0.538456	-0.007082
I	0.000181	-0.002961	-0.006211
N	2.225139	0.532525	-0.005606
F	2.264666	-2.156146	1.055883
F	4.083684	-2.503226	-0.018579
F	2.263925	-2.147828	-1.089055
C	3.262554	-0.312643	-0.009804
C	4.565354	0.138325	-0.009123
C	4.803955	1.504062	-0.003857
C	3.729157	2.371348	0.000519
C	2.451691	1.846784	-0.000550
C	2.964246	-1.807623	-0.015490
H	5.377786	-0.579360	-0.012679
H	1.571718	2.484956	0.002704
H	3.862895	3.447779	0.004747
H	5.822952	1.880822	-0.003178

R = Br

H	-2.168782	1.549088	1.151254
H	-6.093752	0.061478	0.245709
H	-4.633519	1.726994	1.433655
H	-5.006408	-1.691109	-1.159849
C	-3.039042	-0.975535	-0.728465

C	-4.421614	-0.950098	-0.626869
C	-2.848814	0.856639	0.662509
C	-4.211638	0.945861	0.811429
C	-5.011739	0.023384	0.153038
Br	-2.231261	-2.282942	-1.780136
N	-2.272871	-0.088823	-0.094777
I	0.000022	-0.001820	-0.173745
N	2.273054	0.087283	-0.093729
Br	2.237612	2.202866	-1.876033
C	2.846253	-0.823574	0.706762
C	4.208717	-0.908547	0.861279
C	5.011364	-0.018970	0.161984
C	4.424055	0.919034	-0.662261
C	3.041590	0.942776	-0.766329
H	4.628327	-1.661068	1.519262
H	2.164274	-1.491449	1.226008
H	5.010959	1.633568	-1.228053
H	6.093213	-0.055045	0.257359

R = I

H	-2.141382	1.486817	1.203140
H	-6.087274	0.059653	0.298303
H	-4.598662	1.672341	1.526256
H	-5.036127	-1.643716	-1.179873
C	-3.049194	-0.967416	-0.759055
C	-4.432104	-0.929381	-0.631873
C	-2.832173	0.816845	0.698445
C	-4.191829	0.911095	0.870194
C	-5.006746	0.018587	0.190120
I	-2.212053	-2.398050	-2.000008
N	-2.272984	-0.104804	-0.100354
I	-0.000193	0.000112	-0.181164
N	2.272903	0.104998	-0.103206
I	2.210455	2.393869	-2.007963
C	2.832678	-0.814970	0.697124
C	4.192489	-0.909245	0.867598
C	5.006922	-0.018554	0.184561
C	4.431689	0.927697	-0.638985
C	3.048628	0.965869	-0.764731
H	4.599830	-1.669091	1.524966
H	2.142237	-1.483549	1.204141
H	5.035333	1.640586	-1.189284
H	6.087558	-0.059704	0.291649

R = NO_2

H	-1.635433	2.424391	0.303820
H	-5.855286	1.826525	-0.253277
H	-3.916509	3.383408	0.093418

H	-5.383876	-0.650435	-0.346972
C	-3.303412	-0.342321	-0.033445
C	-4.600936	0.084276	-0.194677
C	-2.505688	1.785864	0.174893
C	-3.778430	2.308347	0.047440
C	-4.843158	1.447225	-0.143714
O	-3.847599	-2.473423	-0.626570
O	-2.087938	-2.184225	0.592418
N	-3.050354	-1.793596	-0.027678
N	-2.268544	0.471079	0.143682
I	-0.010581	0.006907	0.188621
N	2.247548	-0.457967	0.146177
N	3.026862	1.799988	-0.105776
O	2.068116	2.210710	0.506972
O	3.819906	2.459811	-0.732014
C	2.485657	-1.770869	0.220066
C	3.757922	-2.296826	0.102488
C	4.820929	-1.442175	-0.124010
C	4.577582	-0.081800	-0.219252
C	3.280799	0.349430	-0.064535
H	3.896963	-3.369682	0.183614
H	1.616553	-2.405054	0.375553
H	5.832587	-1.824556	-0.226980
H	5.359106	0.647717	-0.400883

Table S4. $[(2,6\text{-}R_2\text{-py})_2I]^+$ adducts.

R = -SO_3H

H	-6.090407	-1.064328	0.337112
H	-5.201637	1.282175	0.238496
H	-4.443529	-2.956363	0.218353
H	-1.042044	2.659727	-1.289856
H	-0.066117	-2.789620	-1.314927
C	-2.772197	-1.638498	0.110001
C	-4.124095	-1.920108	0.200886
C	-3.177291	0.626531	0.120531
C	-4.542802	0.421126	0.211830
C	-5.023340	-0.873134	0.261757
O	-2.548611	-4.185926	0.031062
O	-0.688140	-2.952633	1.045179
O	-3.851718	3.093884	0.067379
O	-1.676798	2.572817	1.071705
O	-2.003879	2.442947	-1.325007
O	-1.043450	-2.919713	-1.350519
S	-2.659051	2.354152	0.059323
S	-1.686837	-3.078260	0.033035
N	-2.297588	-0.384747	0.084148
I	-0.024099	0.022780	-0.003063
N	2.249628	0.430455	-0.090198

S	2.611242	-2.308426	-0.065455
S	1.638857	3.123942	-0.038758
O	1.629005	-2.527224	-1.077866
O	3.803887	-3.048128	-0.073405
O	2.500669	4.231575	-0.036466
O	0.640201	2.998645	-1.050983
O	0.995361	2.965096	1.344721
O	1.955983	-2.397215	1.318846
C	2.724209	1.684205	-0.116078
C	4.076077	1.965882	-0.207154
C	4.975357	0.918953	-0.268183
C	4.494879	-0.375322	-0.218151
C	3.129385	-0.580779	-0.126701
H	6.042403	1.110189	-0.343700
H	5.153765	-1.236331	-0.244862
H	4.395456	3.002155	-0.224628
H	0.017991	2.835220	1.308985
H	0.994142	-2.613853	1.283665

R = -OH

H	-4.883811	1.523709	-1.531774
H	-4.881020	-1.531436	1.500439
H	-6.140355	-0.004256	-0.014914
H	-2.737483	2.151649	-2.155344
H	-2.733540	-2.157929	2.121523
C	-2.983261	-0.823234	0.797011
C	-4.367454	-0.854012	0.827969
C	-2.984757	0.816722	-0.830615
C	-4.369004	0.846625	-0.859910
C	-5.053902	-0.003915	-0.015567
O	-2.208249	-1.585321	1.553144
O	-2.211142	1.579336	-1.587641
N	-2.308513	-0.003043	-0.017204
I	-0.015664	-0.002247	-0.018601
N	2.277131	-0.001294	-0.020047
O	2.177989	-1.572691	-1.601370
O	2.178654	1.569990	1.561420
C	2.952469	-0.815273	-0.840022
C	4.336659	-0.844721	-0.871302
C	5.022517	0.000098	-0.021985
C	4.337030	0.844187	0.828310
C	2.952796	0.813333	0.798976
H	4.850732	-1.516986	-1.548546
H	4.851338	1.517015	1.504815
H	6.108970	0.000643	-0.022792
H	2.703672	-2.140930	-2.173759
H	2.704596	2.138651	2.133149

R = -OMe

H	-4.733901	1.926596	-1.505157
H	-4.993630	-1.138974	1.486930
H	-6.097354	0.496783	-0.010669
H	-3.187373	2.259523	-3.201942
H	-1.666350	3.154398	-2.939863
H	-3.084843	3.505490	-1.916360
H	-3.630741	-2.968982	1.903110
H	-3.528614	-1.722023	3.187812
H	-2.176352	-2.857007	2.930447
C	-3.026338	-0.594764	0.802771
C	-4.411074	-0.508958	0.829706
C	-2.885756	1.063724	-0.816269
C	-4.265521	1.208621	-0.846824
C	-5.014434	0.406311	-0.009346
C	-2.537875	2.721367	-2.450709
C	-2.962733	-2.285985	2.438637
O	-2.302640	-1.415400	1.541095
O	-2.033859	1.752877	-1.552227
N	-2.287822	0.178790	-0.005835
I	0.000223	-0.007614	0.003601
N	2.288415	-0.193718	0.018877
O	2.051360	-1.769325	-1.528640
O	2.286363	1.401897	1.564398
C	2.936643	2.274056	2.467547
C	2.565114	-2.738528	-2.420824
C	2.895213	-1.079048	-0.784501
C	4.275297	-1.223242	-0.800583
C	5.015065	-0.419883	0.044013
C	4.402519	0.495745	0.875971
C	3.018094	0.580886	0.834440
H	3.221929	-2.277130	-3.165947
H	1.698897	-3.172683	-2.918338
H	3.107109	-3.521683	-1.880028
H	3.610138	2.956417	1.938111
H	3.494666	1.711415	3.223586
H	2.144929	2.845633	2.950058
H	4.750891	-1.941462	-1.453452
H	4.977866	1.126524	1.538777
H	6.097981	-0.509837	0.054074

R = -NH_2

H	-4.867160	1.864079	1.054049
H	-4.857923	-1.898444	-1.024764
H	-6.117448	-0.020638	0.015229
H	-1.298127	1.935331	1.373834
H	-2.779087	2.741248	1.614427
H	-2.765534	-2.764065	-1.587822
H	-1.288269	-1.950856	-1.347253

C	-2.960460	1.017903	0.577417
C	-4.350826	1.032690	0.589838
C	-2.955321	-1.041221	-0.551201
C	-4.345689	-1.063923	-0.561623
C	-5.030863	-0.017537	0.014473
N	-2.247349	-2.075264	-1.065395
N	-2.257432	2.055853	1.090908
N	-2.279991	-0.009608	0.012422
I	0.000343	-0.001028	0.007480
N	2.280697	0.007696	0.001855
N	2.242823	2.098125	-1.026933
N	2.263499	-2.083023	1.030679
C	2.963914	-1.032445	0.539761
C	4.354319	-1.046488	0.546771
C	5.031560	0.017634	-0.005892
C	4.343601	1.076830	-0.554582
C	2.953299	1.052799	-0.539752
H	4.872904	-1.888409	0.988960
H	4.853605	1.922515	-0.999576
H	6.118140	0.021537	-0.008990
H	1.282747	1.979750	-1.307979
H	2.758660	2.799204	-1.535144
H	2.787467	-2.779881	1.536457
H	1.305001	-1.970180	1.319449

R = -CH_3

H	-4.852669	2.131089	-0.023835
H	-4.849744	-2.157632	0.024213
H	-6.125901	-0.014159	-0.001504
H	-2.868426	-3.311033	0.039414
H	-1.539253	-2.549304	-0.849444
H	-1.545531	2.527669	0.858924
H	-2.872936	3.287248	-0.034396
H	-1.541584	-2.529851	0.914766
H	-1.542222	2.507659	-0.905270
C	-2.182741	-2.464440	0.030983
C	-2.186099	2.441616	-0.023516
C	-2.953801	1.161855	-0.010389
C	-4.339245	1.175943	-0.012449
C	-2.952198	-1.185751	0.015811
C	-4.337620	-1.201772	0.014194
C	-5.039314	-0.013402	-0.000057
N	-2.291502	-0.011483	0.003591
I	0.000644	-0.009941	0.006803
N	2.292825	-0.008399	0.010128
C	2.955121	-1.181746	0.024187
C	4.340559	-1.195828	0.026630
C	5.040642	-0.006478	0.014559
C	4.338955	1.181892	0.000191

C	2.953526	1.165856	-0.001848
C	2.184067	2.444546	-0.017259
C	2.187431	-2.461522	0.036966
H	2.874291	-3.307141	0.047529
H	1.542979	2.509787	-0.901106
H	1.543608	-2.527889	0.918727
H	1.546828	-2.547300	-0.845480
H	2.869749	3.291138	-0.025788
H	1.540500	2.529566	0.863097
H	4.853995	-2.150968	0.038054
H	4.851067	2.137759	-0.009596
H	6.127228	-0.005737	0.016337

R = -NMe_2

H	-4.255650	2.433246	-1.156904
H	-4.052507	-1.662000	-2.371243
H	-4.887256	0.629809	-2.712628
H	-2.697890	3.811563	-0.388288
H	-2.240984	4.118763	1.291925
H	-3.913516	3.674846	0.908247
H	-2.118624	0.688139	2.319897
H	-2.840052	2.208255	2.880565
H	-1.118233	2.147266	2.442795
H	-1.827891	-4.213609	-1.162293
H	-2.352706	-3.088494	-2.418746
H	-3.546852	-3.800823	-1.302438
H	-2.487372	-3.566858	1.184419
H	-2.008005	-1.902720	1.565760
H	-0.819767	-3.028606	0.878572
C	-2.114091	1.768751	2.184971
C	-2.861409	3.495709	0.644093
C	-2.525279	-3.404425	-1.387896
C	-1.872134	-2.719718	0.857688
C	-2.873185	1.183210	-0.072226
C	-3.793770	1.459501	-1.079563
C	-2.766161	-1.083955	-0.737507
C	-3.681148	-0.849134	-1.763735
C	-4.157132	0.434339	-1.931258
N	-2.275849	-2.317659	-0.471149
N	-2.471034	2.118134	0.825952
N	-2.305139	-0.051753	0.022616
I	-0.005981	0.015199	0.168564
N	2.296753	0.081249	0.091143
N	2.280021	2.336740	-0.449085
N	2.438642	-2.072485	0.941456
C	2.867977	-1.154694	0.038181
C	3.819622	-1.449560	-0.934453
C	4.209292	-0.440704	-1.793919
C	3.727540	0.845245	-1.666495

C	2.780237	1.098799	-0.674708
C	1.835814	2.765392	0.858397
C	2.555970	3.404908	-1.379982
C	2.836363	-3.452662	0.798835
C	2.039702	-1.697202	2.281789
H	4.284067	-2.424148	-0.978796
H	4.117543	1.646636	-2.277558
H	4.963882	-0.650541	-2.547822
H	2.440491	3.619607	1.186463
H	1.950328	1.963038	1.586718
H	0.783157	3.073663	0.840879
H	1.849367	4.216189	-1.193970
H	2.417617	3.066711	-2.408912
H	3.573127	3.806606	-1.270022
H	2.037984	-0.614160	2.395345
H	2.744806	-2.121145	3.007826
H	1.037100	-2.072992	2.516427
H	2.704643	-3.789208	-0.231546
H	2.197485	-4.064002	1.439830
H	3.880266	-3.624313	1.098147

R = -CH_2F

H	-4.995152	1.735437	0.291868
H	-4.690238	-2.496549	-0.389824
H	-6.118915	-0.467194	-0.087984
H	-2.639888	-3.482639	-0.608522
H	-1.319269	-2.466077	-1.244203
H	-1.704977	2.469318	-0.442340
H	-1.726961	2.161304	1.313152
C	-2.007719	-2.613710	-0.401105
C	-2.350430	2.212278	0.409241
C	-3.039605	0.892272	0.178690
C	-4.424095	0.825900	0.148198
C	-2.873597	-1.400530	-0.199251
C	-4.248346	-1.518062	-0.233762
C	-5.035233	-0.392087	-0.062727
F	-1.269052	-2.870112	0.723099
F	-3.277457	3.189324	0.571766
N	-2.290635	-0.204413	0.002809
I	-0.003229	-0.006442	0.007454
N	2.283695	0.191377	0.011949
F	3.270470	-3.202631	-0.555612
F	1.262563	2.857310	-0.709229
C	3.032575	-0.905415	-0.163841
C	4.417071	-0.839002	-0.134015
C	5.028321	0.379058	0.076149
C	4.241521	1.505091	0.247188
C	2.866762	1.387580	0.213369
C	2.000981	2.600858	0.415128

C	2.343390	-2.225563	-0.393565
H	1.698019	-2.482145	0.458188
H	1.719848	-2.175124	-1.297462
H	2.633218	3.469692	0.622635
H	1.312389	2.453322	1.258078
H	4.988068	-1.748596	-0.277577
H	4.683479	2.483627	0.402758
H	6.112016	0.454159	0.100839

R = F

H	-6.120829	0.000030	0.000384
H	-4.838438	2.119491	0.452803
H	-4.838542	-2.119460	-0.452175
C	-2.975004	-1.114355	-0.237709
C	-4.347152	-1.175339	-0.250582
C	-2.974950	1.114339	0.238117
C	-4.347095	1.175358	0.251149
C	-5.034316	0.000018	0.000322
F	-2.251360	2.174220	0.464583
F	-2.251467	-2.174251	-0.464246
N	-2.287278	-0.000016	0.000165
I	-0.000001	-0.000030	-0.000007
N	2.287278	-0.000024	-0.000187
F	2.251465	-2.174278	0.464136
F	2.251362	2.174229	-0.464525
C	2.975003	-1.114371	0.237654
C	4.347151	-1.175352	0.250546
C	5.034317	0.000017	-0.000301
C	4.347096	1.175365	-0.251095
C	2.974951	1.114342	-0.238087
H	4.838540	-2.119480	0.452108
H	4.838440	2.119505	-0.452707
H	6.120829	0.000032	-0.000345

R = Cl

H	-6.152606	0.000035	0.000039
H	-4.870520	2.155468	-0.000373
H	-4.870582	-2.155440	0.000437
C	-2.990794	-1.150939	0.000217
C	-4.371546	-1.193738	0.000243
C	-2.990757	1.150918	-0.000207
C	-4.371509	1.193753	-0.000203
C	-5.066013	0.000018	0.000025
Cl	-2.124735	2.618868	-0.000442
Cl	-2.124794	-2.618912	0.000516
N	-2.302048	-0.000025	0.000008
I	0.000000	-0.000020	-0.000017
N	2.302047	-0.000025	-0.000019

Cl	2.124793	-2.618912	-0.000580
Cl	2.124734	2.618868	0.000397
C	2.990794	-1.150939	-0.000215
C	4.371546	-1.193738	-0.000230
C	5.066013	0.000018	0.000002
C	4.371509	1.193753	0.000225
C	2.990757	1.150918	0.000218
H	4.870582	-2.155440	-0.000432
H	4.870520	2.155468	0.000394
H	6.152606	0.000035	-0.000004

R = -CHF$_2$

H	-5.106917	1.597384	0.003688
H	-4.663717	-2.680909	-0.006751
H	-6.160761	-0.673868	0.030141
H	-1.217399	-2.651612	-0.832756
H	-1.728281	2.278531	-0.821697
C	-1.967290	-2.709743	-0.027699
C	-2.473051	2.178088	-0.016082
C	-3.128674	0.804449	0.000291
C	-4.505836	0.695340	0.007715
C	-2.890118	-1.499363	-0.005324
C	-4.260350	-1.674813	0.001933
C	-5.080034	-0.561949	0.017198
F	-1.866119	2.402537	1.165725
F	-2.693025	-3.816452	-0.192262
F	-1.327703	-2.811287	1.153856
F	-3.410121	3.113640	-0.174873
N	-2.338684	-0.277985	-0.000979
I	-0.044697	-0.041575	0.038610
N	2.249426	0.194764	0.078143
F	3.320797	-3.196831	0.252795
F	1.238126	2.728043	-1.076668
F	1.776671	-2.486181	-1.087868
F	2.603877	3.733230	0.268955
C	3.039382	-0.887701	0.076932
C	4.416545	-0.778632	0.069222
C	4.990786	0.478642	0.059399
C	4.171140	1.591530	0.074645
C	2.800906	1.416105	0.082188
C	1.878063	2.626522	0.104666
C	2.383751	-2.261315	0.093807
H	1.639057	-2.361455	0.899536
H	1.128471	2.568399	0.909959
H	5.017602	-1.680692	0.073261
H	4.574532	2.597618	0.083096
H	6.071514	0.590523	0.046203

R = Br

H	-6.157218	-0.000795	0.000057
H	-4.878285	2.151920	0.028673
H	-4.876890	-2.152665	-0.028663
C	-2.993176	-1.150830	-0.015269
C	-4.374395	-1.192944	-0.015944
C	-2.993923	1.151281	0.015299
C	-4.375174	1.192523	0.015793
C	-5.070502	-0.000437	-0.000028
Br	-2.078308	2.774905	0.036839
Br	-2.076571	-2.773902	-0.037479
N	-2.304395	0.000454	-0.000042
I	0.000011	0.001021	0.000017
N	2.304396	0.000442	0.000047
Br	2.076577	-2.773917	0.037259
Br	2.078298	2.774896	-0.036923
C	2.993180	-1.150840	0.015282
C	4.374399	-1.192947	0.015913
C	5.070499	-0.000437	0.000058
C	4.375168	1.192522	-0.015765
C	2.993917	1.151276	-0.015232
H	4.876899	-2.152666	0.028657
H	4.878277	2.151921	-0.028576
H	6.157216	-0.000789	0.000027

R = I

H	-6.170487	-0.018065	0.005642
H	-4.900501	2.091891	0.419077
H	-4.889089	-2.121114	-0.407781
C	-2.998243	-1.143825	-0.215487
C	-4.382236	-1.181406	-0.223477
C	-3.004357	1.125063	0.225479
C	-4.388560	1.155053	0.234256
C	-5.083638	-0.015077	0.005452
I	-2.044907	2.931407	0.575783
I	-2.028952	-2.945110	-0.564873
N	-2.314569	-0.007448	0.004603
I	-0.000013	-0.000063	0.000691
N	2.314580	0.007279	-0.003268
I	2.046253	-2.929819	0.576009
I	2.027567	2.943797	-0.579397
C	3.005095	-1.124559	0.218604
C	4.389278	-1.154467	0.223650
C	5.083642	0.015109	-0.010124
C	4.381529	1.180793	-0.240160
C	2.997521	1.143168	-0.228190
H	4.901763	-2.090806	0.409510
H	4.887833	2.120033	-0.428294
H	6.170487	0.018162	-0.012906

R = CN

H	-6.139125	-0.009019	-0.018066
H	-4.846283	-1.537202	-1.531643
H	-4.859117	1.522719	1.502792
C	-2.216457	-1.691812	-1.681192
C	-2.230659	1.684562	1.667292
C	-2.961035	-0.825091	-0.822739
C	-4.348842	-0.852681	-0.852124
C	-2.967920	0.815790	0.804616
C	-4.355935	0.839570	0.826126
C	-5.052713	-0.007518	-0.014981
N	-1.664740	-2.406314	-2.388889
N	-1.685133	2.400556	2.378270
N	-2.282478	-0.003714	-0.007126
I	0.000432	-0.000500	-0.000445
N	2.283437	0.003042	0.006465
N	1.672335	-2.381133	2.408431
N	1.679481	2.385083	-2.399452
C	2.964211	-0.809338	0.829193
C	4.352091	-0.832437	0.859268
C	5.053661	0.007803	0.015278
C	4.354589	0.845616	-0.833193
C	2.966644	0.817747	-0.811956
C	2.222005	-1.670999	1.694761
C	2.227004	1.676852	-1.682241
H	4.855921	1.524521	-1.515478
H	6.140072	0.009699	0.018757
H	4.851385	-1.509620	1.544760

R = -CF_3

H	-4.955919	1.991947	0.460644
H	-4.867660	-2.220471	-0.349288
H	-6.195436	-0.141459	0.057548
C	-2.213199	-2.494686	-0.421917
C	-2.315669	2.380225	0.519254
C	-3.048075	1.057211	0.265276
C	-4.429825	1.062848	0.276906
C	-3.000768	-1.203572	-0.169462
C	-4.381027	-1.269003	-0.171275
C	-5.109344	-0.118094	0.054272
F	-3.046349	-3.502916	-0.595489
F	-1.431921	-2.787699	0.605247
F	-1.466194	-2.395356	-1.509453
F	-1.544242	2.301496	1.591214
F	-3.190827	3.346629	0.721941
F	-1.568587	2.722553	-0.518122
N	-2.336965	-0.058084	0.044095

I	-0.000420	-0.002003	0.013409
N	2.336334	0.057744	-0.026890
F	1.558062	-2.714826	-0.608804
F	1.593299	-2.326815	1.506707
F	3.216331	-3.354458	0.574661
F	3.019599	3.512867	-0.639397
F	1.442275	2.778169	0.598222
F	1.415654	2.413819	-1.521594
C	3.056053	-1.058678	0.157480
C	4.437562	-1.060871	0.129348
C	5.107716	0.124970	-0.095636
C	4.370593	1.276991	-0.283780
C	2.991092	1.207930	-0.243145
C	2.193843	2.500452	-0.455047
C	2.333917	-2.387111	0.412078
H	4.970794	-1.991139	0.284446
H	4.849789	2.232135	-0.462289
H	6.193394	0.151220	-0.123276

R = -NO_2

H	-6.132850	-0.126664	-0.476721
H	-4.914959	2.069100	-0.180504
H	-4.800147	-2.257106	-0.198691
C	-3.013053	-1.174033	0.199610
C	-4.354165	-1.273360	-0.095828
C	-3.072833	1.078923	0.209214
C	-4.417387	1.109543	-0.085793
C	-5.071522	-0.099446	-0.245508
O	-2.627492	-3.377706	-0.224581
O	-1.411782	-2.403533	1.279026
O	-2.802764	3.302987	-0.197258
O	-1.541356	2.382431	1.302584
N	-2.397718	2.370117	0.451130
N	-2.270224	-2.429764	0.430007
N	-2.353495	-0.029825	0.343553
I	-0.021937	0.034967	-0.005707
N	2.309372	0.100039	-0.355006
N	2.354145	-2.299923	-0.462066
N	2.225494	2.499895	-0.442155
O	1.497596	-2.312576	-1.313316
O	2.759616	-3.232597	0.186339
O	2.582197	3.447986	0.212520
O	1.367338	2.473278	-1.291464
C	2.968660	1.244443	-0.211446
C	4.309811	1.344180	0.083669
C	5.027485	0.170484	0.233492
C	4.373605	-1.038719	0.074229
C	3.028990	-1.008508	-0.220494
H	6.088859	0.198016	0.464458

H	4.871436	-1.998130	0.169057
H	4.755560	2.328068	0.186201

Table S5. $[(3\text{-R-py})_2I]^+$ adducts.

R = -NH$_2$

H	-2.473634	1.314314	1.007254
H	-6.027452	-0.735259	-0.294876
H	-4.564638	-2.240552	-1.614763
H	-2.082132	-1.885785	-1.563677
H	-6.116032	1.100171	1.334802
H	-4.708752	1.915403	1.852512
C	-2.767632	-1.259166	-1.003315
C	-4.138885	-1.439217	-1.020194
C	-2.991419	0.541784	0.444416
C	-4.383478	0.426259	0.476121
C	-4.948656	-0.603603	-0.286003
N	-5.141529	1.302115	1.181464
N	-2.236594	-0.280299	-0.275597
I	-0.000992	-0.000531	-0.271968
N	2.234549	0.282571	-0.276645
N	5.133202	-1.195503	1.298902
C	2.990016	-0.520863	0.463513
C	4.380896	-0.395259	0.502292
C	4.945013	0.619016	-0.281057
C	4.134897	1.434236	-1.037506
C	2.764364	1.248799	-1.021930
H	6.132382	-1.214144	1.173923
H	4.727745	-2.024460	1.702035
H	2.473081	-1.281841	1.042640
H	2.078495	1.860168	-1.598508
H	4.559835	2.222917	-1.649326
H	6.022964	0.757723	-0.285157

R = -NMe$_2$

H	-1.482098	2.707425	0.240671
H	-5.718312	2.026447	0.225579
H	-3.828716	3.597627	0.260250
H	-2.821124	-1.180202	0.155695
H	-6.088276	-2.630521	0.122964
H	-4.587483	-2.373038	1.019171
H	-4.586530	-2.333990	-0.759456
H	-7.074066	0.407951	-0.700561
H	-7.074966	0.369805	1.077274
H	-7.495605	-1.079002	0.156702
C	-3.116419	-0.137530	0.178374
C	-4.465635	0.257759	0.186891
C	-2.349584	2.056718	0.226427

C	-3.649194	2.527927	0.236738
C	-4.703853	1.645375	0.217292
C	-5.160402	-2.061495	0.135966
C	-6.840396	-0.209842	0.175636
N	-5.466654	-0.652825	0.166811
N	-2.125496	0.746360	0.197742
I	-0.000470	-0.000251	0.181428
N	2.124561	-0.746841	0.165129
N	5.465689	0.652234	0.203894
C	5.159415	2.060806	0.238758
C	2.348681	-2.057103	0.132568
C	3.648302	-2.528281	0.122243
C	4.702940	-1.645798	0.145587
C	4.464686	-0.258281	0.179993
C	3.115461	0.136982	0.188346
C	6.839434	0.209259	0.195220
H	3.827845	-3.597906	0.095638
H	1.481214	-2.707758	0.115264
H	2.820138	1.179577	0.214036
H	5.717408	-2.026842	0.137283
H	4.587500	2.375085	-0.644125
H	6.087278	2.629780	0.254589
H	4.584529	2.330536	1.134371
H	7.074914	-0.367788	-0.707844
H	7.072207	-0.411065	1.069872
H	7.494632	1.078352	0.217329

R = -OMe

H	-2.136933	1.499401	-1.947996
H	-6.039263	0.723905	-0.322433
H	-4.612124	1.839611	-2.015095
H	-2.458557	-0.968576	1.356063
H	-6.677439	-1.390500	2.566891
H	-6.862908	-1.166510	0.807235
H	-6.697242	0.257096	1.884393
C	-2.969799	-0.351620	0.621989
C	-4.357537	-0.215190	0.657134
C	-2.802740	1.032393	-1.229640
C	-4.168438	1.209058	-1.251815
C	-4.963841	0.585390	-0.305755
C	-6.383932	-0.779553	1.714219
O	-4.977623	-0.877743	1.627611
N	-2.238806	0.259754	-0.297728
I	-0.000264	-0.009935	-0.287579
N	2.238360	-0.279124	-0.280238
O	4.970098	0.929885	1.611228
C	6.376311	0.837124	1.705105
C	2.805859	-1.084308	-1.181936
C	4.171761	-1.260398	-1.193420

C	4.963649	-0.602270	-0.267992
C	4.353655	0.231966	0.663490
C	2.965917	0.365695	0.619128
H	4.618355	-1.917610	-1.932132
H	2.142707	-1.577570	-1.885036
H	2.451787	1.008252	1.328821
H	6.039235	-0.740288	-0.276250
H	6.666573	1.479167	2.535754
H	6.857182	1.191632	0.785934
H	6.690711	-0.192153	1.913660

R = -OH

H	-2.090270	1.884840	-1.428308
H	-6.035044	0.746090	-0.143023
H	-4.565090	2.248560	-1.478268
H	-2.499456	-1.318037	1.158083
H	-5.998426	-1.115246	1.334344
C	-2.993097	-0.538252	0.584849
C	-4.377296	-0.403580	0.610761
C	-2.775299	1.259421	-0.864982
C	-4.142151	1.447005	-0.882093
C	-4.955826	0.612245	-0.140634
O	-5.049187	-1.272235	1.365500
N	-2.237995	0.279676	-0.138103
I	-0.000281	0.000807	-0.129840
N	2.237403	-0.278298	-0.120135
O	5.037515	1.274988	1.402599
C	2.779937	-1.259344	-0.841366
C	4.146814	-1.447406	-0.847708
C	4.955046	-0.611776	-0.101302
C	4.371114	0.405383	0.644089
C	2.987201	0.540485	0.607341
H	4.574038	-2.250024	-1.439374
H	2.099039	-1.885409	-1.408966
H	2.489424	1.321324	1.175548
H	6.034205	-0.745980	-0.095158
H	5.986900	1.117578	1.378993

R = -CH_3

H	-2.078464	1.344837	-1.899982
H	-6.021823	0.048757	-0.793606
H	-4.548216	1.394306	-2.286942
H	-2.497955	-1.240153	1.282304
H	-5.799095	-2.281814	0.737918
H	-5.945914	-0.908996	1.830394
H	-4.617683	-2.059421	2.035772
C	-3.009087	-0.674188	0.506532
C	-4.387112	-0.713397	0.375423

C	-2.768184	0.777754	-1.280962
C	-4.133507	0.795803	-1.482920
C	-4.943405	0.047142	-0.650536
C	-5.226780	-1.536665	1.297162
N	-2.234507	0.051617	-0.301275
I	0.000052	0.050542	-0.001689
N	2.234615	0.053694	0.297902
C	5.226569	-1.550376	-1.285294
C	2.768417	0.788926	1.270713
C	4.133733	0.808529	1.472576
C	4.943473	0.051807	0.647367
C	4.387050	-0.718254	-0.371397
C	3.009044	-0.679911	-0.502971
H	4.548559	1.414519	2.270909
H	2.078801	1.362000	1.884307
H	2.497788	-1.253111	-1.273331
H	6.021880	0.054456	0.790501
H	4.617245	-2.081255	-2.017897
H	5.800037	-2.289169	-0.718867
H	5.944673	-0.927658	-1.825685

R = -CH_2F

H	-2.265335	1.532697	1.011093
H	-6.073179	0.201963	-0.455124
H	-4.853571	-1.352159	-1.976880
H	-2.355154	-1.376002	-1.916991
H	-5.569414	2.655844	0.771612
H	-5.603895	1.395520	2.028664
C	-2.937048	-0.719000	-1.276275
C	-4.317397	-0.696322	-1.299605
C	-2.884129	0.913905	0.367586
C	-4.263867	0.992520	0.400659
C	-4.985500	0.167557	-0.453256
C	-4.951552	1.944054	1.335691
F	-4.033303	2.641571	2.060344
N	-2.252837	0.074869	-0.455456
I	0.001747	0.003210	-0.448633
N	2.256205	-0.068616	-0.440686
F	4.017108	-2.713335	2.007205
C	4.940942	-1.993258	1.312320
C	2.881080	-0.932815	0.360959
C	4.260505	-1.013003	0.401994
C	4.988715	-0.162520	-0.420737
C	4.327218	0.727208	-1.245214
C	2.946731	0.749739	-1.231621
H	2.257288	-1.570947	0.980300
H	2.369794	1.426347	-1.856209
H	4.868618	1.403359	-1.897905
H	6.076370	-0.197338	-0.415455

H	5.563653	-2.686915	0.731261
H	5.587414	-1.466645	2.027457

R = -F

H	-2.327041	1.451358	1.464379
H	-6.093636	0.001800	0.010278
H	-4.812890	-1.515286	-1.542011
H	-2.317770	-1.450165	-1.490351
C	-2.918759	-0.818003	-0.843315
C	-4.299513	-0.844095	-0.861736
C	-2.907422	0.806339	0.810525
C	-4.287653	0.814218	0.826546
C	-5.007794	-0.013943	-0.012344
F	-4.903463	1.625138	1.655761
N	-2.257019	-0.005562	-0.020012
I	0.000002	0.000023	-0.022507
N	2.257034	0.005684	-0.018414
F	4.902670	-1.661978	1.621884
C	2.907032	-0.824700	0.793964
C	4.287255	-0.832649	0.810776
C	5.007796	0.014410	-0.008677
C	4.299927	0.863425	-0.839565
C	2.919167	0.836688	-0.822653
H	2.326336	-1.484579	1.432533
H	2.318486	1.483399	-1.455441
H	4.813629	1.550080	-1.503976
H	6.093626	-0.001612	0.014356

R = -CHF$_2$

H	-2.304535	1.787924	-0.771022
H	-6.085013	0.089537	0.345627
H	-4.825972	-1.782168	1.437161
H	-2.331135	-1.747433	1.358941
H	-5.356707	1.949298	-1.998931
C	-2.928118	-0.966304	0.895359
C	-4.307469	-0.975096	0.930993
C	-2.908796	1.014792	-0.303454
C	-4.288117	1.069509	-0.306505
C	-4.998362	0.057634	0.322653
C	-5.003732	2.212701	-0.988713
F	-6.064324	2.574933	-0.252469
F	-4.178723	3.267027	-1.091374
N	-2.257787	0.013885	0.289139
I	-0.001096	-0.003928	0.285341
N	2.255612	-0.021812	0.286817
F	6.062212	-2.582967	-0.256937
F	4.175060	-3.277200	-1.090594
C	2.905917	-1.023389	-0.305391

C	4.285236	-1.078360	-0.309637
C	4.996221	-0.066013	0.317900
C	4.306041	0.967448	0.925836
C	2.926665	0.958868	0.891462
C	4.999895	-2.222379	-0.991466
H	5.350544	-1.960543	-2.002901
H	2.301096	-1.796901	-0.771612
H	2.330238	1.740551	1.354826
H	4.825145	1.774921	1.430746
H	6.082883	-0.098107	0.339948

R = -Cl

H	-2.321235	1.494773	1.380669
H	-6.087860	-0.026115	-0.013360
H	-4.809216	-1.587194	-1.499480
H	-2.311288	-1.512462	-1.454837
C	-2.915254	-0.858484	-0.832387
C	-4.294853	-0.889424	-0.847217
C	-2.911789	0.828009	0.758053
C	-4.295813	0.843966	0.787563
C	-5.001684	-0.028567	-0.027708
Cl	-5.098148	1.935570	1.825876
N	-2.257298	-0.011579	-0.040410
I	0.000083	0.001157	-0.043502
N	2.257317	0.012988	-0.042953
Cl	5.095020	-1.850236	1.909534
C	2.910853	-0.789430	0.793625
C	4.294932	-0.807358	0.822782
C	5.001777	0.024266	-0.033541
C	4.296011	0.847297	-0.891868
C	2.916357	0.820228	-0.874441
H	2.319635	-1.424649	1.447775
H	2.313243	1.445253	-1.526745
H	4.811240	1.512159	-1.576991
H	6.087971	0.019823	-0.020438

R = -Br

H	-2.328677	1.743795	1.034715
H	-6.087518	-0.057300	-0.015707
H	-4.797884	-1.888346	-1.131785
H	-2.300173	-1.792356	-1.100401
C	-2.909107	-1.025070	-0.630863
C	-4.288310	-1.065722	-0.641219
C	-2.918458	0.960312	0.566595
C	-4.302045	0.975995	0.588571
C	-5.001281	-0.053227	-0.024297
Br	-5.185001	2.376337	1.439893
N	-2.257294	-0.026413	-0.034914

I	-0.000032	-0.000762	-0.038156
N	2.257244	0.025089	-0.039155
Br	5.187827	-2.329814	1.505556
C	2.919534	-0.941965	0.592298
C	4.303160	-0.956812	0.612404
C	5.001208	0.052568	-0.033885
C	4.287079	1.044864	-0.681485
C	2.907905	1.004364	-0.667632
H	2.330622	-1.710252	1.086010
H	2.298060	1.756170	-1.160454
H	4.795675	1.851553	-1.198804
H	6.087467	0.056982	-0.027286

R = -I

H	-2.341450	2.024651	-0.036447
H	-6.084676	-0.096151	0.004215
H	-4.777369	-2.221349	0.044268
H	-2.280378	-2.100688	0.041386
C	-2.897511	-1.206844	0.024611
C	-4.275972	-1.259356	0.025934
C	-2.929875	1.111051	-0.019137
C	-4.314629	1.129566	-0.019145
C	-4.998365	-0.079096	0.003732
I	-5.312448	2.938293	-0.052668
N	-2.256578	-0.038199	0.002290
I	0.000006	0.000031	0.000248
N	2.256576	0.038274	-0.001859
I	5.312331	-2.938314	0.053651
C	2.929828	-1.110996	0.020250
C	4.314584	-1.129586	0.019706
C	4.998366	0.079033	-0.004153
C	4.276023	1.259313	-0.026827
C	2.897561	1.206870	-0.025121
H	2.341375	-2.024559	0.038410
H	2.280460	2.100727	-0.042394
H	4.777462	2.221270	-0.045890
H	6.084678	0.096049	-0.005061

R = -CF_3

H	-2.161625	-2.281916	1.665942
H	-6.051490	-0.713982	0.712276
H	-4.632612	-2.320966	2.015026
H	-2.452402	0.761706	-1.108670
C	-5.182042	1.123518	-1.085533
C	-2.981132	0.099080	-0.428531
C	-4.351300	0.138247	-0.289855
C	-2.823904	-1.608412	1.127744
C	-4.192181	-1.621056	1.313239

C	-4.970360	-0.733575	0.593595
F	-6.097910	0.488356	-1.800877
F	-4.427772	1.832987	-1.910760
F	-5.812194	1.961120	-0.275626
N	-2.245824	-0.763554	0.274108
I	-0.003257	-0.777666	0.012639
N	2.239210	-0.780534	-0.250062
F	4.430244	1.907256	1.811762
F	5.813776	1.957000	0.171675
F	6.096311	0.554060	1.762018
C	5.181764	1.158879	1.019122
C	2.814243	-1.665285	-1.064472
C	4.182257	-1.689969	-1.250627
C	4.963528	-0.772356	-0.573406
C	4.347625	0.140759	0.269656
C	2.977505	0.111610	0.411338
H	4.620113	-2.422629	-1.919831
H	2.149696	-2.361186	-1.570437
H	2.451254	0.806277	1.060732
H	6.044571	-0.761032	-0.693913

R = -SO_3H

H	-1.508518	2.576047	1.191917
H	-5.684708	2.160460	0.223178
H	-3.804183	3.548829	1.162724
H	-2.820137	-0.944731	-0.537487
H	-7.012578	-1.011268	0.644751
C	-3.081283	0.037585	-0.149458
C	-4.380856	0.503966	-0.181496
C	-2.358623	2.017354	0.808309
C	-3.633494	2.544564	0.789924
C	-4.668271	1.777395	0.281504
O	-4.977137	-1.447306	-1.718371
O	-6.687681	0.316901	-1.318868
O	-6.137884	-1.320102	0.369167
S	-5.639624	-0.526657	-0.883152
N	-2.101511	0.792240	0.342577
I	0.015086	0.004050	0.341774
N	2.134818	-0.775524	0.328907
S	5.704857	0.699819	-0.586318
O	6.917923	0.245167	-0.019192
O	5.189392	1.996768	-0.395673
O	5.760482	0.452078	-2.129107
C	2.395396	-2.021721	0.732798
C	3.681851	-2.519528	0.756194
C	4.723263	-1.704474	0.344493
C	4.427830	-0.420882	-0.083609
C	3.120260	0.023931	-0.072374
H	3.859198	-3.531980	1.102780

H	1.541059	-2.615144	1.048687
H	2.861575	1.036726	-0.374271
H	5.756163	-2.044747	0.369424
H	6.555313	-0.045068	-2.368890

R = -CN

H	-2.751343	1.437275	-0.000368
H	-5.912550	-1.478745	-0.003083
H	-4.148370	-3.259726	-0.005308
H	-1.750296	-2.569332	-0.004919
C	-2.554441	-1.838092	-0.003997
C	-3.883992	-2.207734	-0.004196
C	-3.106260	0.409697	-0.001444
C	-4.462612	0.111534	-0.001560
C	-4.855954	-1.225148	-0.002968
C	-5.412741	1.173675	-0.000238
N	-6.170943	2.034436	0.000872
N	-2.191028	-0.553316	-0.002645
I	-0.000168	-0.000114	-0.002336
N	2.190832	0.553210	-0.002020
N	6.171174	-2.033846	-0.005231
C	5.412819	-1.173219	-0.004233
C	3.106242	-0.409621	-0.003162
C	4.462536	-0.111209	-0.002985
C	4.855652	1.225518	-0.001578
C	3.883510	2.207934	-0.000414
C	2.554029	1.838046	-0.000673
H	2.751518	-1.437270	-0.004238
H	1.749778	2.569175	0.000198
H	4.147690	3.259975	0.000693
H	5.912201	1.479302	-0.001412

R = -NO_2

H	-1.629511	2.646210	0.013624
H	-5.849850	1.741508	0.002445
H	-3.986503	3.457998	0.018022
H	-2.845342	-1.315299	-0.019108
C	-3.125971	-0.265539	-0.011015
C	-4.446544	0.126688	-0.009140
C	-2.468705	1.955075	0.007343
C	-3.777067	2.393731	0.009658
C	-4.800109	1.460464	0.001199
O	-5.099529	-2.060243	-0.026628
O	-6.628932	-0.533301	-0.017176
N	-5.482359	-0.911574	-0.018447
N	-2.164782	0.653635	-0.002788
I	-0.003616	-0.007400	-0.006153
N	2.157568	-0.668493	-0.009525

N	5.475251	0.896489	0.005951
O	6.621801	0.518141	0.004740
O	5.092501	2.045185	0.013990
C	2.461402	-1.969961	-0.019570
C	3.769731	-2.408704	-0.021886
C	4.792836	-1.475503	-0.013518
C	4.439368	-0.141705	-0.003269
C	3.118813	0.250612	-0.001395
H	3.979104	-3.472984	-0.030179
H	1.622153	-2.661030	-0.025779
H	2.838259	1.300394	0.006621
H	5.842558	-1.756621	-0.014764

Table S6. $[(3,5\text{-}R_2\text{-py})_2I]^+$ adducts.

R = -NMe$_2$

H	-2.225121	1.513802	-1.469388
H	-6.048077	0.127799	-0.023888
H	-2.278406	-1.414489	1.402074
H	-4.931001	-3.097498	3.109814
H	-3.581634	-1.962272	3.219293
H	-3.603205	-3.207020	1.950056
H	-6.834505	-1.866721	0.714560
H	-6.798186	-0.627195	1.987833
H	-6.751330	-2.342091	2.414904
H	-3.507637	2.117869	-3.275599
H	-3.485241	3.364440	-2.008175
H	-4.815877	3.299888	-3.168626
H	-6.748063	2.164983	-0.752561
H	-6.783618	0.918822	-2.019356
H	-6.661635	2.627112	-2.456671
C	-2.891156	-0.771368	0.784283
C	-4.298011	-0.779604	0.823203
C	-2.860834	0.894974	-0.849968
C	-4.266459	0.960028	-0.882765
C	-4.968056	0.104860	-0.027457
C	-4.229003	-2.509121	2.521303
C	-6.403415	-1.607597	1.690386
C	-6.340329	1.881096	-1.731640
C	-4.135318	2.688236	-2.578665
N	-4.900919	1.823682	-1.715952
N	-4.963149	-1.617808	1.658512
N	-2.249901	0.049373	-0.033936
I	0.000979	0.008137	-0.029105
N	2.251831	-0.034049	-0.016469
N	4.952812	1.602400	1.724936
N	4.914801	-1.783737	-1.705419
C	4.212615	2.481543	2.595019
C	6.392666	1.587787	1.770602

C	4.155361	-2.635538	-2.586069
C	6.354170	-1.844258	-1.708518
C	2.868615	-0.867116	-0.840954
C	4.274383	-0.933188	-0.863336
C	4.969790	-0.092926	0.011552
C	4.293673	0.778649	0.870698
C	2.887172	0.772778	0.819995
H	2.237423	-1.475218	-1.475434
H	2.269986	1.406234	1.443338
H	6.049711	-0.117362	0.023739
H	3.591262	3.187625	2.029063
H	4.910470	3.061390	3.196775
H	3.559954	1.925213	3.280526
H	6.834028	1.862960	0.803801
H	6.781938	0.601194	2.054482
H	6.735325	2.308323	2.511458
H	6.802068	-0.877966	-1.974689
H	6.680725	-2.577667	-2.443997
H	6.752162	-2.146650	-0.730929
H	3.501260	-3.320036	-2.030219
H	4.840103	-3.238405	-3.180222
H	3.532656	-2.054896	-3.278935

R = -NH$_2$

H	-2.296309	1.460539	1.422685
H	-6.059857	-0.095888	0.020158
H	-2.261474	-1.532253	-1.416313
H	-5.934439	1.557499	1.812198
H	-4.447199	2.170576	2.365419
H	-4.397301	-2.309023	-2.340647
H	-5.897916	-1.743081	-1.774249
C	-2.875839	-0.887510	-0.796217
C	-4.274835	-0.944181	-0.819477
C	-2.895410	0.796695	0.807851
C	-4.295247	0.809124	0.843632
C	-4.972981	-0.078494	0.015199
N	-4.911779	-1.856607	-1.602146
N	-4.953614	1.700762	1.632288
N	-2.250950	-0.035394	0.003055
I	0.000612	-0.000957	-0.004178
N	2.252216	0.033038	-0.008955
N	4.960276	-1.542457	1.768163
N	4.907794	1.689447	-1.791497
C	2.899916	-0.758874	0.832931
C	4.299813	-0.767296	0.866032
C	4.974296	0.074088	-0.011760
C	4.272863	0.894701	-0.888223
C	2.873899	0.844094	-0.852214
H	5.946112	-1.704323	1.637974

H	4.461382	-2.286452	2.228994
H	2.303222	-1.385014	1.488326
H	2.257129	1.451957	-1.506241
H	6.061197	0.090611	-0.012824
H	5.888717	1.880429	-1.663926
H	4.385807	2.417542	-2.252076

R = -OMe

H	-1.984361	2.011156	-1.060840
H	-5.995137	1.170460	0.241900
H	-2.590518	-1.133127	1.553842
H	-6.882589	-1.515558	2.536605
H	-6.982545	-0.868620	0.877024
H	-6.763055	0.243612	2.264974
H	-4.465657	4.254346	-2.469168
H	-3.289275	2.947373	-2.763937
H	-3.071625	4.060295	-1.374704
C	-3.036689	-0.327906	0.978704
C	-4.417422	-0.099211	1.012260
C	-2.713299	1.446212	-0.493730
C	-4.073997	1.723416	-0.502553
C	-4.936477	0.938055	0.262128
C	-6.514655	-0.744664	1.860669
C	-3.806423	3.530017	-1.992217
O	-4.634659	2.705715	-1.202129
O	-5.116605	-0.924768	1.784006
N	-2.249254	0.433465	0.244971
I	-0.036885	0.010733	0.234933
N	2.177547	-0.417845	0.230304
O	4.996871	0.567340	2.109523
O	4.416669	-2.403688	-1.638690
C	6.389171	0.361365	2.199637
C	5.782107	-2.747274	-1.720013
C	2.665783	-1.257927	-0.671818
C	4.027568	-1.549788	-0.696322
C	4.867775	-0.949995	0.235737
C	4.315653	-0.074610	1.164833
C	2.945442	0.173818	1.134895
H	1.982487	-1.708869	-1.383504
H	2.482260	0.849742	1.845770
H	5.929304	-1.160300	0.238050
H	6.732600	0.976021	3.030919
H	6.896655	0.678367	1.280785
H	6.621339	-0.690094	2.406517
H	5.868541	-3.448808	-2.548943
H	6.123931	-3.232183	-0.797902
H	6.400792	-1.865618	-1.925494

R = -CH_3

H	-2.321391	1.705637	-1.086709
H	-6.064299	-0.041343	0.060825
H	-2.288629	-1.765649	1.132752
H	-5.636148	-2.721653	0.717209
H	-5.637728	-1.769161	2.197867
H	-4.292436	-2.866331	1.858212
H	-4.346100	2.802912	-1.758009
H	-5.681096	2.628386	-0.610666
H	-5.676049	1.687933	-2.099012
C	-2.902571	-1.007641	0.652172
C	-4.286703	-1.058146	0.694945
C	-2.920980	0.943726	-0.594322
C	-4.305974	0.985687	-0.609531
C	-4.975003	-0.038230	0.049612
C	-5.035696	2.087499	-1.307939
C	-4.995485	-2.165110	1.406615
N	-2.254823	-0.029930	0.022408
I	-0.000882	-0.023018	0.000144
N	2.253194	-0.016691	-0.022132
C	2.913954	0.948312	0.613681
C	4.298722	0.997266	0.630398
C	4.973384	-0.010032	-0.048295
C	4.290738	-1.020663	-0.713916
C	2.906349	-0.978481	-0.670565
C	5.005580	-2.109076	-1.447793
C	5.022208	2.089238	1.350452
H	2.310178	1.697222	1.120694
H	2.296608	-1.730460	-1.165744
H	6.062690	-0.007388	-0.058758
H	4.306480	-2.811871	-1.903071
H	5.659368	-2.667731	-0.772663
H	5.635419	-1.694184	-2.239391
H	5.670817	1.677354	2.128324
H	4.328505	2.787397	1.820860
H	5.658469	2.652401	0.662438

R = -OH

H	-2.352189	1.483914	1.440998
H	-6.122891	-0.025777	0.033019
H	-2.344874	-1.509404	-1.420334
H	-5.848979	-1.735521	-1.603285
H	-4.489544	2.218014	2.160118
C	-2.934748	-0.853879	-0.788088
C	-4.329438	-0.890152	-0.809489
C	-2.951150	0.826363	0.817753
C	-4.340048	0.837611	0.841094
C	-5.036382	-0.034218	0.014763
O	-5.050998	1.649643	1.623320

O	-4.887614	-1.767281	-1.641985
N	-2.302230	-0.010848	0.011704
I	-0.047562	0.004360	0.006144
N	2.207046	0.014956	-0.003185
O	4.791485	2.007852	-1.369317
O	4.819177	-1.967932	1.324056
C	2.852294	-0.946403	0.644237
C	4.243622	-0.971540	0.652502
C	4.938877	0.020183	-0.023677
C	4.229759	1.009247	-0.689275
C	2.838862	0.978741	-0.660235
H	2.271837	-1.703711	1.160310
H	2.247840	1.732589	-1.169280
H	6.026837	0.022081	-0.032124
H	5.752625	1.963562	-1.347732
H	5.779708	-1.922175	1.285168

R = -CH_2F

H	-2.295973	-2.011581	0.326252
H	-6.068255	-0.032133	-0.171465
H	-2.310007	1.997956	-0.648367
H	-5.662918	2.713796	0.075406
H	-5.641483	2.301094	-1.656091
H	-4.730823	-2.830272	1.425166
H	-4.729623	-3.245402	-0.306460
C	-5.001067	-2.470252	0.423116
C	-5.014147	2.427399	-0.763336
C	-2.906958	1.116373	-0.436181
C	-4.287462	1.152275	-0.450554
C	-2.904371	-1.135494	0.110295
C	-4.289090	-1.182861	0.114881
C	-4.980505	-0.018445	-0.170162
F	-4.125273	3.434144	-0.992460
F	-6.346859	-2.281721	0.376899
N	-2.249684	-0.011505	-0.159382
I	0.005875	0.000462	-0.156177
N	2.261316	0.012387	-0.156775
F	4.137292	-3.434134	-0.985176
F	6.358195	2.282796	0.380936
C	5.012372	2.471447	0.425904
C	2.918753	-1.115801	-0.431983
C	4.299258	-1.151817	-0.445091
C	4.992147	0.019103	-0.165157
C	4.300578	1.183836	0.118213
C	2.915852	1.136571	0.112436
C	5.026087	-2.427319	-0.756041
H	5.673974	-2.713081	0.083600
H	5.654330	-2.301879	-1.648278
H	4.741340	2.832343	1.427427

H	4.741570	3.245999	-0.304547
H	2.321903	-1.997516	-0.643898
H	2.307332	2.012910	0.327034
H	6.079899	0.032679	-0.165451

R = -F

H	-2.328293	1.474295	1.464888
H	-6.099839	0.000604	-0.005851
H	-2.325866	-1.474085	-1.469381
C	-2.905256	-0.823034	-0.822087
C	-4.288057	-0.831666	-0.832203
C	-2.906615	0.823386	0.816497
C	-4.289425	0.832380	0.823956
C	-5.013996	0.000450	-0.004817
F	-4.909318	1.651182	1.638194
F	-4.906590	-1.650303	-1.647636
N	-2.258397	0.000091	-0.002173
I	0.000155	-0.000256	0.000022
N	2.258388	-0.000401	0.002174
F	4.906472	-1.641741	1.656813
F	4.909400	1.641337	-1.647387
C	2.905197	-0.818977	0.826685
C	4.287996	-0.827628	0.836838
C	5.013984	-0.000165	0.004841
C	4.289466	0.827150	-0.828587
C	2.906658	0.818263	-0.821112
H	2.325750	-1.466328	1.477626
H	2.328361	1.465492	-1.473195
H	6.099828	-0.000057	0.005896

R = -CHF$_2$

H	-2.312151	-2.073049	-0.225148
H	-6.079647	-0.016288	-0.069744
H	-2.323165	2.059661	-0.236283
H	-5.400192	2.803367	-1.145332
H	-5.384906	-2.837928	-1.130636
C	-5.013875	-2.535283	-0.137932
C	-5.027279	2.507978	-0.151154
C	-2.917554	1.149643	-0.227498
C	-4.297188	1.183513	-0.176013
C	-2.911384	-1.166179	-0.221304
C	-4.290834	-1.207104	-0.169677
C	-4.993752	-0.013576	-0.143331
F	-4.184057	-3.489321	0.311989
F	-6.058239	-2.451048	0.696773
F	-6.071018	2.422549	0.684270
F	-4.202506	3.468778	0.293517
N	-2.255607	-0.006571	-0.246532

I	0.002421	-0.000480	-0.256217
N	2.260336	0.005538	-0.243223
F	4.206460	-3.470026	0.298851
F	6.074993	-2.424119	0.690376
F	6.062812	2.449117	0.704252
F	4.188679	3.487612	0.319809
C	5.018655	2.533966	-0.130646
C	2.922156	-1.150769	-0.223783
C	4.301706	-1.184796	-0.170690
C	4.998387	0.012205	-0.136804
C	4.295629	1.205801	-0.163595
C	2.916219	1.165048	-0.216850
C	5.031589	-2.509374	-0.145482
H	5.404860	-2.804848	-1.139502
H	5.389910	2.837400	-1.123025
H	2.327667	-2.060712	-0.233523
H	2.317087	2.071986	-0.221083
H	6.084187	0.014763	-0.061896

R = -Cl

H	-2.317902	1.464852	1.464388
H	-6.090306	-0.001035	-0.001416
H	-2.316708	-1.465605	-1.465461
C	-2.908137	-0.821453	-0.821473
C	-4.293505	-0.841862	-0.841952
C	-2.908791	0.820489	0.820120
C	-4.294150	0.840361	0.839873
C	-5.004202	-0.000853	-0.001181
Cl	-5.100453	1.902703	1.901925
Cl	-5.098727	-1.904283	-1.904109
N	-2.259505	-0.000383	-0.000522
I	-0.000001	-0.000041	0.000074
N	2.259506	0.000335	0.000574
Cl	5.099504	-1.901831	1.904816
Cl	5.099669	1.903683	-1.902675
C	2.908460	-0.820202	0.821809
C	4.293828	-0.840087	0.842239
C	5.004202	0.000857	0.001131
C	4.293830	0.841489	-0.840237
C	2.908470	0.821104	-0.820417
H	2.317290	-1.464320	1.466067
H	2.317323	1.465000	-1.464918
H	6.090307	0.001073	0.001346

R = -Br

H	-2.314451	1.761778	1.085847
H	-6.090332	-0.000452	-0.000281
H	-2.314022	-1.762191	-1.085780

C	-2.909251	-0.989202	-0.608683
C	-4.293741	-1.014117	-0.622981
C	-2.909483	0.988681	0.608681
C	-4.293958	1.013357	0.622710
C	-5.004188	-0.000410	-0.000186
Br	-5.172999	2.410964	1.480844
Br	-5.172321	-2.411598	-1.481040
N	-2.259565	-0.000196	0.000018
I	0.000038	0.000020	0.000114
N	2.259590	0.000234	0.000017
Br	5.172710	-2.410069	1.482207
Br	5.172488	2.410823	-1.481659
C	2.909423	-0.988423	0.609127
C	4.293911	-1.013165	0.623333
C	5.004172	0.000384	0.000075
C	4.293839	1.013810	-0.623307
C	2.909341	0.988921	-0.609124
H	2.314313	-1.761294	1.086564
H	2.314168	1.761697	-1.086638
H	6.090319	0.000437	0.000143

R = -I

H	-2.308414	2.065381	0.002697
H	-6.087125	-0.000004	-0.000139
H	-2.308408	-2.065378	-0.002776
C	-2.910411	-1.161219	-0.001587
C	-4.295328	-1.195342	-0.001617
C	-2.910415	1.161221	0.001478
C	-4.295331	1.195339	0.001436
C	-5.000663	-0.000002	-0.000109
I	-5.272264	3.015822	0.003624
I	-5.272256	-3.015816	-0.003860
N	-2.259121	0.000002	-0.000040
I	0.000001	0.000000	0.000006
N	2.259121	0.000000	0.000043
I	5.272258	-3.015819	0.001741
I	5.272264	3.015823	-0.001513
C	2.910411	-1.161222	0.000768
C	4.295328	-1.195345	0.000773
C	5.000662	-0.000003	0.000106
C	4.295330	1.195338	-0.000595
C	2.910414	1.161220	-0.000654
H	2.308409	-2.065381	0.001319
H	2.308413	2.065380	-0.001231
H	6.087125	-0.000005	0.000130

R = -CF_3

H	-2.333323	-1.437835	1.465090
H	-6.092001	0.068883	0.021213
H	-2.312769	1.487503	-1.457842
C	-5.053022	-1.708215	1.805312
C	-5.028180	1.819794	-1.774440
C	-2.912485	0.849979	-0.812984
C	-4.291315	0.888800	-0.830331
C	-2.923994	-0.786765	0.825477
C	-4.303062	-0.793537	0.855596
C	-5.002845	0.055883	0.015924
F	-5.672812	1.124978	-2.698045
F	-4.188621	2.642320	-2.382701
F	-5.917251	2.540657	-1.112029
F	-4.225078	-2.537775	2.419893
F	-5.950924	-2.421796	1.146851
F	-5.689412	-0.998670	2.723365
N	-2.258556	0.023997	0.003231
I	0.001732	-0.001796	-0.005008
N	2.262064	-0.026916	-0.009907
F	5.915946	-1.195370	-2.501661
F	4.179977	-2.456337	-2.596450
F	5.660911	-2.770170	-1.075426
F	4.240837	2.344923	2.584632
F	5.708660	2.650450	1.049448
F	5.959056	1.062091	2.461448
C	5.066011	1.731115	1.751712
C	2.911625	-0.853431	-0.829133
C	4.290287	-0.892856	-0.853196
C	5.006384	-0.058196	-0.012432
C	4.311153	0.792607	0.829518
C	2.931879	0.784591	0.807966
C	5.022286	-1.847628	-1.777115
H	2.308490	-1.488894	-1.472819
H	2.344608	1.433255	1.453150
H	6.095555	-0.071012	-0.013091

R = -SO_3

H	-2.344975	1.557681	-1.346428
H	-6.106016	-0.037387	0.006310
H	-2.327023	-1.592555	1.355487
H	-6.186747	-1.078201	3.300203
H	-6.200805	1.003262	-3.287640
C	-2.916386	-0.892580	0.766113
C	-4.297636	-0.912838	0.795307
C	-2.926364	0.851564	-0.756463
C	-4.307766	0.857223	-0.784320
C	-5.016703	-0.031606	0.005812
O	-4.184030	-3.151882	2.013563

O	-6.405050	-2.320002	1.253804
O	-5.270400	-1.355886	3.157219
O	-4.218989	3.097594	-2.002245
O	-6.430364	2.242021	-1.240769
O	-5.287249	1.290278	-3.145246
S	-5.142571	2.055786	-1.792816
S	-5.118785	-2.120011	1.804748
N	-2.260380	-0.017024	0.004491
I	0.002090	-0.005140	0.002755
N	2.264428	0.006863	0.000044
S	5.128590	1.822805	2.081605
S	5.140852	-1.777859	-2.091569
O	6.418728	1.278039	2.273487
O	4.199870	2.028313	3.119569
O	5.268822	3.175178	1.315143
O	5.297153	-3.129085	-1.326193
O	6.424755	-1.219807	-2.286960
O	4.211155	-1.992357	-3.126893
C	2.928049	-0.751500	-0.872614
C	4.309501	-0.772496	-0.887984
C	5.020734	0.021892	-0.004813
C	4.303980	0.808463	0.880906
C	2.922795	0.772406	0.870406
H	2.344652	-1.344906	-1.574178
H	2.335441	1.359419	1.574044
H	6.110046	0.027863	-0.006783
H	6.182753	3.322444	1.031772
H	6.213265	-3.266855	-1.045115

R = -CN

H	-2.325382	1.720852	1.141818
H	-6.101858	-0.004152	-0.006008
H	-2.321700	-1.725952	-1.146484
C	-4.969827	-2.051115	-1.361557
C	-2.916004	-0.967439	-0.642798
C	-4.305282	-1.000743	-0.665131
C	-2.918063	0.961838	0.636973
C	-4.307411	0.993968	0.656599
C	-5.014880	-0.003689	-0.004954
C	-4.974197	2.043767	1.351747
N	-5.501262	2.893077	1.914059
N	-5.495079	-2.900889	-1.924862
N	-2.261988	-0.002526	-0.002273
I	0.002312	-0.001663	0.000020
N	2.266626	-0.000875	0.002291
N	5.501885	-2.895465	1.926926
N	5.503755	2.895732	-1.916156
C	4.975996	-2.046490	1.363009
C	2.921360	-0.964849	0.643495

C	4.310664	-0.997112	0.665834
C	5.019516	-0.000006	0.004935
C	4.311303	0.996653	-0.657325
C	2.921980	0.963514	-0.637655
C	4.977306	2.046441	-1.353238
H	2.327623	-1.723442	1.147730
H	6.106494	0.000336	0.005976
H	2.328732	1.721724	-1.143040

R = -NO_2

H	-2.331905	1.800042	-1.089542
H	-6.124544	0.071883	-0.002338
H	-2.378015	-1.750550	1.090800
C	-2.939696	-0.955529	0.606173
C	-4.318058	-0.947666	0.610240
C	-2.914070	1.019362	-0.605774
C	-4.292180	1.045969	-0.611995
C	-5.036363	0.058278	-0.001473
O	-6.174881	2.144450	-1.274422
O	-4.254608	2.990675	-1.801895
O	-4.330918	-2.892166	1.800962
O	-6.228661	-1.999484	1.268527
N	-4.969368	2.159648	-1.289904
N	-5.023923	-2.044143	1.286937
N	-2.268019	0.023656	0.000676
I	-0.002465	-0.005240	0.000998
N	2.263083	-0.034147	0.000231
N	5.019173	2.030691	1.290830
N	4.964239	-2.167016	-1.295906
O	6.223905	1.985898	1.272424
O	4.326250	2.877711	1.806620
O	4.249401	-2.996749	-1.809885
O	6.169753	-2.151926	-1.280455
C	2.909041	-1.028413	-0.608669
C	4.287153	-1.054962	-0.615232
C	5.031425	-0.068696	-0.002525
C	4.313209	0.935800	0.611670
C	2.934848	0.943648	0.607863
H	2.326800	-1.807979	-1.094139
H	2.373233	1.737522	1.094446
H	6.119607	-0.082288	-0.003602

Table S7. $[(4\text{-R-py})_2I]^+$ adducts.

R = -NH_2

H	-2.335509	-1.463291	-1.451449
H	-4.797146	1.533400	1.522635
H	-4.799311	-1.531114	-1.518947

H	-2.333424	1.463626	1.453605
H	-6.891279	0.612826	0.608652
H	-6.892182	-0.608537	-0.603942
C	-2.927987	0.817635	0.812393
C	-4.293823	0.851310	0.845698
C	-2.929147	-0.816798	-0.809887
C	-4.295036	-0.849398	-0.842362
C	-5.030034	0.001271	0.001862
N	-6.369867	0.001858	0.002254
N	-2.246341	0.000146	0.001022
I	-0.000662	-0.001102	0.000262
N	2.244998	-0.002371	-0.000830
N	6.368525	-0.004808	-0.002918
C	2.927161	-0.814536	0.815396
C	4.293026	-0.848283	0.847830
C	5.028726	-0.004001	-0.002198
C	4.293169	0.841070	-0.851587
C	2.927315	0.808957	-0.817815
H	4.796741	-1.525921	1.528926
H	2.333001	-1.456094	1.461420
H	2.333260	1.451113	-1.463336
H	4.797027	1.518008	-1.533291
H	6.890428	0.601906	-0.613150
H	6.890356	-0.611290	0.607609

R = -NMe$_2$

H	-2.337720	2.060843	0.002880
H	-4.776586	-2.167498	-0.001868
H	-4.776631	2.167432	0.002678
H	-6.883708	1.849791	-0.887473
H	-6.884475	1.848066	0.891091
H	-8.181920	1.042862	0.000450
H	-2.337678	-2.060861	-0.001452
H	-6.883883	-1.849934	0.887660
H	-6.884251	-1.848123	-0.890904
H	-8.181906	-1.042979	-0.000511
C	-2.929603	-1.148919	-0.000564
C	-4.295136	-1.198606	-0.000779
C	-2.929627	1.148891	0.001834
C	-4.295160	1.198550	0.001705
C	-5.043925	-0.000036	0.000368
C	-7.113586	1.250342	0.001126
C	-7.113568	-1.250445	-0.000961
N	-6.386083	-0.000046	0.000197
N	-2.243845	-0.000008	0.000722
I	0.000026	0.000051	0.000820
N	2.243923	0.000127	0.000820
N	6.386162	0.000090	0.000181
C	7.113648	-1.250306	0.001811

C	7.113668	1.250474	-0.001739
C	2.929682	-1.148784	0.002626
C	4.295215	-1.198470	0.002493
C	5.044000	0.000101	0.000391
C	4.295235	1.198685	-0.001470
C	2.929700	1.149023	-0.001191
H	2.337758	-2.060725	0.004257
H	2.337792	2.060974	-0.002624
H	4.776671	-2.167358	0.004066
H	4.776706	2.167565	-0.003168
H	6.884630	-1.847478	0.892171
H	8.181985	-1.042840	0.000885
H	6.883668	-1.850303	-0.886392
H	6.884382	1.847610	-0.892055
H	8.182002	1.042991	-0.001134
H	6.883975	1.850516	0.886507

R = -OMe

H	-2.367567	1.401038	-1.209841
H	-2.286757	-1.544335	1.674187
H	-4.830673	1.404597	-1.290618
H	-4.777453	-1.676680	1.725055
H	-8.156843	0.298089	-0.312915
H	-6.921438	0.368412	-1.597800
H	-6.961571	1.622253	-0.315431
C	-2.902118	-0.913801	1.037739
C	-4.266966	-0.985051	1.064436
C	-2.945536	0.733543	-0.575217
C	-4.320138	0.730140	-0.615113
C	-5.009935	-0.148968	0.223016
C	-7.124695	0.562233	-0.538874
O	-6.322195	-0.258464	0.288688
N	-2.248672	-0.067141	0.229804
I	-0.000160	-0.001536	0.233217
N	2.248366	0.063992	0.234154
O	6.321792	0.254670	0.300956
C	7.125432	-0.555133	-0.536172
C	2.946347	-0.726187	-0.580215
C	4.321001	-0.722265	-0.618192
C	5.009630	0.145932	0.232169
C	4.265498	0.970964	1.083422
C	2.900687	0.900067	1.053942
H	2.369255	-1.385358	-1.224269
H	2.284450	1.522174	1.697786
H	4.832471	-1.387868	-1.301722
H	4.775082	1.653849	1.753767
H	8.157263	-0.293830	-0.305503
H	6.923484	-0.347610	-1.592750
H	6.962155	-1.617978	-0.326699

R = -OH

H	-2.332346	-0.015373	-2.058843
H	-2.323844	0.015515	2.065434
H	-4.807999	-0.016209	-2.148901
H	-4.816848	0.015869	2.156881
H	-6.742745	-0.006419	-0.784889
C	-2.923422	0.008652	1.158799
C	-4.291980	0.008860	1.208361
C	-2.925889	-0.008617	-1.148042
C	-4.298080	-0.009038	-1.190656
C	-5.007666	-0.000169	0.010199
O	-6.328452	0.000278	0.084969
N	-2.250194	0.000049	0.002244
I	0.000001	0.000090	0.000068
N	2.250194	0.000074	-0.002157
O	6.328444	0.000251	-0.085232
C	2.925987	-0.008597	1.148070
C	4.298180	-0.009030	1.190563
C	5.007665	-0.000166	-0.010353
C	4.291876	0.008866	-1.208454
C	2.923320	0.008668	-1.158770
H	2.332520	-0.015351	2.058921
H	2.323667	0.015542	-2.065355
H	4.808185	-0.016217	2.148762
H	4.816660	0.015874	-2.157020
H	6.742808	-0.006446	0.784591

R = -CH_3

H	-2.323969	2.061194	-0.007704
H	-4.803237	-2.148760	0.026666
H	-4.804487	2.148281	-0.000371
H	-2.323055	-2.060468	0.018148
H	-6.922175	-0.864928	0.513507
H	-6.869798	-0.060432	-1.052959
H	-6.925141	0.910782	0.417845
C	-2.921911	-1.153309	0.010686
C	-4.297362	-1.188161	0.014361
C	-2.922254	1.153610	-0.003799
C	-4.298156	1.187908	-0.000602
C	-5.025005	-0.000098	0.006352
C	-6.516280	-0.002197	-0.017662
N	-2.251830	0.000469	0.001130
I	0.000001	0.000517	0.001484
N	2.251830	0.000372	0.001187
C	6.516278	-0.001868	-0.017756
C	2.921929	-1.153414	-0.003852
C	4.297394	-1.188285	-0.000586

C	5.025006	-0.000209	0.006393
C	4.298124	1.187786	0.014468
C	2.922237	1.153505	0.010868
H	6.869678	-0.044726	-1.053813
H	6.925075	0.904653	0.431058
H	6.922338	-0.872237	0.500598
H	4.803294	-2.148947	-0.000402
H	2.323095	-2.060612	-0.007909
H	2.323929	2.061050	0.018477
H	4.804429	2.148097	0.026819

R = -CH_2F

H	-2.328877	0.094940	-2.049897
H	-4.794363	0.086402	2.172094
H	-4.823145	0.095145	-2.127710
H	-2.313000	0.086359	2.072550
H	-6.878802	0.979616	0.564812
F	-7.002549	0.093295	-1.229876
C	-2.916484	0.088305	1.168680
C	-4.290747	0.088342	1.209700
C	-2.925362	0.093064	-1.141043
C	-4.303295	0.093191	-1.176161
C	-5.009124	0.090829	0.018627
C	-6.507628	0.090977	0.035247
H	-6.879051	-0.799425	0.561662
N	-2.253161	0.090660	0.009715
I	-0.000041	0.090881	0.000063
N	2.253087	0.091403	-0.009464
F	7.002378	0.117048	1.230139
C	6.507543	0.093861	-0.034805
C	2.916475	0.070703	-1.168214
C	4.290735	0.070850	-1.209165
C	5.009042	0.092955	-0.018251
C	4.303154	0.114108	1.176309
C	2.925218	0.112666	1.141135
H	6.879651	-0.804674	-0.546715
H	6.878090	0.974087	-0.578671
H	4.794408	0.053684	-2.171379
H	2.313030	0.053887	-2.071957
H	2.328680	0.128839	2.049814
H	4.822948	0.131613	2.127733

R = -F

H	-2.320882	-0.019408	2.062949
H	-4.828576	-0.020348	2.148848
H	-4.828575	0.020017	-2.148743
H	-2.320881	0.019581	-2.062840
C	-2.921079	0.010938	-1.156682

C	-4.295618	0.011220	-1.204694
C	-2.921079	-0.010891	1.156791
C	-4.295618	-0.011440	1.204799
C	-4.976863	-0.000168	0.000053
F	-6.284231	-0.000333	0.000052
N	-2.254590	0.000089	0.000058
I	-0.000010	0.000158	-0.000079
N	2.254600	0.000165	-0.000159
F	6.284244	-0.000371	0.000255
C	2.920973	0.010998	1.156649
C	4.295508	0.011225	1.204805
C	4.976872	-0.000229	0.000124
C	4.295749	-0.011436	-1.204691
C	2.921204	-0.010831	-1.156819
H	4.828371	0.020023	2.148907
H	2.320687	0.019677	2.062749
H	2.321098	-0.019310	-2.063040
H	4.828803	-0.020340	-2.148686

R = -Cl

H	-2.321707	1.472970	1.448926
H	-4.814891	1.536202	1.514382
H	-4.819238	-1.534612	-1.499927
H	-2.325890	-1.471405	-1.441636
C	-2.923189	-0.823697	-0.804910
C	-4.298491	-0.855521	-0.834171
C	-2.920845	0.825275	0.813909
C	-4.296068	0.857120	0.847113
C	-4.996804	0.000812	0.007467
Cl	-6.698209	0.000851	0.009856
N	-2.253995	0.000792	0.003538
I	0.000002	0.000611	0.000079
N	2.253994	0.000051	-0.003363
Cl	6.698218	-0.002450	-0.010262
C	2.922850	-0.809682	0.820144
C	4.298139	-0.841706	0.849823
C	4.996802	-0.001468	-0.007598
C	4.296418	0.839534	-0.862861
C	2.921184	0.809035	-0.828930
H	4.818607	-1.508525	1.528085
H	2.325286	-1.445075	1.468917
H	2.322313	1.445086	-1.475855
H	4.815515	1.505790	-1.542728

R = -CHF$_2$

H	-2.318555	0.122093	-2.059863
H	-4.811502	0.085847	2.153334
H	-4.814183	0.089274	-2.146252

H	-2.315935	0.118811	2.063847
H	-6.921874	1.123939	0.005686
C	-2.918208	0.119636	1.159105
C	-4.295998	0.106454	1.198734
C	-2.919654	0.121475	-1.154340
C	-4.297512	0.108359	-1.192250
C	-4.998343	0.098832	0.003663
C	-6.512017	0.100109	0.004605
F	-6.963739	-0.539846	1.092174
F	-6.965065	-0.538093	-1.083367
N	-2.255330	0.127660	0.001956
I	-0.000042	0.131030	0.000469
N	2.255252	0.127652	-0.001085
F	6.963515	-0.541349	-1.091072
F	6.965084	-0.536851	1.084467
C	6.511938	0.099999	-0.004279
C	2.917957	0.117669	-1.158312
C	4.295747	0.104400	-1.198126
C	4.998261	0.098772	-0.003151
C	4.297602	0.110319	1.192853
C	2.919745	0.123404	1.155127
H	6.921843	1.123806	-0.006693
H	4.811105	0.082197	-2.152768
H	2.315558	0.115326	-2.062968
H	2.318765	0.125561	2.060727
H	4.814419	0.092836	2.146807

R = -I

H	-2.324911	1.727597	1.126624
H	-4.807177	1.806495	1.174215
H	-4.805179	-1.806639	-1.178962
H	-2.322974	-1.727233	-1.128001
C	-2.921314	-0.967181	-0.631733
C	-4.297932	-1.003552	-0.655574
C	-2.922387	0.967426	0.629560
C	-4.299064	1.003505	0.651519
C	-5.004349	-0.000086	-0.002517
I	-7.065333	-0.000198	-0.004149
N	-2.253418	0.000220	-0.000641
I	-0.000075	0.000226	0.000501
N	2.253353	0.000007	0.001290
I	7.066221	-0.000213	0.002559
C	2.921559	-0.973584	0.622432
C	4.298216	-1.010126	0.645325
C	5.004105	-0.000106	0.002014
C	4.298650	1.010030	-0.641574
C	2.921974	0.973578	-0.619447
H	4.805778	-1.818379	1.160383
H	2.323488	-1.738646	1.111275

H	2.324218	1.738648	-1.108674
H	4.806552	1.818200	-1.156425

R = -Br

H	-2.323847	2.062962	-0.000903
H	-4.812753	2.153464	-0.000994
H	-4.812758	-2.153463	0.000839
H	-2.323851	-2.062966	0.000858
C	-2.921595	-1.155010	0.000460
C	-4.297541	-1.199449	0.000449
C	-2.921592	1.155008	-0.000529
C	-4.297539	1.199450	-0.000577
C	-4.997035	0.000001	-0.000082
Br	-6.854043	0.000003	-0.000117
N	-2.253477	-0.000002	-0.000020
I	0.000000	-0.000004	0.000026
N	2.253477	-0.000002	0.000046
Br	6.854043	0.000003	0.000115
C	2.921595	-1.155010	-0.000463
C	4.297541	-1.199449	-0.000470
C	4.997035	0.000001	0.000038
C	4.297539	1.199449	0.000594
C	2.921592	1.155007	0.000563
H	4.812758	-2.153463	-0.000904
H	2.323851	-2.062966	-0.000877
H	2.323847	2.062962	0.000951
H	4.812753	2.153464	0.000998

R = -CF$_3$

H	-2.316356	-0.040694	-2.062865
H	-4.814977	-0.008053	2.150572
H	-4.814791	-0.044070	-2.151269
H	-2.316564	-0.005854	2.062433
C	-2.918445	-0.010517	1.157438
C	-4.296850	-0.009050	1.196963
C	-2.918300	-0.030038	-1.157956
C	-4.296811	-0.029152	-1.197729
C	-4.991599	-0.018156	-0.000471
C	-6.510985	0.024500	0.000060
F	-6.929458	1.281894	0.001800
F	-7.000465	-0.573061	1.073270
F	-7.001789	-0.570803	-1.073686
N	-2.256677	-0.020103	-0.000275
I	0.000002	-0.021448	-0.000103
N	2.256678	-0.020106	0.000075
F	7.000724	-0.570070	-1.074591
F	7.001528	-0.573788	1.072364
F	6.929451	1.281897	0.002053

C	6.510983	0.024500	0.000145
C	2.918464	-0.010507	-1.157600
C	4.296893	-0.009042	-1.197111
C	4.991597	-0.018160	0.000320
C	4.296766	-0.029167	1.197582
C	2.918279	-0.030053	1.157794
H	4.815030	-0.008022	-2.150709
H	2.316614	-0.005834	-2.062617
H	2.316299	-0.040723	2.062678
H	4.814741	-0.044109	2.151129

R = -SO_3H

H	-2.322953	1.778925	-1.090095
H	-4.835799	1.871173	-1.089970
H	-2.308731	-1.670375	1.174186
H	-4.816553	-1.716467	1.280384
H	-7.453536	-0.598001	-1.726130
C	-2.916399	-0.915764	0.681627
C	-4.293861	-0.940536	0.729006
C	-2.923588	1.022431	-0.591494
C	-4.303915	1.064533	-0.594533
C	-4.986850	0.063799	0.072767
O	-7.192141	1.350423	-0.342592
O	-7.117585	-0.415599	1.410078
O	-7.132029	-1.000875	-0.907035
S	-6.765720	0.093784	0.146617
N	-2.260635	0.049079	0.032561
I	-0.003363	0.047858	0.016647
N	2.253880	0.054751	0.001163
S	6.758476	0.158421	-0.095108
O	7.176416	-0.579650	-1.226979
O	7.110489	1.500445	0.138717
O	7.136351	-0.645264	1.190806
C	2.912041	-0.755747	-0.827223
C	4.292054	-0.768144	-0.875226
C	4.980119	0.090434	-0.036973
C	4.292151	0.938169	0.815960
C	2.914573	0.886742	0.809649
H	4.820072	-1.418547	-1.565983
H	2.307784	-1.397864	-1.462979
H	2.310920	1.522784	1.451908
H	4.818640	1.636580	1.459614
H	7.459400	-1.528871	0.964025

R = -CN

H	-2.319097	2.063473	0.001651
H	-4.813368	-2.154163	-0.001754
H	-2.319060	-2.063434	-0.001718

H	-4.813410	2.154154	0.001745
C	-6.427535	-0.000015	0.000018
C	-2.919459	-1.157508	-0.000969
C	-4.295838	-1.200924	-0.000985
C	-2.919481	1.157537	0.000918
C	-4.295859	1.200926	0.000966
C	-5.001172	-0.000007	0.000000
N	-7.574755	-0.000018	0.000034
N	-2.256216	0.000020	-0.000032
I	-0.000001	0.000042	-0.000035
N	2.256127	0.000034	-0.000038
N	7.574664	-0.000089	0.000042
C	2.919361	-1.157506	0.000894
C	4.295736	-1.200928	0.000946
C	5.001081	-0.000014	0.000002
C	4.295779	1.200923	-0.000965
C	2.919400	1.157547	-0.000953
C	6.427443	-0.000042	0.000025
H	2.318948	-2.063422	0.001610
H	2.319020	2.063486	-0.001688
H	4.813334	2.154148	-0.001715
H	4.813262	-2.154170	0.001709

R = -NO_2

H	-2.320474	1.719701	-1.129077
H	-4.844169	1.787422	-1.171172
H	-2.317145	-1.738006	1.125632
H	-4.840702	-1.809720	1.169060
C	-2.921388	-0.981695	0.631682
C	-4.300448	-1.021275	0.656301
C	-2.923259	0.962434	-0.634811
C	-4.302395	0.999831	-0.658703
C	-4.970328	-0.011251	-0.001023
O	-6.986921	0.896274	-0.584997
O	-6.985164	-0.921947	0.584070
N	-6.445977	-0.012414	-0.000616
N	-2.263818	-0.009114	-0.001744
I	-0.005082	-0.007336	-0.002312
N	2.253653	-0.005576	-0.002841
N	6.435807	-0.002425	-0.003744
O	6.976423	-0.911574	-0.587709
O	6.975302	0.907537	0.579994
C	2.912755	-0.977630	-0.635482
C	4.291877	-1.015096	-0.660010
C	4.960161	-0.003535	-0.003429
C	4.290637	1.007018	0.653442
C	2.911563	0.967463	0.629526
H	4.833378	-1.803098	-1.172136
H	2.309708	-1.735260	-1.128869

H	2.307587	1.724170	1.123194
H	4.831166	1.795839	1.165332

Table S8. $[I^-]\cdots[I_2]$ adducts.

I	-0.000765	0.000000	-2.958926
I	0.001530	0.000000	-0.000001
I	-0.000765	0.000000	2.958927

Table S9. Key geometrical parameters and interaction energies for the bis(Acetonitrile)-iodonium cation (BUKNAX) model. Otherwise specified, all models were optimized at the corresponding functional and def2-TZVP basis set.

Method	Dispersion	$d_{N\text{-}I}$ (Å)	$\alpha_{N\text{-}I\text{-}N}$ (°)	ΔE_{INT} (kcal/mol)
Experimental		2.198	180	
CCSD[a]		2.198	180	-36.98
CCSD(T)[a]		2.198	180	-37.82
MP2		2.191	180	-41.30
B3LYP		2.218	180	-41.50
	D3	2.220	180	-42.55
	D3BJ	2.214	180	-43.60
CAM-B3LYP		2.213	180	-42.05
	D3	2.214	180	-42.82
	D3BJ	2.211	180	-43.23
Lc-wPBE		2.203	180	-41.61
	D3	2.204	180	-42.42
	D3BJ	2.201	180	-42.92
M06-HF		2.194	180	-40.94
	D3	2.194	180	-41.06
M06-L		2.209	180	-43.35
	D3	2.209	180	-43.44
M06-2X		2.213	180	-41.39
	D3	2.213	180	-41.48
MN12-SX[b]		2.190	180	-40.09
PBEPBE		2.200	180	-44.39
	D3	2.200	180	-44.95
	D3BJ	2.198	180	-45.51
TPSSh		2.197	180	-43.80
TPSSTPSS		2.200	180	-44.16
	D3	2.201	180	-44.94
	D3BJ	2.197	180	-45.69
wB97XD		2.215	180	-41.90

[a] The data was obtained from single point (SP) calculations using the crystal structure of BUKNAX. Optimization at those levels of theory implies a high computational cost. [b] Method selected for the study of the $[N\cdots X\cdots N]^+$ halonium bond (X= Cl, Br, and I).

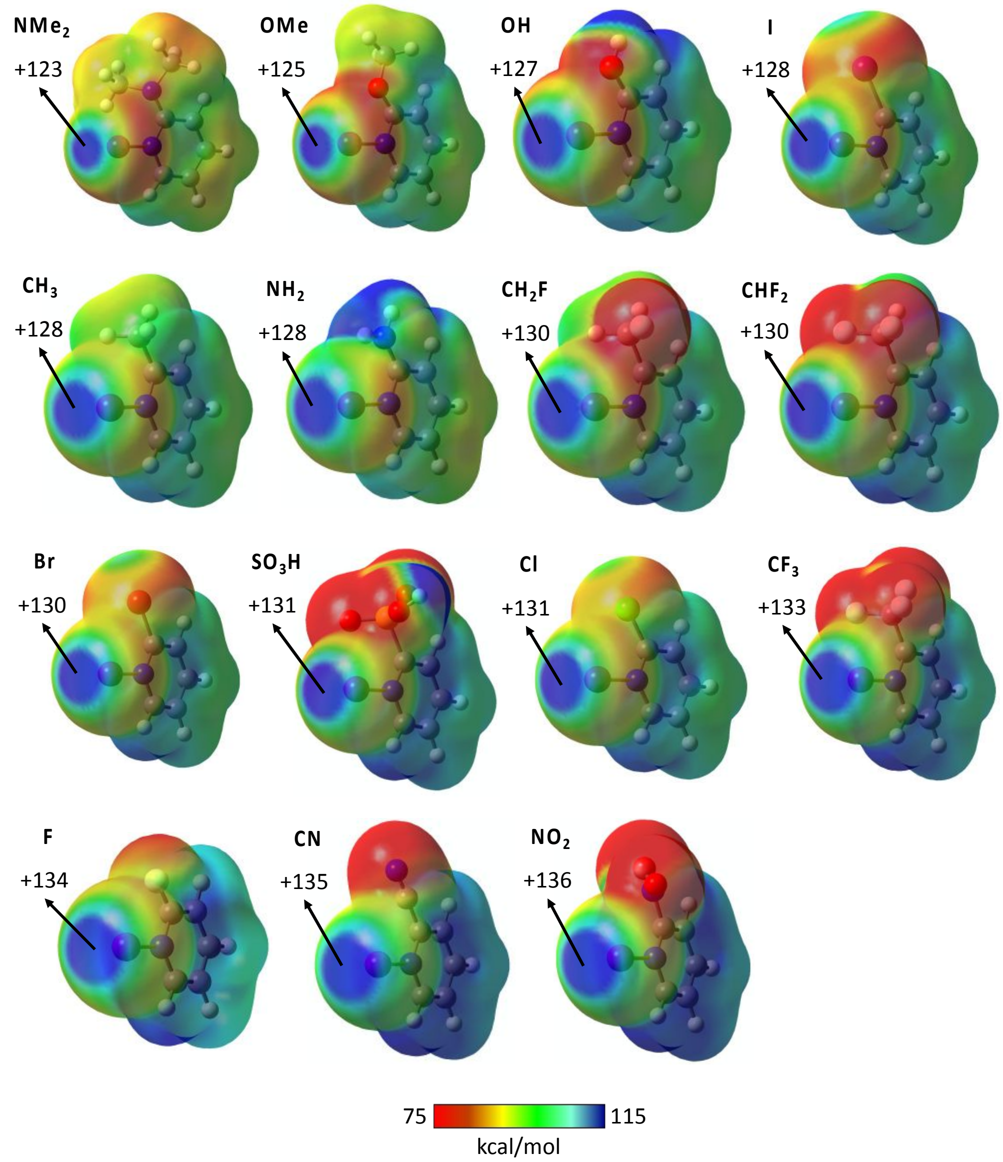

Figure S1. MEP maps for $[(2\text{-R-py})I]^+$ complexes (R = NMe_2, NH_2, OH, OMe, CH_3, CH_2F, CHF_2, CF_3, F, Cl, Br, I, CN, SO_3H, and NO_2) calculated at the MN12-SX/def2-TZVP level and plotted on the electron density isosurface (s = 0.001 Å). Energies are given in kcal/mol. Red and blue colours indicate less and more positive MEP values, respectively.

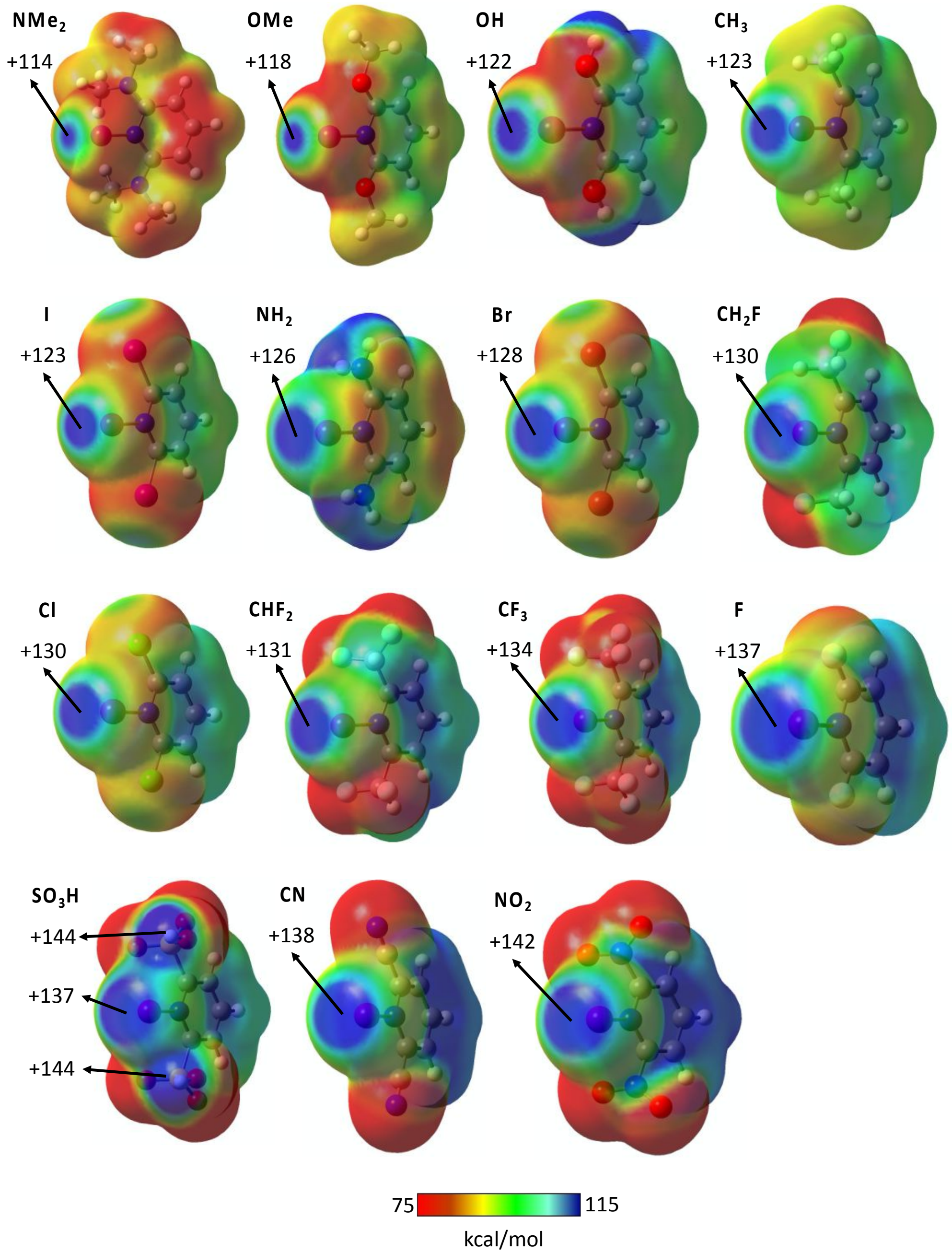

Figure S2. MEP maps for $[(2,6\text{-}R_2\text{-py})I]^+$ complexes (R = NMe_2, NH_2, OH, OMe, CH_3, CH_2F, CHF_2, CF_3, F, Cl, Br, I, CN, SO_3H, and NO_2) calculated at the MN12-SX/def2-TZVP level and plotted on the electron density isosurface (s = 0.001 Å). Energies are given in kcal/mol. Red and blue colours indicate less and more positive MEP values, respectively.

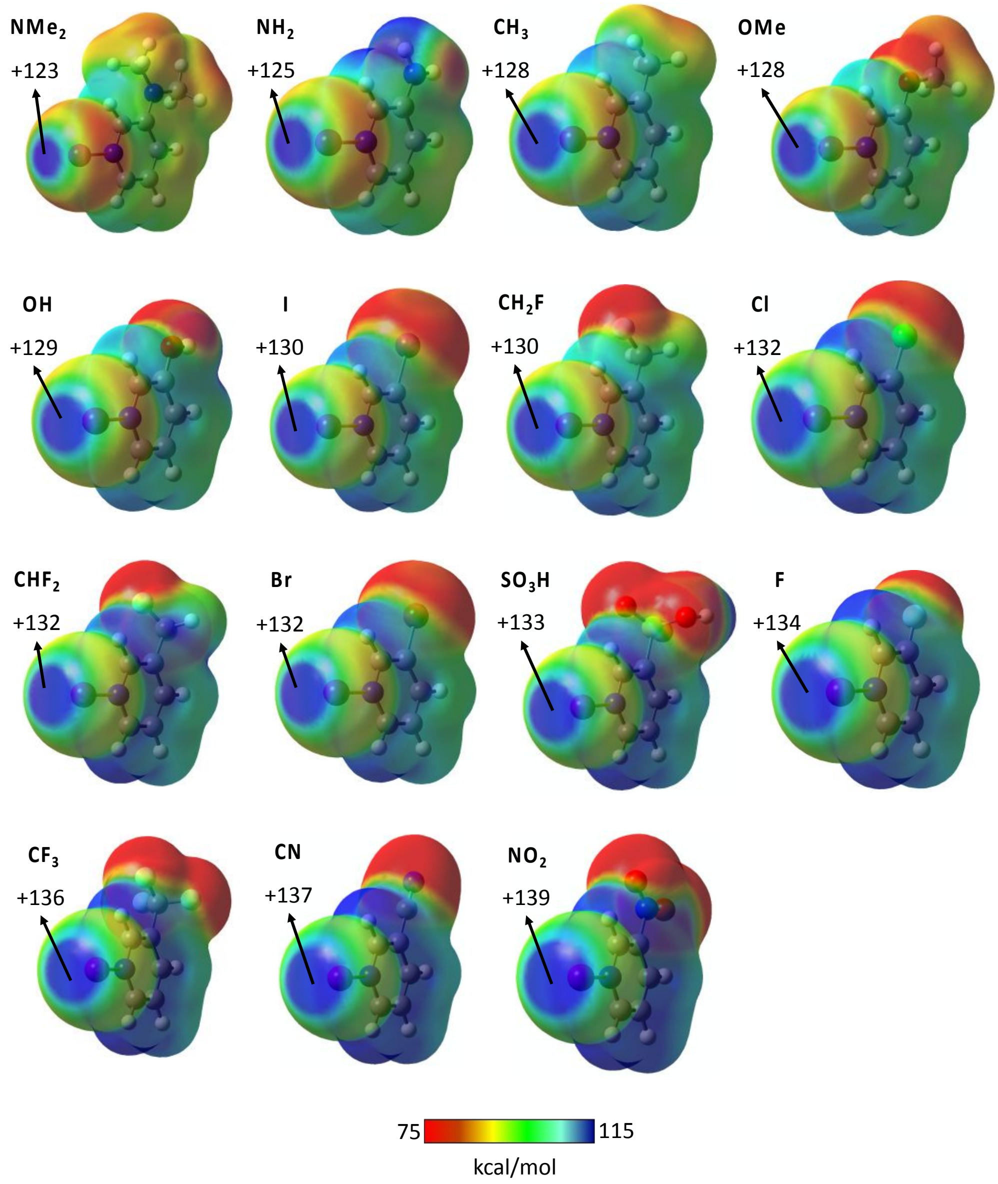

Figure S3. MEP maps for $[(3\text{-R-py})I]^+$ complexes (R = NMe_2, NH_2, OH, OMe, CH_3, CH_2F, CHF_2, CF_3, F, Cl, Br, I, CN, SO_3H, and NO_2) calculated at the MN12-SX/def2-TZVP level and plotted on the electron density isosurface (s = 0.001 Å). Energies are given in kcal/mol. Red and blue colours indicate less and more positive MEP values, respectively.

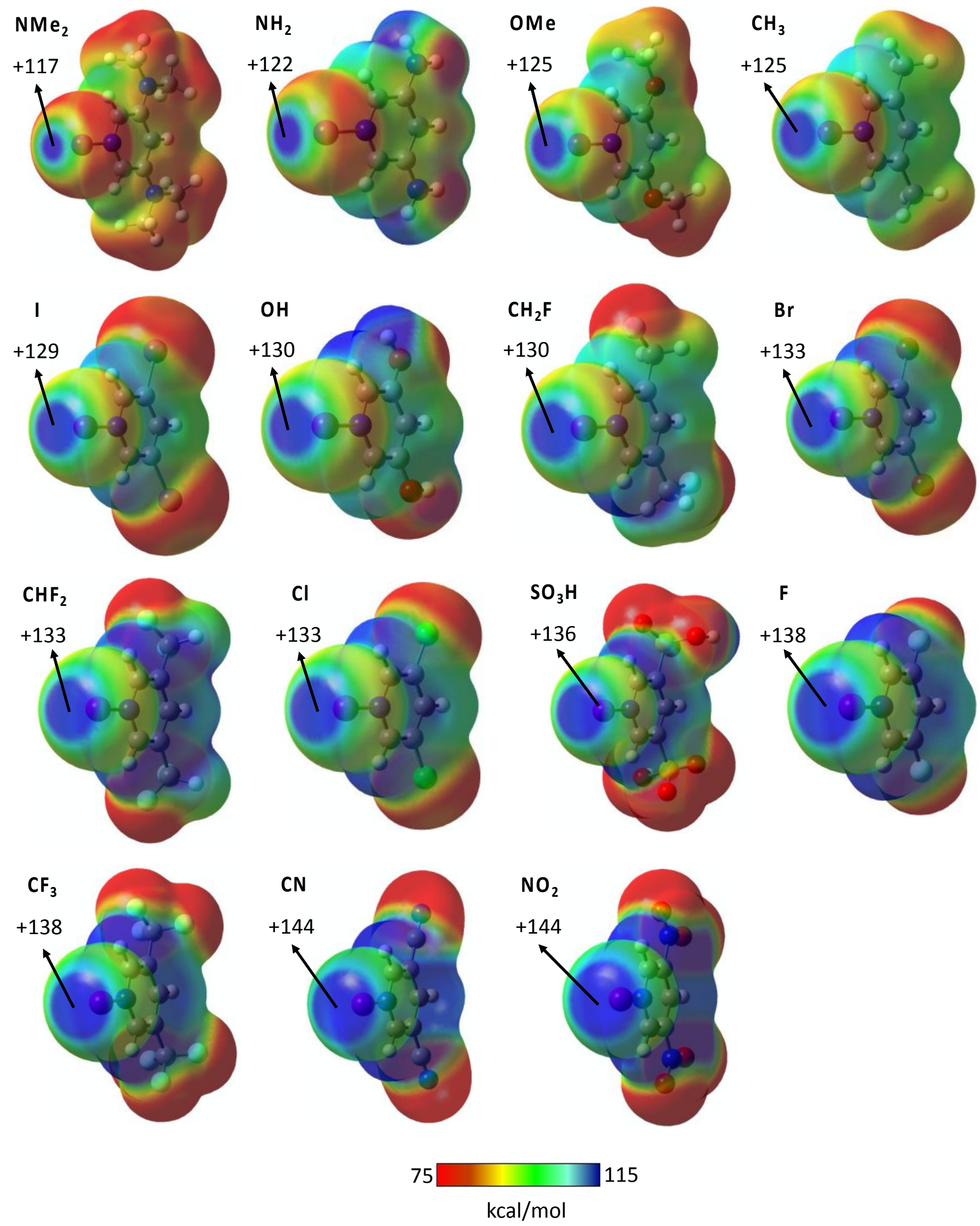

Figure S4. MEP maps for $[(3,5\text{-}R_2\text{-py})I]^+$ complexes (R = NMe_2, NH_2, OH, OMe, CH_3, CH_2F, CHF_2, CF_3, F, Cl, Br, I, CN, SO_3H, and NO_2) calculated at the MN12-SX/def2-TZVP level and plotted on the electron density isosurface (s = 0.001 Å). Energies are given in kcal/mol. Red and blue colours indicate less and more positive MEP values, respectively.

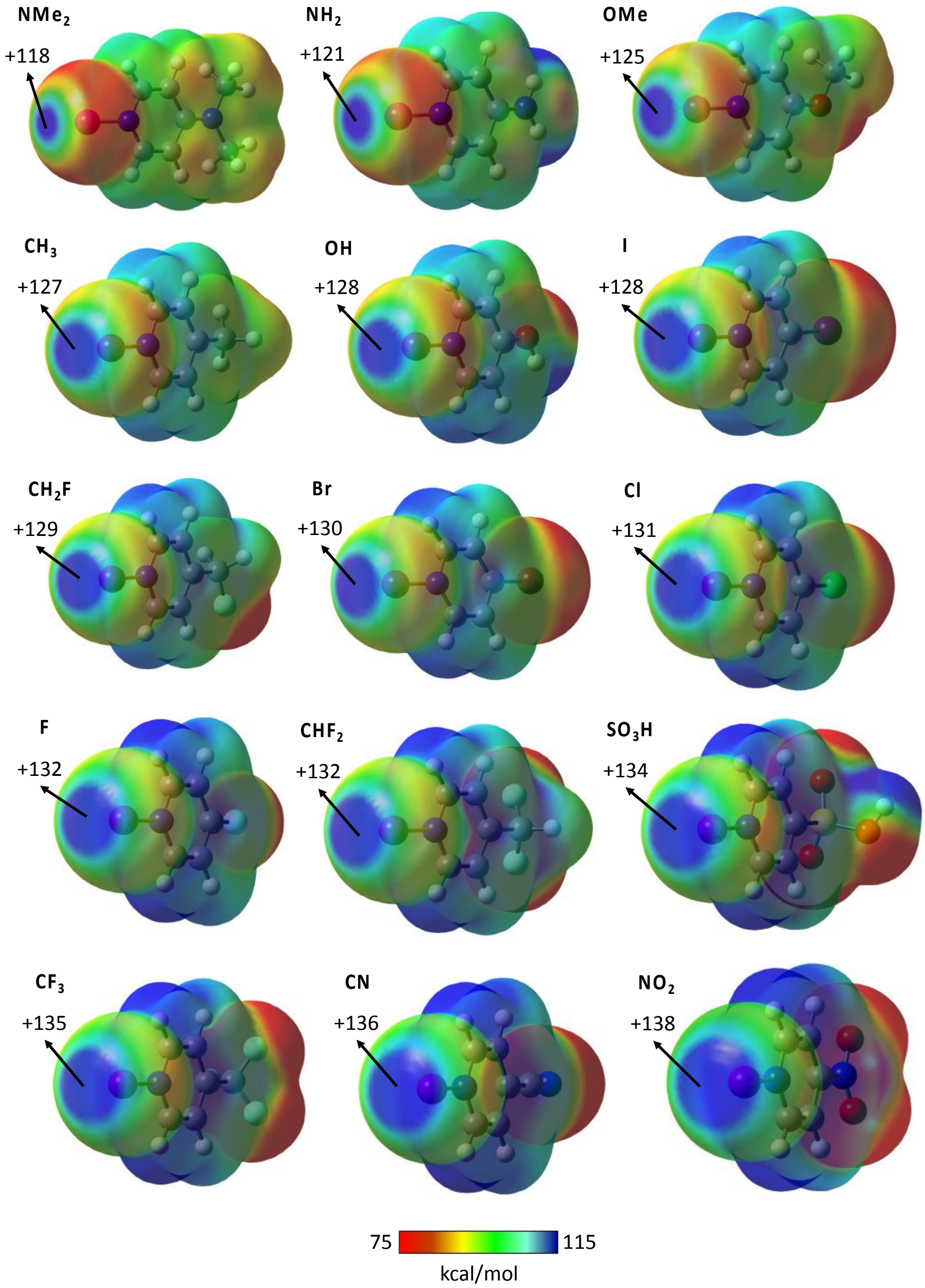

Figure S5. MEP maps for $[(4\text{-R-py})I]^+$ complexes (R = NMe_2, NH_2, OH, OMe, CH_3, CH_2F, CHF_2, CF_3, F, Cl, Br, I, CN, SO_3H, and NO_2) calculated at the MN12-SX/def2-TZVP level and plotted on the electron density isosurface (s = 0.001 Å). Energies are given in kcal/mol. Red and blue colours indicate less and more positive MEP values, respectively.

Table S10. Key geometrical parameters, $V_{s,max}$ of σ-holes, and interaction energies for the optimized $[(2\text{-R-py})_2I]^+$ complexes (2-R-py), calculated at the MN12-SX/def2-TZVP level.

**R**	**d_{N-I} (Å)**	**α_{N-I-N} (°)**	**$V_{s,max}$ (kcal/mol)**	**ΔE_{INT} (kcal/mol)**
OH	2.269	176.3	127	-42.10
OMe	2.270	175.7	125	-41.94
NH_2	2.261	176.9	128	-41.33
H	2.255	180.0	131	-41.30
CH_3	2.271	176.0	128	-40.91
NMe_2	2.279	180.0	123	-40.49
CH_2F	2.278	176.6	130	-40.45
CHF_2	2.287	180.0	130	-40.17
F	2.269	179.4	134	-39.44
Cl	2.274	176.5	131	-38.86
SO_3H	2.290	180.0	131	-38.73
CN	2.266	180.0	135	-38.50
CF_3	2.289	180.0	133	-38.36
Br	2.276	176.0	130	-38.28
I	2.277	176.0	128	-37.70
NO_2	2.306	177.8	136	-37.30

Table S11. Key geometrical parameters, $V_{s,max}$ of σ-holes, and interaction energies for the optimized $[(2,6\text{-}R_2\text{-}py)_2I]^+$ complexes (2,6-R_2-py), calculated at the MN12-SX/def2-TZVP level.

R	$d_{N\text{-}I}$ (Å)	$\alpha_{N\text{-}I\text{-}N}$ (°)	$V_{s,max}$ (kcal/mol)	ΔE_{INT} (kcal/mol)
SO_3H	2.311	180.0	137	-60.67
OH	2.293	180.0	122	-41.97
OMe	2.296	179.9	118	-41.49
H	2.255	180.0	131	-41.30
NH_2	2.280	180.0	126	-39.81
CH_3	2.292	180.0	123	-39.31
NMe_2	2.305	174.4	114	-38.20
CH_2F	2.296	180.0	130	-37.30
F	2.287	180.0	137	-37.21
Cl	2.302	180.0	130	-35.72
CHF_2	2.306	180.0	131	-35.32
Br	2.304	180.0	128	-34.63
I	2.315	180.0	123	-34.31
CN	2.283	180.0	138	-33.98
CF_3	2.337	179.7	134	-33.16
NO_2	2.358	180.0	142	-30.56

Table S12. Key geometrical parameters, $V_{s,max}$ of σ-holes, and interaction energies for the optimized $[(3\text{-}R\text{-}py)_2I]^+$ complexes (3-R-py), calculated at the MN12-SX/def2-TZVP level.

R	$d_{N\text{-}I}$ (Å)	$\alpha_{N\text{-}I\text{-}N}$ (°)	$V_{s,max}$ (kcal/mol)	ΔE_{INT} (kcal/mol)
NH_2	2.253	179.8	125	-43.10
NMe_2	2.252	180.0	123	-43.08
OMe	2.255	179.9	128	-42.22
OH	2.255	180.0	129	-42.08
CH_3	2.255	179.9	128	-41.77
CH_2F	2.256	180.0	130	-41.31
H	2.255	180.0	131	-41.30
F	2.257	179.8	134	-39.76
CHF_2	2.257	179.9	132	-39.64
Cl	2.257	179.9	132	-39.06
Br	2.257	179.9	132	-38.75
I	2.257	180.0	130	-38.50
CF_3	2.258	179.7	136	-38.41
SO_3H	2.259	179.6	133	-38.02
CN	2.260	180.0	137	-36.24
NO_2	2.260	180.0	139	-36.15

Table S13. Key geometrical parameters, $V_{s,max}$ of σ-holes, and interaction energies for the optimized $[(3,5\text{-}R_2\text{-}py)_2I]^+$ complexes (3,5-R_2-py), calculated at the MN12-SX/def2-TZVP level.

R	$d_{N\text{-}I}$ (Å)	$\alpha_{N\text{-}I\text{-}N}$ (°)	$V_{s,max}$ (kcal/mol)	ΔE_{INT} (kcal/mol)
NMe_2	2.251	179.8	117	-44.46
NH_2	2.252	179.9	122	-44.35
OMe	2.252	179.8	125	-42.31
CH_3	2.254	180.0	125	-41.99
OH	2.255	179.8	130	-41.47
H	2.255	180.0	131	-41.30
CH_2F	2.256	179.9	130	-40.18
F	2.259	180.0	138	-38.06
CHF_2	2.258	179.4	133	-37.76
Cl	2.260	180.0	133	-36.90
Br	2.260	180.0	133	-36.36
I	2.259	180.0	129	-36.12
CF_3	2.260	179.9	138	-35.48
SO_3H	2.263	180.0	136	-35.02
CN	2.264	180.0	144	-31.30
NO_2	2.266	180.0	144	-30.96

Table S14. Key geometrical parameters, $V_{s,max}$ of σ-holes, and interaction energies for the optimized $[(4\text{-}R\text{-}py)_2I]^+$ complexes (4-R-py), calculated at the MN12-SX/def2-TZVP level.

R	$d_{N\text{-}I}$ (Å)	$\alpha_{N\text{-}I\text{-}N}$ (°)	$V_{s,max}$ (kcal/mol)	ΔE_{INT} (kcal/mol)
NH_2	2.246	180.0	121	-44.65
NMe_2	2.244	180.0	118	-44.32
OMe	2.249	179.9	125	-42.91
OH	2.250	180.0	128	-42.39
CH_3	2.252	180.0	127	-41.96
H	2.255	180.0	131	-41.30
CH_2F	2.253	180.0	129	-40.83
F	2.255	180.0	132	-40.16
Cl	2.254	180.0	131	-39.62
CHF_2	2.255	179.8	132	-39.30
I	2.253	180.0	128	-39.26
Br	2.253	180.0	130	-39.15
CF_3	2.257	179.9	135	-38.04
SO_3H	2.257	179.8	134	-37.41
CN	2.256	180.0	136	-36.00
NO_2	2.259	180.0	138	-35.79

a **b**

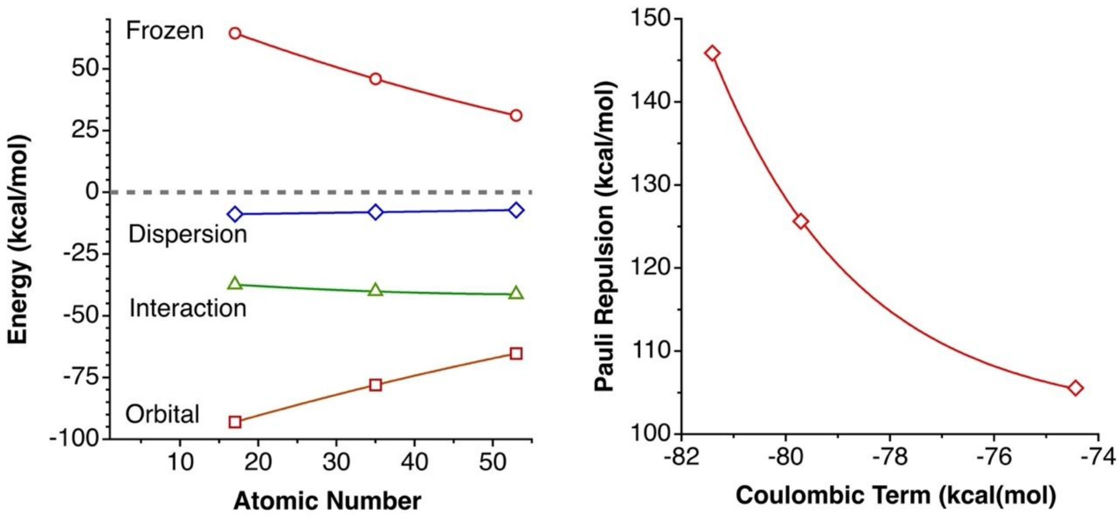

Figure S6. a) Dependence on the atomic number of the central halogen atom (X = Cl, Br, and I) of the frozen ($R^2 = 0.996$), dispersion ($R^2 = 0.999$) and orbital contributions ($R^2 = 0.998$) to the interaction energy and the net interaction energy ($R^2 = 0.949$) between the py donors and the $[X\text{-py}]^+$ cations. **b**) Correlation between the Coulombic (ΔE_{ELEC}) and Pauli exchange-repulsion (ΔE_{PAULI}) term ($R^2 = 0.999$).

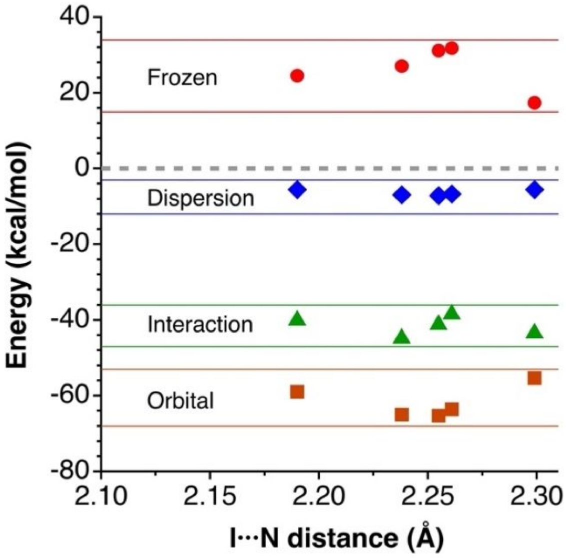

Figure S7. a) Dependence on the I···N distance of the EDA contribution to the interaction energy and the net interaction energy between the N-donors (D) and the $[I\text{-D}]^+$ cations.

Table S15. EDA for the optimized $[(2\text{-R-py})_2I]^+$ complexes, calculated at the MN12-SX/def2-TZVP level. The percentage represents its contribution to the total attractive interaction energy. Energies are given in kcal/mol.

R	ΔE_{PAULI}	ΔE_{ELEC}	ΔE_{DISP}	ΔE_{POL}	ΔE_{CT}	ΔE_{INT}
OH	100.68	-72.47 (50.8 %)	-7.84 (5.5 %)	-26.13 (18.3 %)	-36.34 (25.4 %)	-42.10
OMe	101.78	-72.78 (50.6 %)	-7.96 (5.5 %)	-26.56 (18.5 %)	-36.42 (25.3 %)	-41.94
NH_2	114.64	-78.92 (50.6 %)	-8.52 (5.5 %)	-28.61 (18.3 %)	-39.91 (25.6 %)	-41.33
H	105.56	-74.44 (50.7 %)	-7.16 (4.9 %)	-25.94 (17.7 %)	-39.32 (26.8 %)	-41.29
CH_3	108.05	-75.00 (50.3 %)	-8.57 (5.8 %)	-26.62 (17.9 %)	-38.78 (26.0 %)	-40.91
NMe_2	105.22	-71.61 (49.1 %)	-9.72 (6.7 %)	-27.54 (18.9 %)	-36.84 (25.3 %)	-40.50
CH_2F	102.53	-71.60 (50.1 %)	-8.61 (6.0 %)	-26.28 (18.4 %)	-36.49 (25.5 %)	-40.45
CHF_2	96.51	-68.11 (49.8 %)	-8.54 (6.3 %)	-25.41 (18.6 %)	-34.62 (25.3 %)	-40.17
F	96.82	-67.66 (49.7 %)	-7.32 (5.4 %)	-25.63 (18.8 %)	-35.65 (26.2 %)	-39.44
Cl	98.76	-67.59 (49.1 %)	-8.18 (5.9 %)	-26.20 (19.0 %)	-35.65 (25.9 %)	-38.86
SO_3H	94.81	-65.10 (48.8 %)	-9.08 (6.8 %)	-25.58 (19.2 %)	-33.77 (25.3 %)	-38.73
CN	99.52	-67.88 (49.2 %)	-7.89 (5.7 %)	-25.48 (18.5 %)	-36.76 (26.6 %)	-38.50
CF_3	95.44	-65.43 (48.9 %)	-8.48 (6.3 %)	-25.22 (18.9 %)	-34.66 (25.9 %)	-38.36
Br	99.04	-67.01 (48.8 %)	-8.31 (6.1 %)	-26.26 (19.1 %)	-35.73 (26.0 %)	-38.28
I	101.18	-67.56 (48.6 %)	-8.51 (6.1 %)	-26.53 (19.1 %)	-36.28 (26.1 %)	-37.69
NO_2	87.63	-59.60 (47.7 %)	-8.15 (6.5 %)	-24.85 (19.9 %)	-32.33 (25.9 %)	-37.30

Table S16. EDA for the optimized $[(2,6\text{-R}_2\text{-py})_2I]^+$ complexes, calculated at the MN12-SX/def2-TZVP level. The percentage represents the contribution to the total attractive interaction energy. Energies are given in kcal/mol.

R	ΔE_{PAULI}	ΔE_{ELEC}	ΔE_{DISP}	ΔE_{POL}	ΔE_{CT}	ΔE_{INT}
SO_3H	150.66	-102.65 (48.6 %)	-19.52 (9.2 %)	-43.23 (20.5 %)	-45.95 (21.7 %)	-60.67
OH	94.38	-69.09 (50.7 %)	-8.54 (6.3 %)	-25.26 (18.5 %)	-33.46 (24.5 %)	-41.97
OMe	96.52	-69.56 (50.4 %)	-8.78 (6.4 %)	-26.04 (18.9 %)	-33.63 (24.4 %)	-41.49
H	105.56	-74.44 (50.7 %)	-7.16 (4.9 %)	-25.94 (17.7 %)	-39.32 (26.8 %)	-41.29
NH_2	122.27	-81.88 (50.5 %)	-9.93 (6.1 %)	-29.76 (18.4 %)	-40.50 (25.0 %)	-39.81
CH_3	112.15	-75.47 (49.8 %)	-10.75 (7.1 %)	-26.48 (17.5 %)	-38.75 (25.6 %)	-39.30
NMe_2	103.88	-66.13 (46.5 %)	-13.32 (9.4 %)	-27.42 (19.3 %)	-35.21 (24.8 %)	-38.20
CH_2F	108.55	-71.21 (48.8 %)	-10.83 (7.4 %)	-26.67 (18.3 %)	-37.13 (25.5 %)	-37.30
F	88.25	-60.82 (48.5 %)	-7.49 (6.0 %)	-24.42 (19.5 %)	-32.73 (26.1 %)	-37.21
Cl	94.53	-62.29 (47.8 %)	-9.86 (7.6 %)	-24.50 (18.8 %)	-33.59 (25.8 %)	-35.72
CHF_2	103.45	-66.46 (47.9 %)	-10.84 (7.8 %)	-26.10 (18.8 %)	-35.38 (25.5 %)	-35.32
Br	97.51	-62.64 (47.4 %)	-10.88 (8.2 %)	-24.73 (18.7 %)	-33.90 (25.7 %)	-34.64
I	102.25	-64.11 (46.9 %)	-12.65 (9.3 %)	-24.40 (17.9 %)	-35.39 (25.9 %)	-34.30
CN	93.76	-59.61 (46.7 %)	-8.65 (6.8 %)	-24.48 (19.2 %)	-35.00 (27.4 %)	-33.99

CF_3	90.08	-58.27 (47.3 %)	-11.09 (9.0 %)	-23.23 (18.8 %)	-30.67 (24.9 %)	-33.19
NO_2	75.59	-45.93 (43.3 %)	-9.33 (8.8 %)	-22.45 (21.1 %)	-28.45 (26.8 %)	-30.56

Table S17. EDA for the optimized $[(3\text{-R-py})_2I]^+$ complexes, calculated at the MN12-SX/def2-TZVP level. The percentage represents the contribution to the total attractive interaction energy. Energies are given in kcal/mol.

R	ΔE_{PAULI}	ΔE_{ELEC}	ΔE_{DISP}	ΔE_{POL}	ΔE_{CT}	ΔE_{INT}
NH_2	107.51	-77.16 (51.2 %)	-7.35 (4.9 %)	-26.54 (17.6 %)	-39.56 (26.3 %)	-43.10
NMe_2	108.89	-77.79 (51.2%)	-7.44 (4.9 %)	-27.15 (17.9 %)	-39.60 (26.1 %)	-43.08
OMe	105.72	-75.37 (50.9 %)	-7.20 (4.9 %)	-26.26 (17.7 %)	-39.11 (26.4 %)	-42.22
OH	105.09	-74.91 (50.9 %)	-7.18 (4.9 %)	-26.06 (17.7 %)	-39.02 (26.5 %)	-42.08
CH_3	106.51	-75.40 (50.8 %)	-7.23 (4.9 %)	-26.57 (17.9 %)	-39.09 (26.4 %)	-41.77
CH_2F	105.10	-74.19 (50.7 %)	-7.15 (4.9 %)	-26.45 (18.1 %)	-38.62 (26.4 %)	-41.31
H	105.56	-74.44 (50.7 %)	-7.16 (4.9 %)	-25.94 (17.7 %)	-39.32 (26.8 %)	-41.29
F	102.81	-71.24 (50.0 %)	-7.03 (4.9 %)	-25.90 (18.2 %)	-38.40 (26.9 %)	-39.76
CHF_2	103.68	-71.59 (50.0 %)	-7.06 (4.9 %)	-26.77 (18.7 %)	-37.89 (26.4 %)	-39.64
Cl	102.87	-70.32 (49.5 %)	-7.08 (5.0 %)	-26.94 (19.0 %)	-37.60 (26.5 %)	-39.06
Br	102.92	-69.89 (49.3 %)	-7.10 (5.0 %)	-27.33 (19.3 %)	-37.36 (26.4 %)	-38.75
I	103.48	-69.77 (49.1 %)	-7.13 (5.0 %)	-27.49 (19.4 %)	-37.59 (26.5 %)	-38.49
CF_3	102.33	-69.50 (49.4 %)	-7.01 (5.0 %)	-26.50 (18.8 %)	-37.73 (26.8 %)	-38.41
SO_3H	101.48	-68.52 (49.1 %)	-6.94 (5.0 %)	-27.03 (19.4 %)	-37.02 (26.5 %)	-38.03
CN	100.41	-65.81 (48.2 %)	-6.93 (5.1 %)	-25.83 (18.9 %)	-38.08 (27.9 %)	-36.24
NO_2	99.83	-65.57 (48.2 %)	-6.83 (5.0 %)	-25.99 (19.1 %)	-37.59 (27.6 %)	-36.15

Table S18. EDA for the optimized $[(3,5\text{-R}_2\text{-py})_2I]^+$ complexes, calculated at the MN12-SX/def2-TZVP level. The percentage represents the contribution to the total attractive interaction energy. Energies are given in kcal/mol.

R	ΔE_{PAULI}	ΔE_{ELEC}	ΔE_{DISP}	ΔE_{POL}	ΔE_{CT}	ΔE_{INT}
NMe_2	111.24	-80.30 (51.6 %)	-7.69 (4.9 %)	-28.16 (18.1 %)	-39.54 (25.4 %)	-44.46
NH_2	108.81	-78.98 (51.6 %)	-7.51 (4.9 %)	-26.90 (17.6 %)	-39.76 (26.0 %)	-44.35
OMe	106.84	-75.82 (50.8 %)	-7.35 (4.9 %)	-26.80 (18.0 %)	-39.18 (26.3 %)	-42.31
CH_3	107.43	-76.02 (50.9 %)	-7.29 (4.9 %)	-26.71 (17.9 %)	-39.40 (26.4 %)	-41.99
OH	104.77	-74.06 (50.6 %)	-7.25 (5.0 %)	-26.00 (17.8 %)	-38.92 (26.6 %)	-41.47
H	105.56	-74.44 (50.7 %)	-7.16 (4.9 %)	-25.94 (17.7 %)	-39.32 (26.8 %)	-41.29
CH_2F	105.07	-72.82 (50.1 %)	-7.14 (4.9 %)	-26.89 (18.5 %)	-38.39 (26.4 %)	-40.18
F	100.18	-67.86 (49.1 %)	-6.94 (5.0 %)	-25.22 (18.2 %)	-38.23 (27.7 %)	-38.06
CHF_2	102.04	-68.56 (49.0 %)	-6.97 (5.0 %)	-26.93 (19.3 %)	-37.35 (26.7 %)	-37.76
Cl	100.46	-66.33 (48.3 %)	-7.02 (5.1 %)	-27.12 (19.7 %)	-36.88 (26.9 %)	-36.90

Br	100.58	-65.63 (47.9 %)	-7.05 (5.2 %)	-27.60 (20.2 %)	-36.65 (26.8 %)	-36.36
I	101.60	-65.75 (47.7 %)	-7.12 (5.2 %)	-27.78 (20.2 %)	-37.07 (26.9 %)	-36.12
CF_3	99.42	-64.58 (47.9 %)	-6.88 (5.1 %)	-26.44 (19.6 %)	-37.00 (27.4 %)	-35.48
SO_3H	97.91	-63.15 (47.5 %)	-6.77 (5.1 %)	-27.06 (20.4 %)	-35.95 (27.0 %)	-35.02
CN	95.88	-57.60 (45.3 %)	-6.74 (5.3 %)	-25.86 (20.3 %)	-36.98 (29.1 %)	-31.30
NO_2	94.65	-56.91 (45.3 %)	-6.53 (5.2 %)	-26.04 (20.7 %)	-36.13 (28.8 %)	-30.96

Table S19. EDA for the optimized $[(4\text{-R-py})_2I]^+$ complexes, calculated at the MN12-SX/def2-TZVP level. The percentage represents its contribution to the total attractive interaction energy. Energies are given in kcal/mol.

**R**	**ΔE_{PAULI}**	**ΔE_{ELEC}**	**ΔE_{DISP}**	**ΔE_{POL}**	**ΔE_{CT}**	**ΔE_{INT}**
NH_2	111.21	-81.10 (52.0 %)	-7.52 (4.8 %)	-27.37 (17.6 %)	-39.88 (25.6 %)	-44.65
NMe_2	112.53	-81.35 (51.9 %)	-7.55 (4.8 %)	-28.41 (18.1 %)	-39.53 (25.2 %)	-44.31
OMe	108.61	-77.81 (51.4 %)	-7.36 (4.9 %)	-27.22 (18.0 %)	-39.13 (25.8 %)	-42.91
OH	107.70	-76.75 (51.1 %)	-7.32 (4.9 %)	-26.82 (17.9 %)	-39.19 (26.1 %)	-42.38
CH_3	107.28	-75.99 (50.9 %)	-7.25 (4.9 %)	-26.66 (17.9 %)	-39.34 (26.4 %)	-41.95
H	105.56	-74.44 (50.7 %)	-7.16 (4.9 %)	-25.94 (17.7 %)	-39.32 (26.8 %)	-41.29
CH_2F	106.04	-74.09 (50.4 %)	-7.19 (4.9 %)	-26.60 (18.1 %)	-38.98 (26.5 %)	-40.83
F	104.53	-72.66 (50.2 %)	-7.16 (4.9 %)	-26.22 (18.1 %)	-38.64 (26.7 %)	-40.16
Cl	104.78	-72.10 (49.9 %)	-7.14 (4.9 %)	-26.94 (18.7 %)	-38.23 (26.5 %)	-39.63
CHF_2	104.21	-71.40 (49.8 %)	-7.10 (4.9 %)	-26.60 (18.5 %)	-38.42 (26.8 %)	-39.30
I	105.36	-71.89 (49.7 %)	-7.15 (4.9 %)	-27.48 (19.0 %)	-38.09 (26.3 %)	-39.26
Br	104.94	-71.58 (49.7 %)	-7.14 (5.0 %)	-27.66 (19.2 %)	-37.71 (26.2 %)	-39.15
CF_3	102.85	-69.25 (49.2 %)	-7.04 (5.0 %)	-26.56 (18.9 %)	-38.03 (27.0 %)	-38.03
SO_3H	102.53	-68.31 (48.8 %)	-7.02 (5.0 %)	-26.94 (19.3 %)	-37.66 (26.9 %)	-37.41
CN	102.10	-66.44 (48.1 %)	-6.98 (5.1 %)	-26.24 (19.0 %)	-38.44 (27.8 %)	-36.00
NO_2	100.91	-65.65 (48.0 %)	-6.94 (5.1 %)	-25.98 (19.0 %)	-38.13 (27.9 %)	-35.79

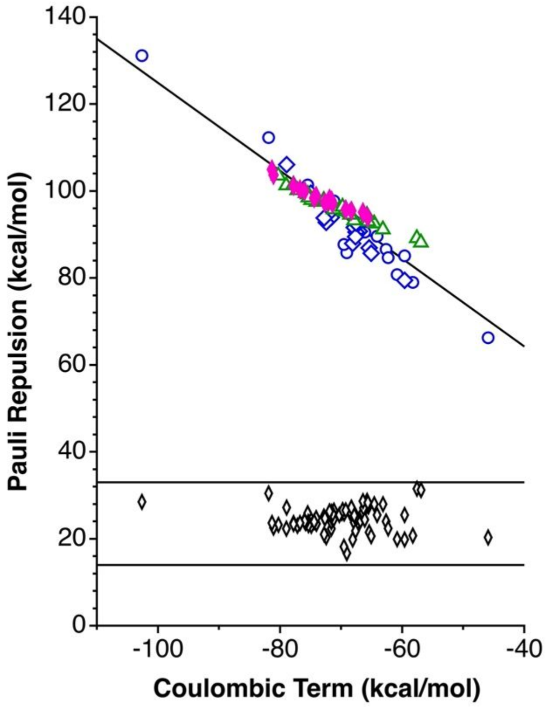

Figure S8. Dependence on the Coulombic (ΔE_{ELEC}) term to the Pauli exchange-repulsion (ΔE_{PAULI}) term for all the 75 substituted pyridines studied in this work ($R^2 = 0.854$). The net frozen component is repulsive.

Chart S1. Linear least-squares fitting equations for Figure 7.

a) $V_{s,max}$ (4-R-py) = 128.7 + 10.64·σ_p ($R^2 = 0.89$) [Eq. S1]

b) $V_{s,max}$ (3-R-py) = 127.7 + 15.07·σ_m ($R^2 = 0.86$) [Eq. S2]

$V_{s,max}$ (3,5-R_2-py) = 125.6 + 26.19·σ_m ($R^2 = 0.84$) [Eq. S3]

c) $V_{s,max}$ (2-R-py) = 129.4 + 6.16·σ_p ($R^2 = 0.67$) [Eq. S4]

Chart S2. 2nd order polynomial least-squares fitting equations for Figure 8.

a) E_{INT} (4-R-py) = 104.1 – 2.74·$V_{s,max}$ + 0.013·$(V_{s,max})^2$ ($R^2 = 0.89$) [Eq. S5]

b) E_{INT} (3-R-py) = 8.55 – 1.22·$V_{s,max}$ + 0.007·$(V_{s,max})^2$ ($R^2 = 0.82$) [Eq. S6]

E_{INT} (3,5-R_2-py) = -0.94 – 1.09·$V_{s,max}$ + 0.006·$(V_{s,max})^2$ ($R^2 = 0.84$) [Eq. S7]

Chart S3. Linear least-squares fitting equations for Figure 11.

a) $d_{I\text{-}N}$ (4-R-py) = 2.25 + 0.008·σ_p ($R^2 = 0.90$) [Eq. S8]

b) E_{INT} (4-R-py) = – 40.73 + 5.58·σ_p ($R^2 = 0.96$) [Eq. S9]

Chart S4. Linear least-squares fitting equations for Figure 12.

a) $d_{I\text{-}N}$ (3-R-py) = 2.25 + 0.008·σ_m (R^2 = 0.91) [Eq. S10]

$d_{I\text{-}N}$ (3,5-R_2-py) = 2.25 + 0.016·σ_m (R^2 = 0.89) [Eq. S11]

b) E_{INT} (3-R-py) = -41.89 + 8.26·σ_m (R^2 = 0.91) [Eq. S12]

E_{INT} (3,5-R_2-py) = -42.01 + 15.56·σ_m (R^2 = 0.94) [Eq. S13]

Table S20. Experimental I···N distance and N-I-N bond angle for compounds containing a [N···I···N] central framework found in the CSD, represented in Figure 3.

Refcode	Donor	$d_{N\text{-}I}$ (Å)	$\alpha_{N\text{-}I\text{-}N}$ (°)	Refcode	Donor	$d_{N\text{-}I}$ (Å)	$\alpha_{N\text{-}I\text{-}N}$ (°)
**PYRIDI**	Pyridine	2.164	180.0	**DOVYOE01**	Pyridine	2.257	180.0
**BUKNAX**	Acetonitrile	2.198	180.0	**HUMMAD**	Pyridine	2.258	177.7
**YOFKOW**	Pyridine	2.227	180.0	**NOMBOJ**	Pyridine	2.258	176.8
**MIYJIP**	Pyridine	2.236	179.4	**HUMMAD04**	Pyridine	2.258	180.0
**YOFKOW**	Pyridine	2.242	178.9	**CICQIQ**	Pyridine	2.259	179.0
**YOFLAJ**	Pyridine	2.244	180.0	**CICQIQ01**	Pyridine	2.259	180.0
**YOFKOW01**	Pyridine	2.245	176.4	**HUMMAD**	Pyridine	2.259	180.0
**HUMMAD01**	Pyridine	2.246	180.0	**DOVYOE01**	Pyridine	2.260	180.0
**YOFKIQ**	Pyridine	2.247	177.8	**HUMMAD**	Pyridine	2.261	180.0
**DOVZAR01**	Pyridine	2.248	178.6	**DOVYUK**	Pyridine	2.262	180.0
**YOFKIQ**	Pyridine	2.248	177.2	**OVANEG**	Pyridine	2.262	176.2
**MIYJIP**	Pyridine	2.250	177.7	**OVANEG**	Pyridine	2.264	175.2
**DOVYUK**	Pyridine	2.250	180.0	**HUMMAD01**	Pyridine	2.264	177.8
**DOVZAR**	Pyridine	2.251	178.8	**HINXOR**	R_3P=N-I-R	2.265	176.8
**PYRIDI01**	Pyridine	2.251	180.0	**CICQIQ03**	Pyridine	2.268	180.0
**LUKZOI**	Pyridine	2.252	180.0	**NOMCAW**	Pyridine	2.275	175.3
**YOFKIQ**	Pyridine	2.253	180.0	**NOMBOJ**	Pyridine	2.278	175.4
**NOMCAW**	Pyridine	2.254	177.0	**BAZNAR04**	Pyridine	2.278	180.0
**CICQIQ02**	Pyridine	2.254	180.0	**HINXIL**	R_3P=N-I	2.279	176.1
**DOVZAR02**	Pyridine	2.254	178.0	**BAZNAR04**	Pyridine	2.280	180.0
**YOFKEM**	Pyridine	2.254	178.3	**KABRUB**	R_3P=N-I	2.285	180.0
**LULBIF**	Pyridine	2.255	178.7	**CICQOW**	Pyridine	2.288	178.9
**DOVYUK**	Pyridine	2.256	177.5	**CICQOW02**	Pyridine	2.294	178.1
**HUMMAD04**	Pyridine	2.256	177.8	**DEFXIW**	Pyridine	2.294	180.0
**DOVYOE01**	Pyridine	2.256	177.7	**CICQOW01**	Pyridine	2.297	179.1
**HUMMAD01**	Pyridine	2.256	180.0	**GANXEZ**	Pyridine	2.300	180.0
**HUMMAD04**	Pyridine	2.256	180.0	**HMTITI**	HMTA	2.303	176.5
**OVANAC**	Pyridine	2.257	178.0				
